# Molecular Principles of Gene Fusion Mediated Rewiring of Protein Interaction Networks in Cancer

**DOI:** 10.1016/j.molcel.2016.07.008

**Published:** 2016-08-18

**Authors:** Natasha S. Latysheva, Matt E. Oates, Louis Maddox, Tilman Flock, Julian Gough, Marija Buljan, Robert J. Weatheritt, M. Madan Babu

**Affiliations:** 1 MRC Laboratory of Molecular Biology, Francis Crick Avenue, Cambridge CB2 0QH, UK; 2 Department of Computer Science, University of Bristol, Bristol BS8 1UB, UK; 3 The Donnelly Centre, University of Toronto, Toronto, ON M5S 3E1, Canada

**Keywords:** gene fusion, fusion protein, cancer genomics, protein interaction networks

## Abstract

Gene fusions are common cancer-causing mutations, but the molecular principles by which fusion protein products affect interaction networks and cause disease are not well understood. Here, we perform an integrative analysis of the structural, interactomic, and regulatory properties of thousands of putative fusion proteins. We demonstrate that genes that form fusions (i.e., parent genes) tend to be highly connected hub genes, whose protein products are enriched in structured and disordered interaction-mediating features. Fusion often results in the loss of these parental features and the depletion of regulatory sites such as post-translational modifications. Fusion products disproportionately connect proteins that did not previously interact in the protein interaction network. In this manner, fusion products can escape cellular regulation and constitutively rewire protein interaction networks. We suggest that the deregulation of central, interaction-prone proteins may represent a widespread mechanism by which fusion proteins alter the topology of cellular signaling pathways and promote cancer.

## Highlights

• Parents of fusion proteins occupy central positions in protein interaction networks
• Parents are rich in interaction-mediating features, which are often lost via fusion
• Fusions preferentially join proteins with no previous connection in protein networks
• Fusion proteins escape regulation by losing post-translational modification sites

## Graphical Abstract

**Figure:**
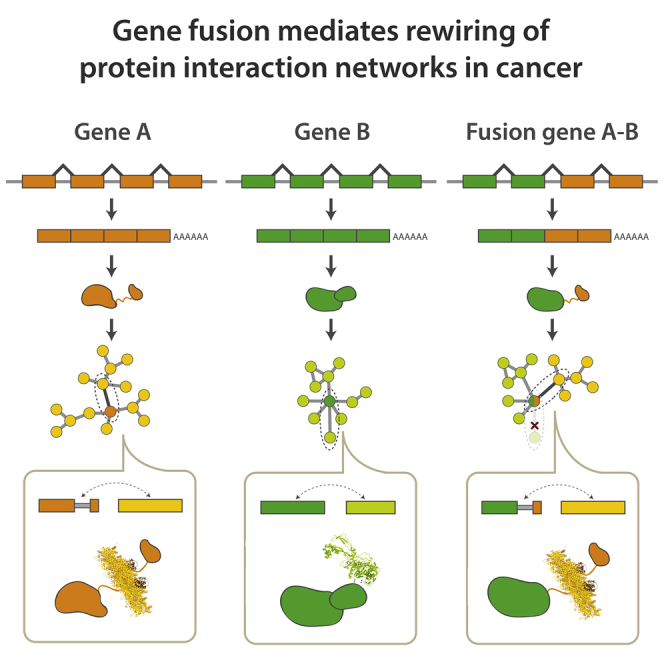

## Teaser

The molecular mechanisms of fusion-mediated interactome disruption are currently unclear. Latysheva et al. find that fusion-forming proteins occupy central positions in interaction networks. They lose much of their extensive interaction-mediating ability and capacity for regulation upon fusion. These findings provide insights into how fusion proteins could rewire networks in cancer.

## Introduction

Fusion genes are hybrid genes formed from two previously independent parent genes. Historically, gene fusions have been viewed as common driver mutations in malignancies associated with blood, lymph, and bone marrow tissue, but are becoming increasingly recognized as important players in solid tumors (Mertens et al., 2015a, Mertens et al., 2015b, Yoshihara et al., 2015). For example, translocation-induced gene fusions are found in about 90% of all lymphomas and over half of all leukemias (Lobato et al., 2008), and the *TMPRSS2-ERG* fusion is the most frequent genetic aberration in prostate cancer (Nam et al., 2007). In accord with their important role in oncogenesis, fusion transcripts and proteins have been utilized in many areas of clinical care, from biomarker development and diagnostics to acting as therapeutic targets (Kumar-Sinha et al., 2015, Mertens et al., 2015b). Yet, aside from a relatively small number of well-studied fusions, the functions of fusion proteins and the cellular context in which they operate remain unclear.

A variety of mechanisms can lead to the fusion of two genes, such as insertions, deletions, inversions, and translocations. Continuous transcription of neighboring genes (Varley et al., 2014) or *trans*- and *cis*-splicing of pre-mRNAs (Jividen and Li, 2014, Zhang et al., 2012) can also generate fusion transcripts and proteins. If fusion transcripts are translated, the resulting fusion proteins have the potential to redirect cellular signaling pathways and act as principal oncogenic drivers (see Watson et al., 2013, Yoshihara et al., 2015). Despite some concerns over whether certain putative fusion mRNAs may be artifacts of the sequencing procedure (Yu et al., 2014), the widespread finding of recurrent gene fusions in tumor samples, the clinical utility of an increasing number of gene fusions, and a growing body of literature on fusion protein functionality adds support to their potential for significant biological impact.

**Figure 1:**
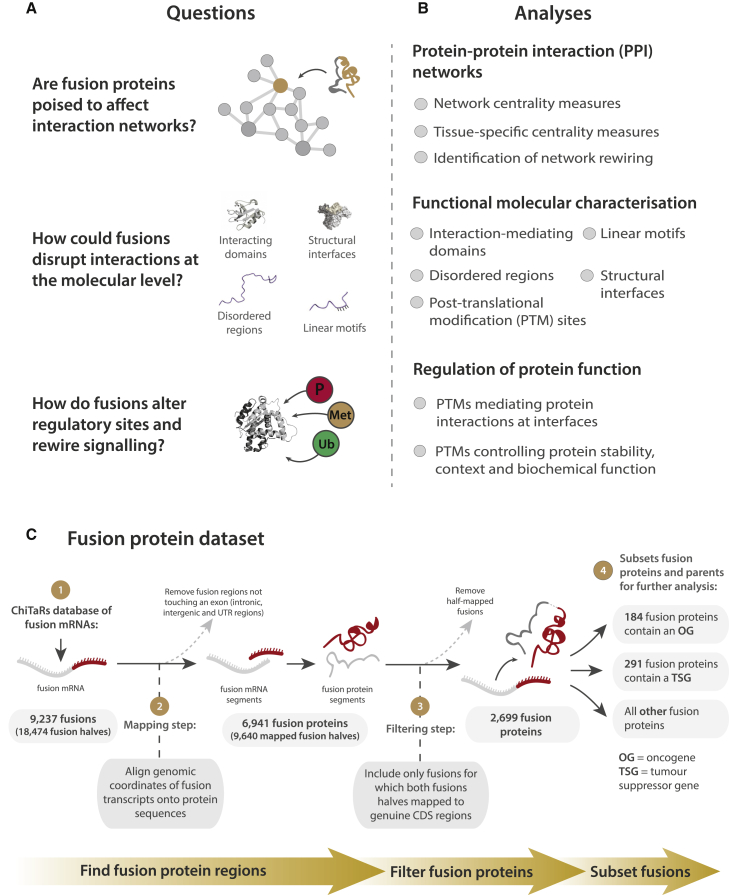
Study Outline (A) Investigating how gene fusions and fusion proteins could affect molecular interactions in cancer. (B) Summary of analyses employed. (C) Description of processing procedure applied to the ChiTaRS database of fusion (“chimeric”) mRNA sequences to obtain a data set of fusion proteins. See also Figures S1 and S2 and Table S1.

There are now approximately 10,000 known gene fusions, most of which have only recently been discovered using deep sequencing technology (Mertens et al., 2015a). The molecular functions of gene fusions, and the fusion proteins they encode, remain relatively poorly understood. Recent bioinformatics work on gene fusions (reviewed in Latysheva and Babu, 2016) has examined fusion protein domain content and recombination, reading frame conservation, intrinsic disorder at fusion junctions, and expression properties. However, the molecular principles of fusion-mediated rewiring of protein networks and how fusion proteins could disrupt native protein interactions remain unclear. Here, we devise a genome-scale computational data analysis framework to investigate the molecular principles by which fusion proteins affect protein interactions (Figures 1A and 1B). Understanding the structural features of fusion proteins, as well as the interactions that are recurrently disrupted or created as a result of fusion, will help clarify how fusions contribute to specific cellular phenotypes and influence cancer initiation and progression.

## Results

To compose a set of human fusion proteins, a list of fusion transcripts from the ChiTaRS v1 database (Frenkel-Morgenstern et al., 2013) was acquired and mapped onto Ensembl protein sequences (Experimental Procedures; Figure 1C). In this study, only fusions affecting protein-coding regions were examined. In total, we mapped 2,699 distinct fusion proteins derived from 3,279 genes (Table S1; fusion protein mappings are available as a web resource at http://fusion.d2p2.pro, integrated into the D2P2 database; Oates et al., 2013). Genes that form fusions (“parent genes”) are enriched for functions related to translation, mRNA splicing, and the cell cycle, and for protein classes related to translation, acetyltransferase activity, and the binding of actin, chromatin, and RNA (Table S1). Parent genes that form multiple fusions, especially five or more, are further enriched for functions relating to translation, RNA binding, and nucleic acid binding.

Gene fusion events can be summarized as a network, in which nodes indicate genes and a link between nodes indicates the occurrence of a fusion between genes. Our resulting network of gene fusions involving 3,209 genes (as gene symbols; Figure S1A) expands upon previous networks of ∼300 gene fusions (Höglund et al., 2006, Mitelman et al., 2007); we confirm the presence of several major hubs, i.e., nodes with many edges (e.g., *MLL*, *ETV6*, *NUP98*, *EWSR1*, and *ALK*), and highlight novel fusion hubs (e.g., *COL1A1*, *HSP90AA1*, *MT1A*, *NCL*, and *AFF1*; Table S1; Figure S1A). The number of fusions formed for each gene follows a power law distribution (Figure S1B), with most parent genes forming few fusions (e.g., only 21 genes form ten or more fusion proteins). Over a third of known oncogenes (OGs) and a quarter of known tumor suppressor genes (TSGs) form fusions in this data set (Figure S1).

### Parent Proteins Have More Central Roles in Protein Interaction Networks and Are Expressed at Higher Levels

**Figure 2:**
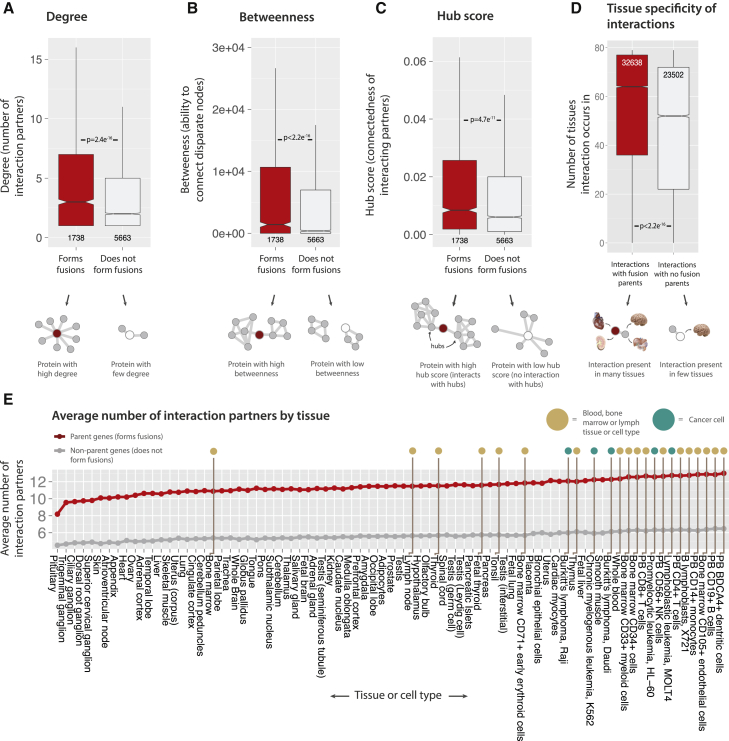
Network Centrality of Parent Genes and Proteins (A–C) Parent genes possess more interaction partners in PPI networks (A), have higher betweenness centrality (B), and higher hub scores (C). (D) PPIs involving parent proteins occur in more human tissues than interactions not involving parent proteins. (E) The average number of interaction partners for parent proteins and all other proteins by tissue or cell type (gold = blood, bone marrow, and lymph tissues and teal = cancer cells). Throughout this study, distribution outliers are excluded from boxplots for presentation purposes, but included in statistical analyses. See also Figures S3 and S4 and Tables S2 and S3.

To examine whether parent genes encode proteins with central positions in the human interactome, a high-confidence data set of human protein-protein interactions (PPIs) (Wang et al., 2012) was analyzed. In addition to a much higher number of interaction partners (node degree; Figure 2A), parent proteins have a significantly higher tendency to interconnect interaction clusters, as quantified by betweenness centrality, which measures the extent to which a given node in a network lies on the shortest paths between all other nodes (Figure 2B). Furthermore, parent proteins have higher Kleinberg’s hub scores (see Experimental Procedures), which measure a protein’s connection to network hubs (Figure 2C). Compared to central non-parents, the most central parent proteins were more likely to be involved in functions such as mRNA splicing, cell proliferation, DNA replication, and repair (Table S2).

We observed that parent mRNAs and proteins are more abundant compared to non-parents (∼3-fold difference between averages; Figures S2A and S2B) in medulloblastoma cell lines (Vogel et al., 2010). Additionally, parent proteins have very similar half-lives to non-parent proteins (Figure S2C). Further, by integrating data on 12 oncogenic signaling blocks (Cui et al., 2007), we find that parent proteins are over twice as likely to be involved in signaling processes implicated in oncogenesis (χ^2^ = 29.5, df = 1, and p = 5.7 × 10^−8^) (Figure S2D) and are over 2.5 times as likely to be genes essential for cellular viability (χ^2^ = 396.8, df = 1, and p < 2.2 × 10^−16^) (Figure S2E). Although these trends need to be analyzed in different tissues, these results suggest that altering parent proteins could have a major effect on critical cellular functions and for a sustained period of time.

Parent genes were grouped into OG parent genes, TSG parent genes, and all other parent genes (Figure S3A). Parent genes that are neither OGs nor TSGs possess significantly higher network centrality than non-parent genes, indicating that centrality is a feature of parent genes more broadly and not simply reflective of the centrality of OGs and TSGs. Further, parent OGs and TSGs tend toward higher centrality than non-parent OGs and TSGs, respectively (Figure S3B). For example, average centrality measures for parent TSGs are approximately 30% higher than non-parent TSGs. Replicate network centrality calculations on two additional PPI data sets—the consensus network used in further analyses (see below; Bossi and Lehner, 2009) (Figure S3C) and an unbiased interaction network derived using mass spectrometry (Huttlin et al., 2015) (Figure S3D)—were consistent with those described above.

### Parent Proteins Have Higher Centrality in the Interaction Networks of Cancer-Associated Cell and Tissue Types

Next, the role of parent proteins in tissue-specific protein interaction networks (Bossi and Lehner, 2009) was examined. PPIs involving parent proteins are present in more human tissues (median of 64 of 79 tissues, compared to 52 of 79 for non-parents; p < 2.2 × 10^−16^; Figure 2D), indicating that fusion events do not only affect tissue-specific interactions. Parent proteins consistently have on average ∼5 additional interaction partners across most tissues (Figure 2E). Interestingly, the tissues and cell types with the highest degrees for parent proteins—e.g., B and T cells, bone marrow cells, and blood cells—are cell types often associated with fusion-induced cancers (gold dots, Figure 2E). Furthermore, parent proteins in the five cancer cell types in the data set (teal dots) have on average 9.1% higher degree than non-cancer cells and 12.1% higher degree than the set of non-cancer and non-blood/bone/lymph cell types (Table S3). This trend is not observed for betweenness (Figure S3E), but is for hub scores (Figure S3F), which may indicate that gene fusions in cancer may preferentially affect nodes of high degree (either directly or indirectly) rather than alter global network cohesion. Fusions could therefore be especially disruptive in tissues with interaction networks containing proteins with unusually high degree. Finally, fusion transcripts detected in cell lines of metastatic tumor origin may have parent genes with higher centrality compared to those from primary tumors (Figures S4A–S4D; Supplemental Information), suggesting a possible connection between cancer aggressiveness and parent centrality. Although this trend was not observed in the mass spectrometry PPI data set (Huttlin et al., 2015; data not shown), the concept of a link between cancer stage and the roles of parent proteins in PPI networks may be relevant in specific contexts (e.g., certain cancer types).

### Parent Proteins Are Unstructured and Enriched for Interaction-Mediating Domains, which Are Preferentially Excluded from Fusion Proteins

**Figure 3:**
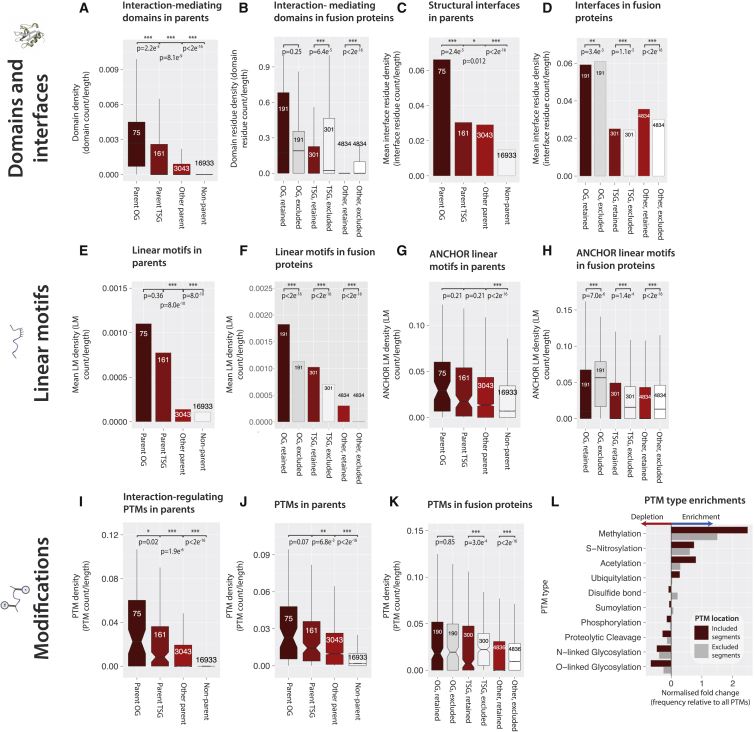
Interaction-Mediating Molecular Features in Fusion Proteins (A and B) IMDs in parent proteins (A) and fusion proteins (B). (C and D) The PPI interface residues in parent proteins (C) and fusion proteins (D) are shown. (E and F) The ELM LMs in parent proteins (E) and fusion proteins (F) are shown. (G and H) The predicted ANCHOR LMs in parent proteins (G) and fusion proteins (H) are shown. (I) The putative interaction-regulating PTMs in parent proteins are shown. (J and K) Other PTM sites in parent proteins (J) and fusion proteins (K) are shown. (L) The PTM type enrichments in included and excluded parent protein segments are shown. Within each subplot, Holm’s sequential Bonferroni correction for multiple testing was applied. See also Figure S5 and Tables S4 and S6.

The structural features of parent proteins and their retention within fusion proteins were investigated (Figures 3A–3L and S5A–S5K). In agreement with a previous study (Hegyi et al., 2009), parent proteins in our expanded data set (3,279 parent proteins versus 406) have significantly higher intrinsic structural disorder scores than non-parents (Figure S5A): OG parents have on average 1.27 × (0.39 versus 0.31; p = 2.8 × 10^−4^; and pairwise Wilcoxon rank-sum tests with Holm multiple testing correction), TSG parents 1.15 × (p = 1.5 × 10^−3^), and other parents 1.13 × (p < 2 × 10^−16^) higher disorder compared to non-parents. Parent OGs and TSGs are approximately equally disordered as non-parent OGs and TSGs (Figure S5B), as are included versus excluded fusion protein segments (Figure S5C). This suggests that any observed enrichment of linear motifs and post-translational modifications (PTMs) in included segments (see below), which are features correlated with disorder (Davey et al., 2012), are not simply due to included segments being more disordered. Throughout the structural feature calculations, densities instead of counts are used to control for protein length.

Using a database of PPIs defined at the structurally resolved level of domains (Meyer et al., 2013), we investigated parent versus non-parent densities of interaction-mediating domains (IMDs). Parent proteins, especially OG and TSG parent proteins, have higher densities of IMDs (Figure 3A). On average, compared to non-parent proteins, OG parents have 4.6×, TSG parents 2.7×, and other parents 1.5× the IMD densities (all corrected p values: <2.2 × 10^−16^). There is a slight tendency for parent OGs to have higher IMD densities than non-parent OGs (on average 1.3×; p = 9.1 × 10^−3^; Figure S5D). Hence, although parent proteins are generally more intrinsically disordered, they are also enriched in structured domains that mediate protein interactions. IMDs tend to largely be excluded from fusion proteins (Figure 3B; Table S4). OG parent proteins, in contrast to TSG and other parent proteins, tend to retain IMDs upon fusion. Overall, the most frequently retained IMDs include RNA-recognition, tyrosine kinase, pleckstrin homology (signaling and cytoskeleton), and SH3 and SH2 signaling domains (Table S4). The average level of domain truncation upon transfer varies significantly by domain type, and the most intact IMDs which occur ≥10 times include ubiquitin conjugating domains, the ubiquitin-like PB1 domain (a specificity adaptor to kinases), and the proliferation modulating S_100 domain. Parents that repeatedly donate large portions of IMDs are enriched for functions in translation, cell structure morphogenesis, and cell cycle and protein modification (Table S4).

### Transfer of IMDs Can Create Novel Interactions and Preserve Important Natural Interactions

**Figure 4:**
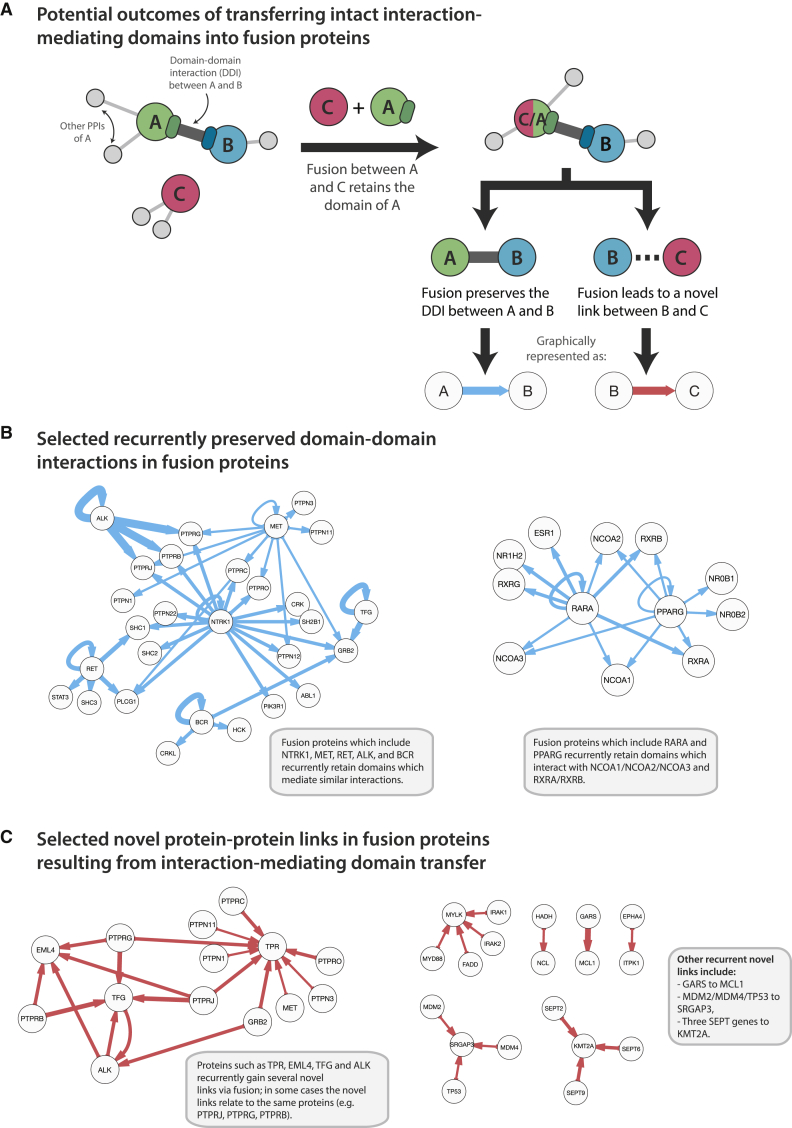
Retained and Novel PPI in Fusion Proteins (A) The repeated inclusion of large portions of specific IMDs in fusion proteins can lead to the retention of domain-mediated interactions or the creation of novel interaction-like links between proteins. (B and C) Subsets of the recurrently retained domain-mediated PPIs (B) and novel links (C) are shown. See also Figure S6 and Table S5.

The repeated inclusion of large portions of specific IMDs in fusion proteins is interesting for two reasons (Figure 4A). First, it can point to the importance of a particular domain-domain interaction (DDI) for a fusion protein’s function. Second, as a result of the fusion, a novel interaction-like link can occur between the interaction partner of the included domain and the fusion partner. We map which domain-mediated PPIs are repeatedly conserved in fusion proteins (Figures 4B and S6A; Table S5). We find that 192 IMD-mediated PPIs are recurrently retained in fusion proteins and comment on the most frequently conserved DDIs (see the Figure S6A legend).

We also map novel protein links that are created through IMD transfer (Figures 4C and S6B; Table S5). A protein interaction “link” was drawn between proteins A and B if there existed some fusion protein B-C, where C normally interacts with A and at least 90% of C’s IMD was retained (Figure 4A). Of the 126 novel links, 116 (92%) do not normally occur in the cell. The most frequent novel links include many connections for BCR, with the newly linked proteins being enriched for functions in cell proliferation and cellular component movement (Table S5), and 11 new connections for the nuclear trafficking protein TPR, including eight tyrosine protein phosphatases (Figures 4C and S6B). Certain fusion-induced novel links are recurrent, e.g., fusion proteins involving both EML4 and TFG lead to the gain of similar links (i.e., connections to receptor-type protein tyrosine phosphatases PTPRB, PTPRG, and PTPRJ).

### Fusion-Generated Novel Links Disproportionately Connect Proteins that Are Distant in the Interaction Network

We examined the distance between the protein pairs in the novel links set in a non-diseased PPI network. Where a path existed between the novel links pairs, the distance was overall slightly shorter than in other protein pairs in the network (Figure S6C). However, fusion was found to disproportionately connect proteins which normally reside in separate sections of the interactome, whereas only 10.7% of protein pairs in the PPI network had no connecting path, 29.3% of protein pairs in the novel links set had no previous connecting path (Fisher’s exact test on contingency table, odds ratio = 3.47, p = 3.0 × 10^−8^) (Figure S6D). We examine the 34 newly connected protein pairs in Figure S6E (see the legend).

### Independent Structural Evidence Supports the Potential of Fusion Proteins to Disrupt PPI, Protein-RNA Interactions, and Protein-DNA Interactions

Structural interfaces in fusion proteins were identified by analyzing the Protein Interfaces, Surfaces, and Assemblies (PISA) database, which houses macromolecular interfaces (involving proteins, RNA, and DNA) in the Protein Data Bank (PDB). Parent proteins in the PDB contain more interface-forming residues (Figure 3C). On average, 1.5% of residues in non-parents form interfaces, and OG parents have on average 4.5×, TSG parents 2.1×, and other parents 2.0× this PISA residue density. Parent OGs have 2.4× the average interface residue density of non-parent OGs (p = 3.6 × 10^−5^; Figure S5E). Interface residue densities on included and excluded segments of parent proteins are similar (Figure 3D), though the distribution is skewed toward exclusion (Figure S5F). The 302 parent proteins which donate ten or more interface-forming residues to fusion proteins are enriched for functions relating to cell cycle signaling, carbohydrate and lipid metabolism, cellular component morphogenesis, and cell death (Table S6).

### Parent Proteins Are Enriched in Interaction-Mediating Short Linear Motifs, which May Be Preferentially Excluded from Fusion Products

Linear motifs (LMs) are short sequence motifs, usually <10 residues, often found in intrinsically disordered regions (Tompa et al., 2014). Using 1,410 experimentally validated LMs from the ELM database (Dinkel et al., 2014) and over a million putative LMs identified using the ANCHOR program (Dosztányi et al., 2009), we tested for enrichment of LMs within parent proteins compared to all other proteins. Parent proteins have more experimentally verified LMs on average (Figure 3E), with OG and TSG parents harboring more motifs. Although most parents have zero experimental LMs due to the small size of this data set, on average, OG parents have 10.1× (p < 2 × 10^−16^), TSG parents 7.1× (p < 2 × 10^−16^), and other parents 1.3× (p = 8.0 × 10^−10^) the LM density of non-parents. Parent TSGs have slightly higher LM densities compared to non-parent TSGs (Figure S5G). Fusion proteins tend to retain ELM LMs, as shown by higher mean LM densities in included segments (Figure 3F). Parent proteins, which donate ELM LMs, function in the regulation of cell death, the stress response, protein metabolism, and nucleic acid binding (Table S6). Similarly, the expanded ANCHOR data shows higher densities of LMs in parents (Figure 3G), though parent OGs and TSGs have similar densities to the non-parent categories (Figure S5H). Interestingly, the larger ANCHOR data set shows a strong trend toward the exclusion of LMs (Figure 3H). Either trend implies that fusion substantially disrupts transient interactions mediated by LMs.

### PTMs that Regulate Protein Interactions Are Enriched in Parent Proteins

We mapped putative interaction-regulating PTMs (PTMcode v2 database; Minguez et al., 2015) onto proteins and found that compared to non-parents, OG parents have on average 4.6×, TSG parents 3.5×, and other parents 2.2× the PTM density (all corrected p < 2 × 10^−16^; Figure 3I). Parent TSGs have slightly more interaction-regulating PTMs compared to non-parent TSGs (1.5×, p = 0.03; Figure S5I). These PTM sites overall tend toward exclusion from fusion proteins (Figure S5J), though the retention and loss is comparable in OG and TSG parents.

### Parent Proteins Are Enriched in PTM Sites, and Fusion Proteins Tend to Selectively Escape Regulation by PTMs

In addition to regulating protein interactions, post-translational and co-translational modification sites can regulate protein stability (e.g., by ubiquitination), subcellular localization (e.g., N-myristoylation), and protein function (e.g., acetylation). Parent proteins have significantly more PTMs (Figure 3J) compared to non-parents (on average 0.009 PTMs/residue): OG parents have 3.5×, TSG parents 3.5×, and other parents 2.3× the PTM densities of non-parents (all corrected p < 2 × 10^−16^). This suggests that the function, stability, and subcellular location of parent proteins are extensively regulated by PTMs. Further, on average, parent OGs have 1.5× (p = 7.5 × 10^−3^) the PTM content of non-parent OGs, and parent TSGs have 2.1× (p = 1.4 × 10^−5^) the PTM content of non-parent TSGs (Figure S5K). PTMs are generally excluded from fusion proteins, though not in OG parents (Figure 3K). The selective exclusion of PTM sites suggests that fusion proteins tend to escape regulation by signaling pathways. TSG parents experience the heaviest loss of PTMs, with excluded segments having over triple the median PTM density of included segments (excluded: 0.022 PTMs/residue; included: 0.007; p = 3.0 × 10^−4^; Figure 3K). Parent proteins which retain at least 90% of their PTM content are enriched for functions in translation, ion transport, and metabolism (Table S6), while parent proteins which lose at least 90% of their PTMs have a wide range of functions, including splicing and cell matrix adhesion.

Next, we examined the PTM profiles in included and excluded fusion protein segments (Experimental Procedures; Figure 3L). Certain PTM types (e.g., S-Nitrosylation) occur in either parental segment more frequently than expected given the global frequencies of all PTMs in dbPTM, while other PTM types (e.g., methylation and acetylation) showed marked presence/absence patterns based on segment inclusion (Table S6).

### Fusion Can Lead to the Gain and Loss of Ubiquitination Sites, which May Deregulate the Activity of OGs and TSGs

**Figure 5:**
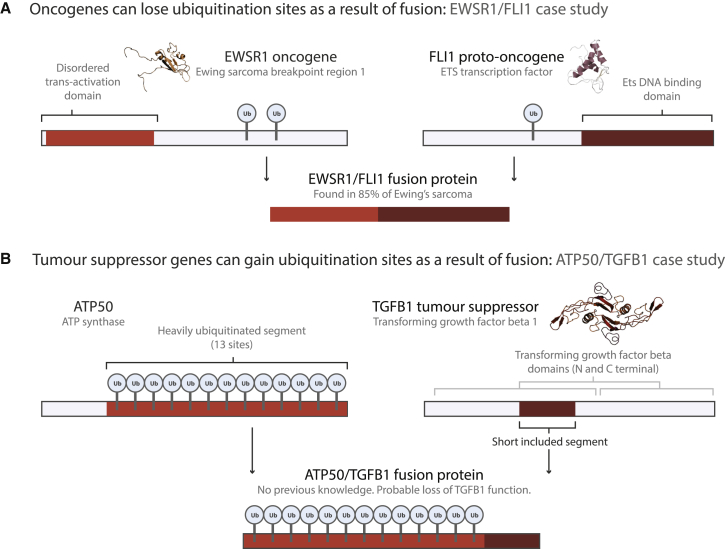
Fusion-Induced UB Site Gain and Loss in Cancer-Associated Proteins Fusion proteins involving OGs and TSGs can lead to the loss or gain of ubiquitination sites. (A) Example of an OG losing UB sites upon fusion. (B) Example of a TSG gaining UB sites upon fusion. The protein structure cartoons are of EWSR1 (PDB: 2CPE), FLI1 (PDB: 1FLI), and TGFB1 (PDB: 1KLA).

Ubiquitination (UB) sites are of particular interest since their loss and gain upon fusion could “upregulate” OG activity or “downregulate” TSG activity, due to the role of UB sites in mediating protein stability and degradation. We find 14 fusion proteins in which OGs lose ≥5 UB sites and ten fusion proteins in which a TSG gains ≥5 UB sites (Table 1). As an illustrative example, we profile the well-known *EWSR1-FLI1* gene fusion from Ewing’s sarcoma (Figure 5A). The specific pattern of segment retention in EWSR1-FLI1 fusion proteins leads to UB site loss, which may confer increased stability onto the fusion product, adding to the known oncogenic mechanism of transcriptional deregulation. Notably, decreased UB-mediated degradation of ETS family transcription factors (e.g., FLI1) has been linked to cancer (Vitari et al., 2011). Conversely, one of the most extreme examples of UB site gain by a TSG occurs in the previously unstudied *ATP50-TGFB1* fusion (Figure 5B), which results in the amalgamation of a heavily ubiquitinated segment with a short portion of the TGFB1 tumor suppressor domain, hinting at a fusion-mediated loss of TSG function. TGF-β signaling is known to inhibit cell proliferation and is normally tightly regulated by UB (Huang and Chen, 2012). OG parents do not lose and TSG parents do not gain UB sites more often than expected (data not shown), but individual cases identified here (Table 1) could be of substantial biological interest for follow-up studies.

### Fusions Involving Transcription Factors Are Linked to Significant Alterations in Downstream Target Gene Expression Levels

To investigate the potential downstream network rewiring effects due to fusion events, we investigated whether fusions involving transcription factors (TFs) are associated with downstream expression changes in the TFs’ regulatory targets. TCGA tumor samples with TF-containing fusion transcripts and paired normal controls were identified (Experimental Procedures). The regulatory target genes of TFs were acquired from the TRRUST database (Han et al., 2015). Differential gene expression (DGE) values were calculated (absolute log_2_ fold change between diseased and healthy samples). The targets of TFs had significantly (i.e., corrected p < 0.05) higher DGE values in five of the eight paired breast cancer samples when compared to all other genes (Figure S7). For example, four fusion transcripts containing TFs were detected in patient TCGA-GI-A2C9; these four TFs together affected 51 mapped regulatory targets, the mean (absolute log_2_) DGE of which is 2.0× the mean DGE of all other genes (Table S7; corrected p = 9.6 × 10^−5^). Across the eight available biospecimen pairs, the average DGE of TF targets is 1.41× (mean) and 1.45× (median) the DGE of all other genes.

## Discussion

**Figure 6:**
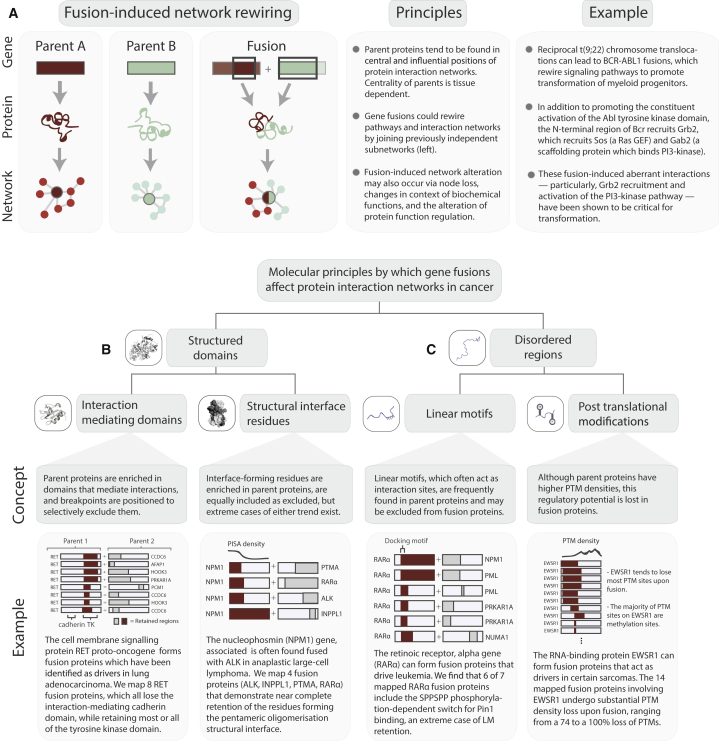
Molecular Principles by which Gene Fusions Can Alter Protein Interaction Networks in Cancer (A) Fusion tends to involve highly central proteins in interaction networks and can alter networks by several mechanisms. Rewiring effects can play key roles in seemingly straightforward fusion events, as in the constitutive kinase activation found in the BCR-ABL1 fusion. (B and C) More generally, fusion can affect molecular interactions of proteins by shuffling interaction-prone regions within ordered (B) and disordered (C) protein segments. See also Figure S7 and Table S7.

Many disease states result from altered dynamics of complex regulatory and signaling interactions. Representing interactions as networks provides a conceptual framework for understanding how mutations in proteins can affect entire cellular systems and cause disease (Wang et al., 2011, Wu et al., 2010), especially when combined with structural analyses of interacting proteins (Sudha et al., 2014, Wang et al., 2012). Here, we investigated the interaction properties and structural features of thousands of putative fusion proteins. Based on our observations, we delineate genome-scale molecular principles by which gene fusions can affect protein networks, rewire signaling pathways, and contribute to disease (Figure 6). These trends will be useful for setting novel gene fusions into context, building on the performance of previous driver gene fusion prioritization algorithms (Abate et al., 2014, Shugay et al., 2013), and interpreting studies of fusion protein functionality.

### Fusion Preferentially Affects Highly Central, Interaction-Prone Proteins

Although it is likely that not all of the analyzed fusion proteins drive disease (e.g., genomic instability can produce passenger fusions; Mertens et al., 2015a), parent proteins are nonetheless enriched for a wide variety of interaction-prone elements, such as IMDs, interface-forming residues, LMs, and PTM sites that regulate PPIs. The observed density of interaction-mediating features in parent proteins is in accord with their centrality in interaction networks. These results are consistent with other computational work on disease mutations, which have shown that disease-related in-frame mutations (Wang et al., 2012) and disease-causing non-synonymous single nucleotide polymorphisms (David et al., 2012) are preferentially located on PPI interfaces. Finally, the finding that many parent genes are essential genes dovetails with the concept of “edgetic” perturbations in cancer, i.e., mutations that disrupt specific interactions (or edges) of proteins rather than the entire node (Charloteaux et al., 2011, Rolland et al., 2014, Wang et al., 2015), given that disrupting essential genes is associated with lethality, fusion may offer an opportunity to disrupt only a portion of an essential protein’s function, such as specific interactions.

Network disruption may play a role in fusion proteins that first appear to have relatively simple mechanisms of oncogenesis (Figure 6A), for example, the concurrent rewiring of signaling pathways can be critical for BCR-ABL1 mediated transformation (Pawson and Warner, 2007). Importantly, targeting the interacting partners or downstream signaling of fusion proteins could be a fruitful area for therapeutic agent development (see Tognon et al., 2011). In this context, our observation that TF fusions significantly perturb target gene expression in breast cancer lends further weight to the signaling perturbation capabilities of fusion events.

### Fusion Results in a Loss of Parental Interaction-Mediating Features and Regulatory Sites

Although parent proteins are enriched for interaction-mediating features, the segments of parents that are included within fusion proteins appear to be depleted of functional regions (though OG parents retain more of these features than other parents). Examining specific cases of fusion-mediated loss and gain of molecular features (Figures 3A–3L), as well as interaction preservation and creation (Figures 4A–4C), is a rich resource for hypothesis generation. For example, fusion proteins characterized by the repeated inclusion of largely complete tyrosine kinase domains (e.g., Figure 6B) could be promising targets for kinase inhibitors. Proteins dependent on the function of several distinct molecular features (such as the interface residues and nuclear import/export signal motifs in nucleophosmin; Figure 6B), as well as proteins sensitive to changes in PTM content (such as EWSR1; Figure 6C), may be especially disrupted by fusion events.

Although we largely addressed each interaction-mediating and regulatory molecular feature of parent and fusion proteins separately, these entities are not independent. For instance, LMs tend to form interactions conditionally on PTM site status (Van Roey et al., 2013). For example, the retinoic receptor alpha gene (*RARα*) encodes a LM that acts as a phosphorylation-dependent switch for binding Pin1. RARα forms driver fusion proteins in acute promyelocytic leukemia, for which Pin1 suppression is used as a treatment (Gianni et al., 2009). We find a RARα fusion protein that excludes the LM in question (Figure 6C), which could correspond to a treatment resistant patient. Knowledge of the specific retained sequence of fusion proteins has previously been observed to be key to patient treatment (Robinson et al., 2011).

### Conclusions

Our findings demonstrate that proteins that form fusions tend to be highly interactive and positioned in critical regions of PPI networks. Disruption of such proteins may alter the topology of signaling and regulatory pathways of cells and promote cancer. A detailed understanding of the molecular impact of the rewired network will be helpful for future drug discovery studies. For example, in cases where driver fusion proteins retain the ability to form interactions, their carcinogenic activity could be reduced by the targeted disruption of specific interaction interfaces with small molecules (Cierpicki and Grembecka, 2015, Jin et al., 2014, Kuenemann et al., 2015). Additionally, recent methodological advances in therapeutically degrading specific proteins in vivo (Bondeson et al., 2015, Winter et al., 2015) could be instrumental to targeting oncogenic fusion proteins that have escaped normal regulatory pathways.

## Experimental Procedures

### Database Identification, Processing, and Integration

To compose a set of human fusion proteins, we acquired a database (ChiTaRS v1 database; Frenkel-Morgenstern et al., 2013) of 9,237 fusion mRNAs. The fusion transcripts were mapped onto known proteins in the Ensembl database using ChiTaRS genomic coordinates and segments that mapped to non-exonic regions (intronic, UTR, or intergenic sequences) were discarded. The resulting data set maps all fusion protein segments defined at the DNA/gene, mRNA, and protein levels (Table S1). We limit our analysis to fusion proteins in which both parents were mapped to known Ensembl proteins. Fusion protein mapping information is made available via a web server (http://www.fusion.d2p2.pro). A fusion network of all gene fusions was constructed using Cytoscape. Throughout this study, gene sets were tested for enrichments of GO-Slim molecular functions and protein classes using PantherDB (Mi et al., 2013). See the Supplemental Information for further methodological details.

### mRNA and Protein Abundance and Half-Lives of Parents

Protein and mRNA abundances were acquired from a microarray and shotgun proteomics study performed on the Daoy medulloblastoma cell line (Vogel et al., 2010), and protein half-life data were taken from a SILAC study in HeLa cells (Boisvert et al., 2012). These data sets were overlapped onto parent and non-parent gene sets, and differences in distributions of abundance and half-life by category were quantified by non-parametric Wilcoxon rank-sum tests.

### Parent Gene Participation in Oncogenic Signaling Blocks

Disproportionate parent protein participation in cancer signaling processes (Cui et al., 2007) was assessed using a contingency table and a chi-square test of independence.

### Parent Gene Essentiality

1,734 “core” essential genes shared between two cell lines (Blomen et al., 2015) were acquired and tested for enrichment among parent genes as above.

### PPI Network Centrality

Network centrality calculations for both parent and non-parent genes/proteins were performed on a non-tissue specific PPI network (Wang et al., 2012) using the igraph R package. See the Supplemental Information for definitions of centrality measures. A tissue-specific PPI network (Bossi and Lehner, 2009) was acquired in order to calculate tissue-specific PPI metrics (Buljan et al., 2012). A more recent, expanded, and unbiased protein interaction data set from human cells (Huttlin et al., 2015) was also investigated.

### Intrinsic Structural Disorder in Parent Proteins

Residue-by-residue predictions for disorder for each protein in the human proteome were generated using the IUPred program (Dosztányi et al., 2005; http://www.iupred.enzim.hu/). Scores range from 0 to 1, where higher scores indicate a higher propensity toward intrinsic disorder. Intrinsic disorder was calculated for genes (i.e., longest isoform Ensembl protein) and for specific included and excluded segments as an average over either the protein or segment length.

### Analysis of Interacting Domains within Proteins

A data set of curated, structurally resolved PPIs was acquired (Meyer et al., 2013), and residues that form IMDs were mapped onto parent and non-parent proteins. IMD retention was quantified by calculating IMD residue densities on included and excluded segments. The frequency and completeness of retention of different domain types was summarized across the fusion protein set. Statistically significant differences between gene sets in the distributions of IMD residues were assessed as before. Parents which donate ≥20% of at least one IMD were analyzed for functional and protein class enrichments.

### Identifying Novel and Retained PPIs of Fusion Proteins

The above set of domain-mediating PPIs was analyzed to identify which PPIs are recurrently (two or more times) retained in fusion proteins. DDIs were deemed to be “retained” if at least one fusion protein incorporated at least 90% of the IMD. Novel interactions created as a result of the transfer of IMDs were between protein A and B if there existed at least one fusion protein B-C, where C normally interacts with A and at least 90% of C’s IMD was retained. Novel links were those that did not appear in a set of known PPIs (Wang et al., 2012).

### Identifying Shortest Path Distances between Proteins Newly Linked by Fusion

Pairwise shortest path lengths (geodesics) between all protein pairs in a PPI network (Wang et al., 2012) were calculated using igraph. The distribution of shortest path lengths in the novel link set was compared to the distribution of path lengths in 1,000 randomly sampled protein pairs from the complete geodesic matrix as before. Disconnected protein pairs had infinite shortest path lengths, reflecting the absence of a geodesic. A contingency table containing the counts of disconnected novel links versus other disconnected protein pairs was constructed and tested for independence using Fisher’s exact test.

### Analysis of Interaction Interfaces in Parents

Structures of proteins in complex with proteins, DNA, or RNA molecules were obtained from the PDB and PISA database (http://www.ebi.ac.uk/pdbe/pisa/). Interface residues were identified and their positions converted into Ensembl protein coordinates. PISA residue densities were calculated by counting unique positions and dividing by protein lengths. Differences in the distributions of interface-forming PISA residue densities were analyzed as before. Biological process and protein class enrichments for parent genes that donate ten or more interface-forming residues to fusion proteins were calculated.

### Analysis of Short Linear Peptide Motifs in Parents

A set of 1,410 experimentally validated (Dinkel et al., 2014) and 1,036,282 computationally predicted (Dosztányi et al., 2009) LMs were acquired and mapped onto proteins. LM densities were calculated by counting unique ELM accessions and dividing by protein length. Differences in LM density were assessed across parent gene sets and across included versus excluded segments. Due to the small sample size of experimentally verified LMs, functional enrichments were reported even if the number of genes in an enriched category was less than ten. Parent proteins that donate LMs to fusion proteins were assessed for functional enrichments.

### Analysis of PTM Sites

PTM sites, which are candidate sites for regulating protein interactions, were acquired from the PTMcode v2 data set (Minguez et al., 2015). Differences in PTMcode site densities per gene were assessed for different parent gene sets and across included versus excluded segments. Further, we obtained and cleaned a data set of experimentally validated PTMs (dbPTM 3.0 database; Lu et al., 2013). PTM densities were analyzed as before at the whole protein and fusion segment level. Enrichments of specific types of modification sites were quantified in included and excluded segments.

### Analysis of TF Fusions and the Expression Levels of Target Genes

Fusion transcripts in TCGA samples (Yoshihara et al., 2015) were filtered to identify fusions involving TFs (n = 1,131) (Table S7). The TCGA database (Tomczak et al., 2015) was queried to identify matched RNaseq data for TF fusion containing samples (n = 29). Normalized expression counts for each matched sample pair were extracted, genes with extremely small read counts (n < 10) removed, and DGE calculated as the absolute log_2_ fold change between the diseased and healthy samples. The regulated target genes of TFs were acquired from the TRRUST database (Han et al., 2015). DGE values for the TF targets were compared against all other genes using non-parametric Wilcoxon rank-sum tests in cases where sufficient regulatory targets (n ≥ 20) were available (n = 8). The resulting p values were corrected for multiple testing using Holm’s procedure.

## Author Contributions

Study Conception and Design: N.S.L., R.J.W., and M.M.B.; Acquisition of Data: N.S.L., L.M., M.E.O., J.G., and R.J.W.; Analysis and Interpretation of Data: N.S.L., L.M., M.B., R.J.W., T.F., and M.M.B.; Manuscript Writing: N.S.L. and M.M.B.; and Critical Inputs to Manuscript: N.S.L., L.M., T.F., M.E.O., R.J.W., M.B., and M.M.B. The project was led by N.S.L. and supervised by M.M.B.

## Figures and Tables

**Table 1:** OGs Losing ≥5 UB Sites and Tumor Suppressor Genes Gaining ≥5 UB Sites as a Result of Fusion Events

Fusion Accession^a^	OG	Description	Number of UB Sites Lost	Length of OG Retained Segment	Fusion Partner	Description of Fusion Partner
BF736842	EGFR	epidermal growth factor receptor	17	25	SLC12A9	solute carrier family 12, member 9
AK098472	CTNNB1	catenin (cadherin-associated protein), beta 1, and 88 kDa	9	420	RP11-345J4.5	bolA-like protein 2
BE176861	COPS5	COP9 signalosome subunit 5	9	112	HNRNPH3	heterogeneous nuclear ribonucleoprotein H3 (2H9)
BE176782	COPS5	COP9 signalosome subunit 5	9	112	HNRNPH3	heterogeneous nuclear ribonucleoprotein H3 (2H9)
BG953255	CTTN	cortactin	9	21	MYC	v-myc avian myelocytomatosis viral OG homolog
BP430745	CSE1L	CSE1 chromosome segregation 1-like (yeast)	7	41	UGP2	UDP-glucose pyrophosphorylase 2
CN278368	TRIM32	tripartite motif-containing protein 32	7	36	DDX21	DEAD (Asp-Glu-Ala-Asp) box helicase 21
CV340327	ERBB2	v-erb-b2 avian erythroblastic leukemia viral OG homolog 2	6	21	NOMO1	NODAL modulator 1
BE273347	DCUN1D1	DCN1, defective in cullin neddylation 1, and domain containing 1	6	24	QTRT1	queuine tRNA-ribosyltransferase 1
BC001010	CDK4	cyclin-dependent kinase 4	6	30	RPL4	ribosomal protein L4
AW371253	ERBB2	v-erb-b2 avian erythroblastic leukemia viral OG homolog 2	5	49	RABGAP1	RAB GTPase activating protein 1
U08818	MET	met proto-OG	5	380	MIR548F1	microRNA 548f-1
U19348	MET	met proto-OG	5	380	MIR548F1	microRNA 548f-1
DA624159	TFG	TRK-fused gene	5	90	GPR128	G protein-coupled receptor 128

**Fusion Accession**	**Tumor Suppressor Gene**	**Description**	**Number of UB Sites Gained**	**Length of TSG Retained Segment**	**Fusion Partner**	**Description of Fusion Partner**

CD368725	TGFB1	transforming growth factor, beta 1	13	45	ATP50	ATP synthase, H^+^ transporting, mitochondrial F1 Complex, and O subunit
DB041801	SMARCA4	SWI/SNF related, matrix associated, actin dependent regulator of chromatin, subfamily a, and member 4	9	78	UBB	ubiquitin B
BP213958	ARID1A	AT rich interactive domain 1A (SWI-like)	6	34	DNAJA2	DnaJ (Hsp40) homolog, subfamily A, and member 2
DB120764	EEF1A1	eukaryotic translation elongation factor 1 alpha 1	6	4	HIST1H2AM	histone cluster 1, H2am
BG035867	EIF1	eukaryotic translation initiation factor 1	6	38	RALY	RALY heterogeneous nuclear ribonucleoprotein
AB209020	GJA1	gap junction protein, alpha 1, and 43 kDa	6	136	IFT140	intraflagellar transport 140
BG926120	PDCD4	programmed cell death 4 (neoplastic transformation inhibitor)	5	110	GAPDH	glyceraldehyde-3-phosphate dehydrogenase
BC001412	EEF1A1	eukaryotic translation elongation factor 1 alpha 1	5	462	LASP1	LIM and SH3 protein 1
BQ962146	E2F1	E2F TF 1	5	8	RDH11	retinol dehydrogenase 11 (all-*trans*/9-*cis*/11-*cis*)
CK004088	NDRG2	NDRG family member 2	5	153	RPL38	ribosomal protein L38

a ChiTaRS fusion event accessions are listed along with affected genes, retained segment lengths, and tallies of UB site gain or loss.

## Supplemental Information

Document S1. Supplemental Experimental Procedures and Figures S1–S7

Table S1. Fusion Proteins and Parent Functions, Related to Figure 1 The table shows a description of fusion proteins used in this study and biological process and protein class enrichments of parent genes. The gene symbols in the original ChiTaRS mapping can differ from gene names associated with mapped Ensembl proteins (see Supplemental Information), and we provide both gene name sets in the fusion protein listing (see the fusion protein mapping web server http://www.fusion.d2p2.pro/ for further details).

Table S2. Functions of the Top Quartile Centrality Genes, Related to Figure 2 The table shows the biological process and protein class enrichments of parent and non-parent genes with the highest PPI network centralities.

Table S3. Tissue-Specific Network Centrality, Related to Figure 2 The table shows the averaged network centrality measures for parent and non-parent proteins in tissue-specific interaction networks.

Table S4. Interaction-Mediating Domains in Fusion Proteins and Parent Functions, Related to Figure 3 The table shows IMD residues incorporated into fusion proteins and biological process and protein class enrichments of parent genes that donate ≥20% of an IMD.

Table S5. Retained and Novel PPI Arising from Fusion-Mediated Domain Recombination, Related to Figure 4 The table shows the retained and novel PPIs resulting from the transfer of largely intact (≥90% of the domain sequence) IMD into fusion proteins.

Table S6. Interfaces, Linear Motifs, and PTMs in Fusion Proteins and Parent Functions, Related to Figure 3 The table shows structural interfaces of protein complexes that are incorporated into fusion proteins and biological process and protein class enrichments of parent genes that donate ten or more interface forming residues; experimentally validated short linear peptide motifs incorporated into fusion proteins and biological process and protein class enrichments of parent genes that donate at least one such linear motif; and experimentally validated PTMs incorporated into fusion proteins. Also, biological process and protein class enrichments for parent genes that either retain or lose ≥90% of their PTM content upon fusion. Certain PTM types were found to occur in both included and excluded segments more frequently than expected given the global frequencies of all known PTMs, such as S-Nitrosylation (1.7× enrichment in included segments and 1.6× in excluded segments). Other PTM types showed differential presence/absence patterns based on segment inclusion: methylation sites are more highly enriched in included segments (3.5× enrichment) than in excluded segments (2.5×), as are acetylation sites (1.8× included and 1.3× excluded). Interestingly, both N-linked and O-linked glycosylation, which are involved in protein folding and stability and cancer processes like migration and invasion, are generally depleted in parent proteins.

Table S7. Fusion-Mediated Deregulation of TF Target Genes, Related to Figure 6 The table shows a differential expression analysis of breast cancer samples containing fusion transcripts composed of at least one TF parent. The differential gene expression values of the TF targets were compared to those of all other genes.

Document S2. Article plus Supplemental Information
