# Supplementary material for: Molecular Principles of Gene Fusion Mediated Rewiring of Protein Interaction Networks in Cancer
Source: Mol Cell. 2016 Aug 18;63(4):579–92. doi: 10.1016/j.molcel.2016.07.008 (PMC5003813; doi:10.1016/j.molcel.2016.07.008)
Supplement: Document S1. Supplemental Experimental Procedures and Figures S1–S7 [file mmc1.pdf]

Molecular Cell, Volume *63*

Supplemental Information

Molecular Principles of Gene Fusion Mediated Rewiring of Protein Interaction Networks in Cancer

Natasha S. Latysheva, Matt E. Oates, Louis Maddox, Tilman Flock, Julian Gough, Marija Buljan, Robert J. Weatheritt, and M. Madan Babu

Latysheva, Fig. S1 (related to Fig. 1)

A Gene fusion network

(zoomable svg format)

Nodes = genes
Edges = presence of a gene fusion between the two genes

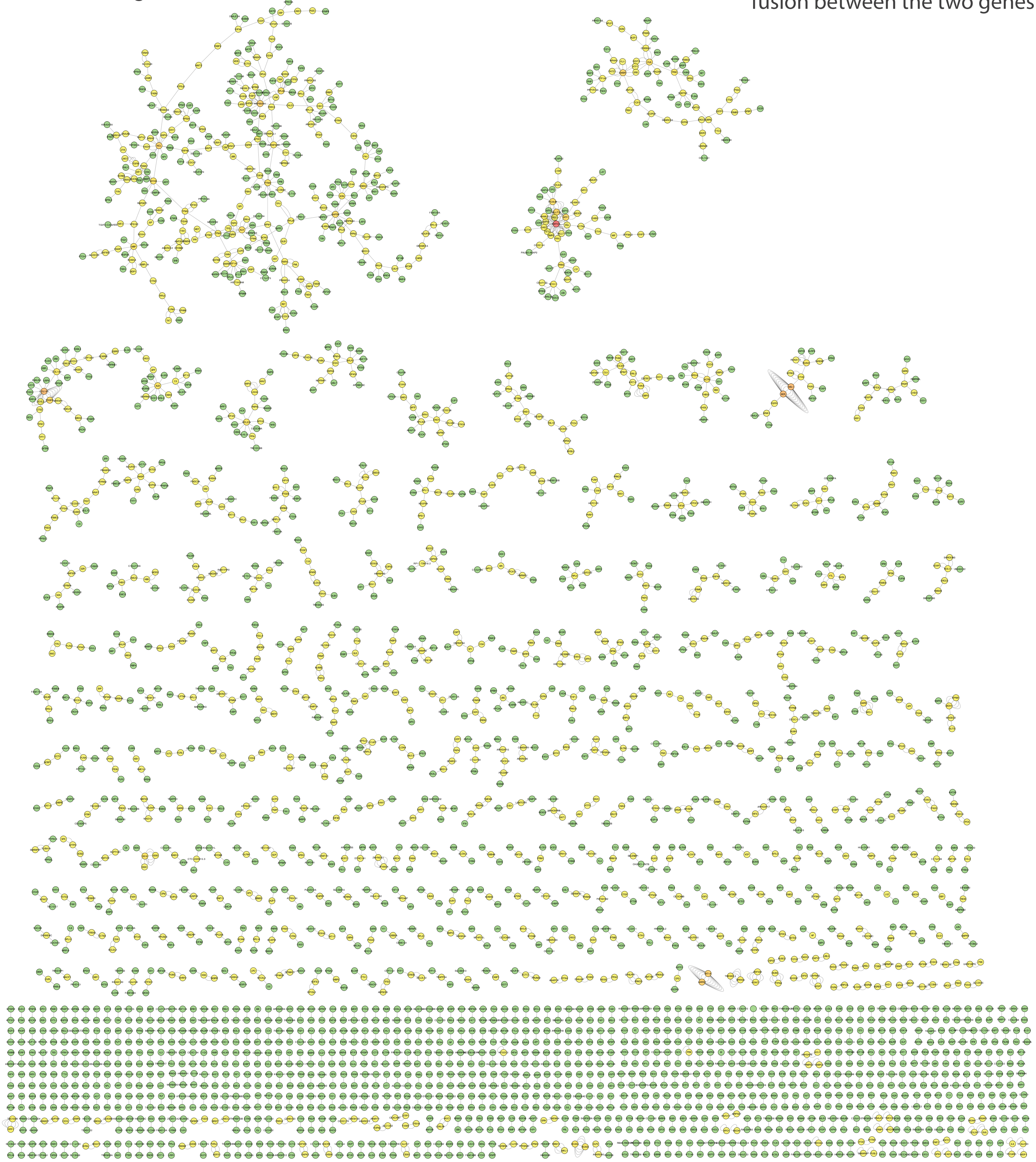

B Distribution of degree

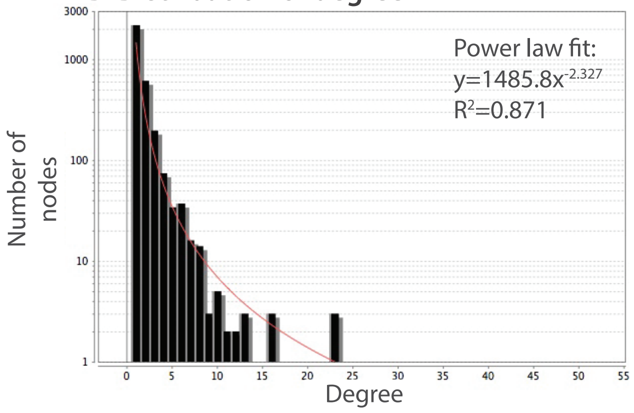

Node colour:

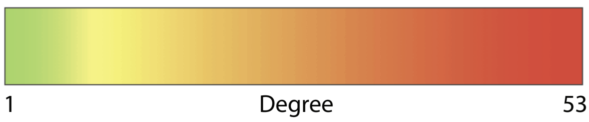

Figure S1. Network of gene fusions (related to Figure 1). (A) Nodes indicate genes and edges indicate the occurrence of a fusion between genes. Node colour indicates the degree of each node, or the count of distinct genes the node/gene fuses with. **(B)** Distribution of degree in the gene fusion network with a fitted power law curve. Note that one instance of fusion degree greater than 23 exists (KTM2A degree=53) but is excluded from the degree chart due to logarithmic scales. Oncogenes (OGs) and tumour suppressor genes (TSGs) are overrepresented in the parent set (OG: χ^2=62.1, df=1, p=3.3e^{-15}; TSG: χ^2=42.1, df=1, p=8.5e^{-11}). Over a third of known OGs (78/216) and a quarter of known TSGs (161/626) form gene fusions ($n_{OG\ fusions}$=184; $n_{TSG\ fusions}$=291) within this dataset. There exist instances of OG-TSG (n=38), OG-OG (n=14) and TSG-TSG (n=11) fusions.

Latysheva, Fig. S2 (related to Fig. 1)

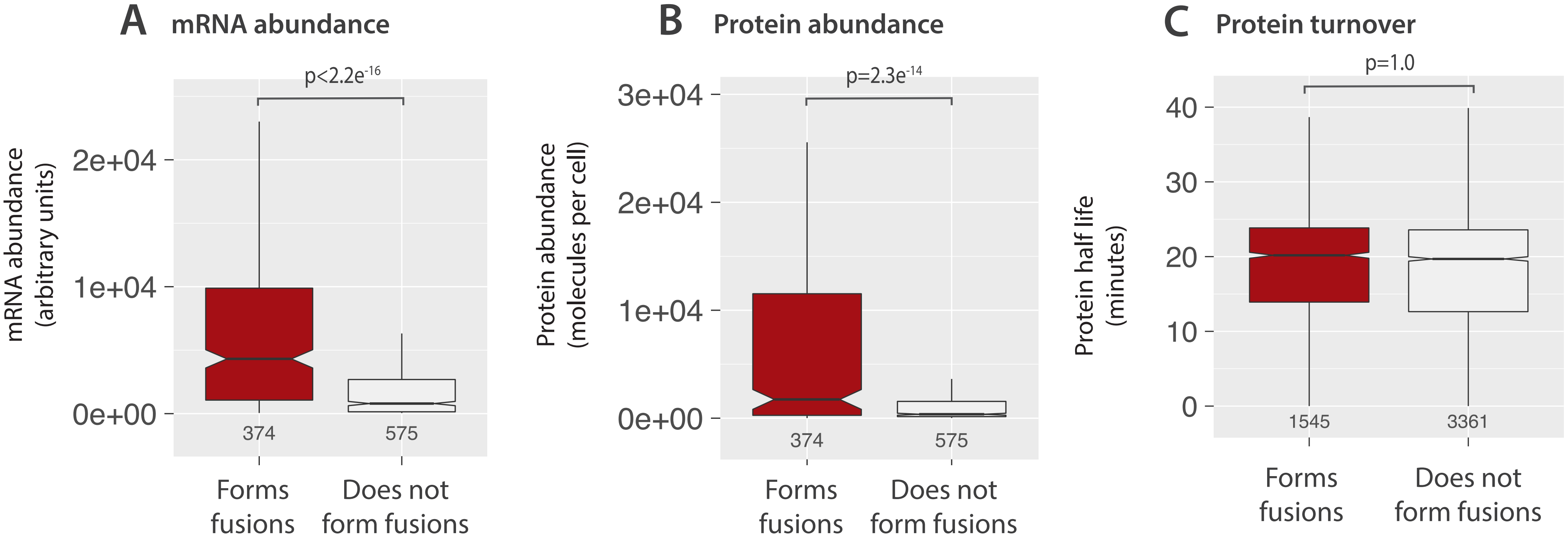

D Oncogenic signaling genes

	Oncogenic signaling gene	Not oncogenic signaling gene
Parent gene	57	3281
Non-parent gene	124	16941

χ^2=29.5, df=1, p=5.7e-08

E Essentiality of parent genes

	Essential gene	Not essential gene
Parent gene	576	2762
Non-parent gene	1151	15914

χ^2=396.8, df=1, p<2.2e-16

Figure S2. mRNA abundance, protein abundance, protein half-lives and gene identities in parents (related to Figure 1). (A) mRNA and **(B)** protein abundances of parent and non-parent genes from the Daoy medulloblastoma cell line. **(C)** Protein half-lives of parent and non-parent genes from SILAC experiments in HeLa cells. Enrichment of parent genes for **(D)** cancer signaling genes and **(E)** genes essential for cellular viability.

Latysheva, Fig. S3 (related to Fig. 2)

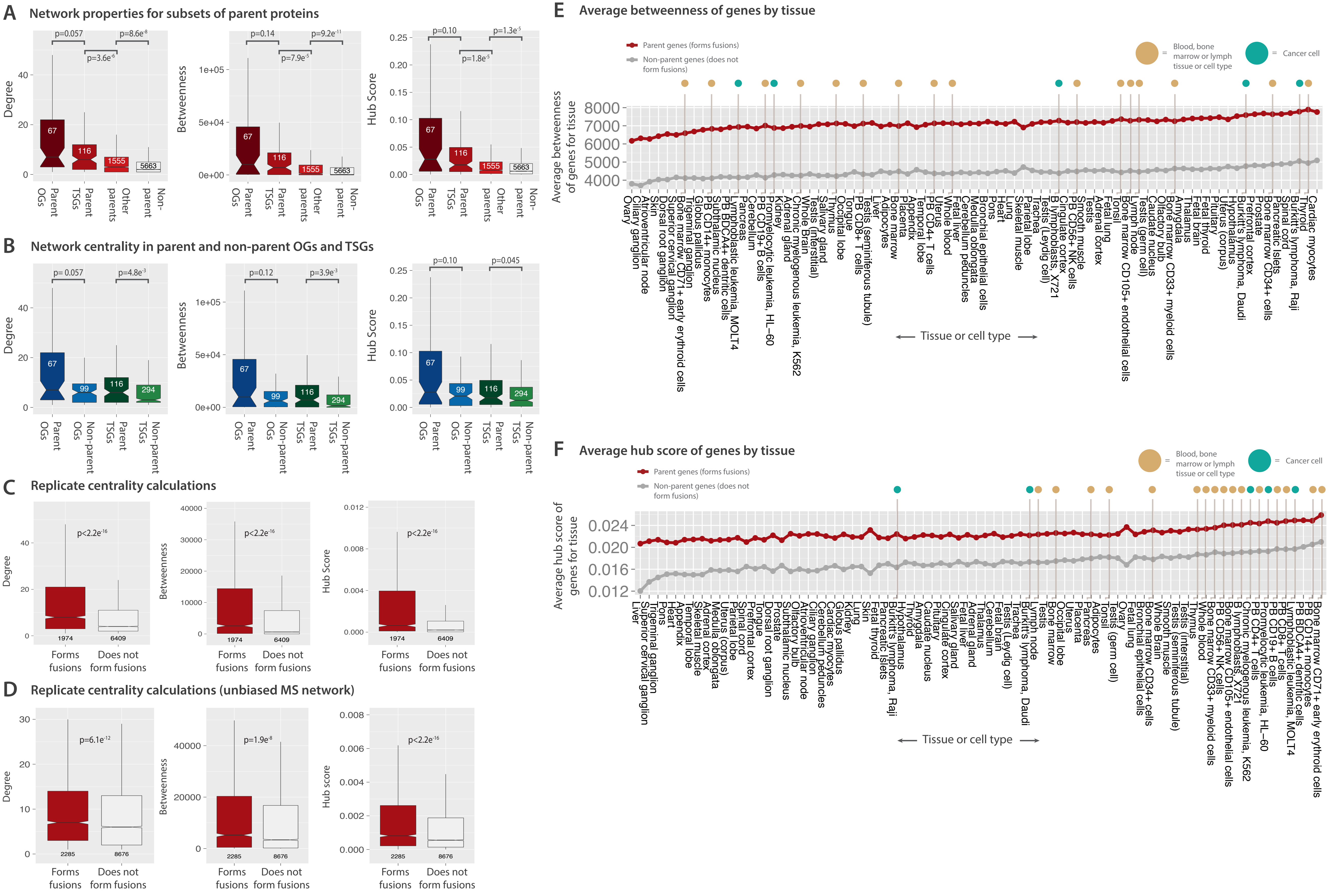

Figure S3. Network centrality support calculations (related to Figure 2). (A) Network centrality scores for parent gene sets and non-parents. **(B)** Network centrality scores for parent and non-parent oncogenes and tumour suppressor genes. **(C)** Replicate network centrality calculations for parent and non-parent genes on an additional PPI dataset. **(D)** Replicate network centrality calculations for parent and non-parent genes on an unbiased PPI dataset based on mass spectrometry data. Average tissue-specific betweenness centrality **(E)** and hub score centrality **(F)** of parent genes.

Latysheva, Fig. S4 (related to Fig. 2)

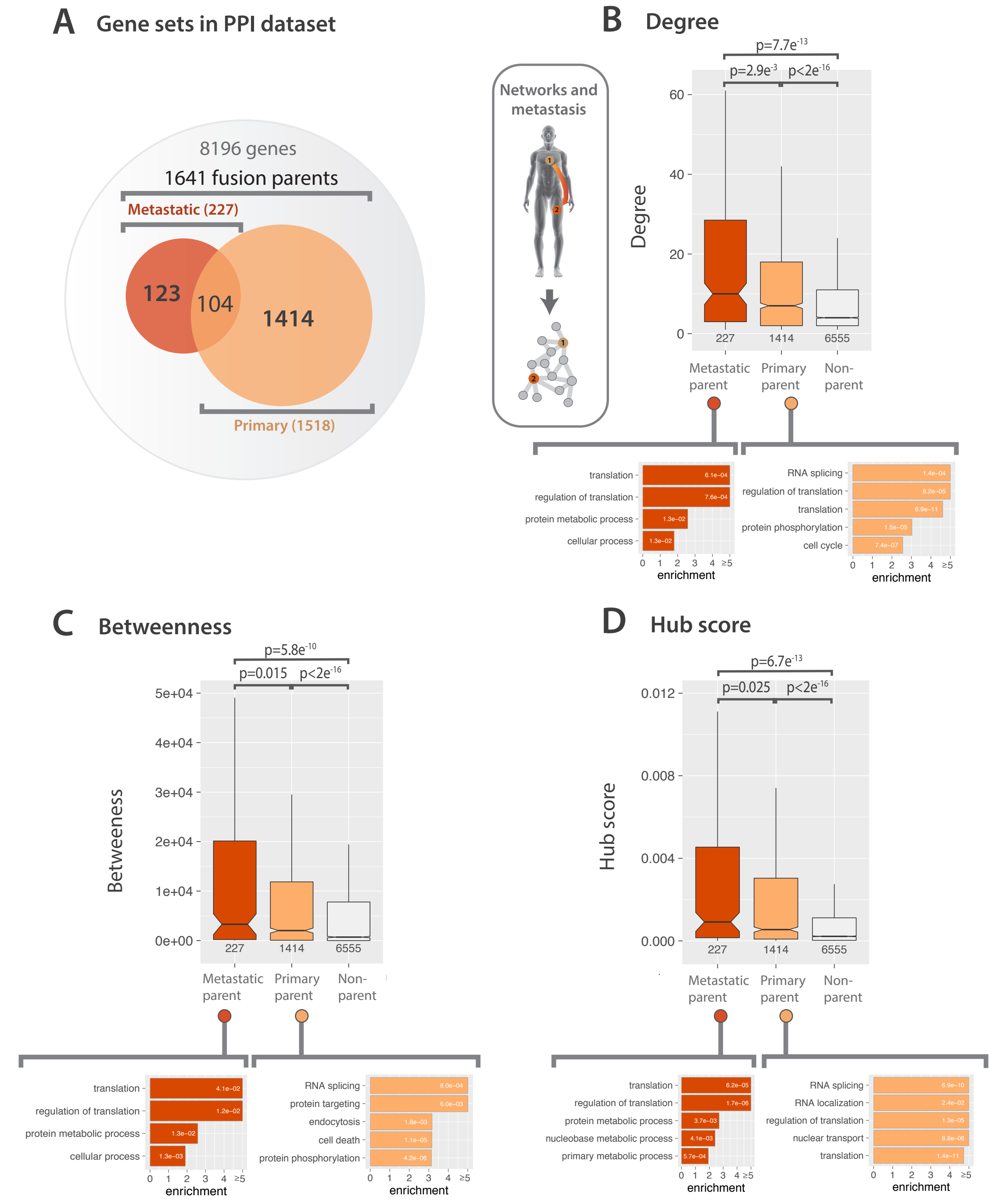

Figure S4. Network centrality in metastatic and primary tumour parent genes (related to Figure 2). Parent genes were labeled as “metastatic” if the gene formed a fusion that was detected in at least 1 cell line of metastatic tumour origin and “primary” if the gene only formed fusions detected in cell lines derived from primary tumours. **(A)** Counts of metastatic tumour parent genes, primary tumour parent genes, and non-parent genes within the PPI dataset. The degree **(B)**, betweenness centrality **(C)** and hub score **(D)** distributions by gene class. Bar plots show gene ontology enrichments in metastatic fusion genes in the top quartile of centrality values of the class. Although we find that parent genes from metastatic tumours have borderline significantly higher degree, betweenness and hub scores compared to parent genes from primary tumours, an alternative PPI dataset derived from MS data (Huttlin et al., 2015) does not replicate this trend (data not shown). There is an indication that the top quartile of metastatic and primary parents may have distinct functions (see also **Table S4**) - however, terms classically associated with metastasis (e.g. motility, tissue invasion, anoikis) were not found to be enriched.

Latysheva, Fig. S5 (related to Fig. 3)

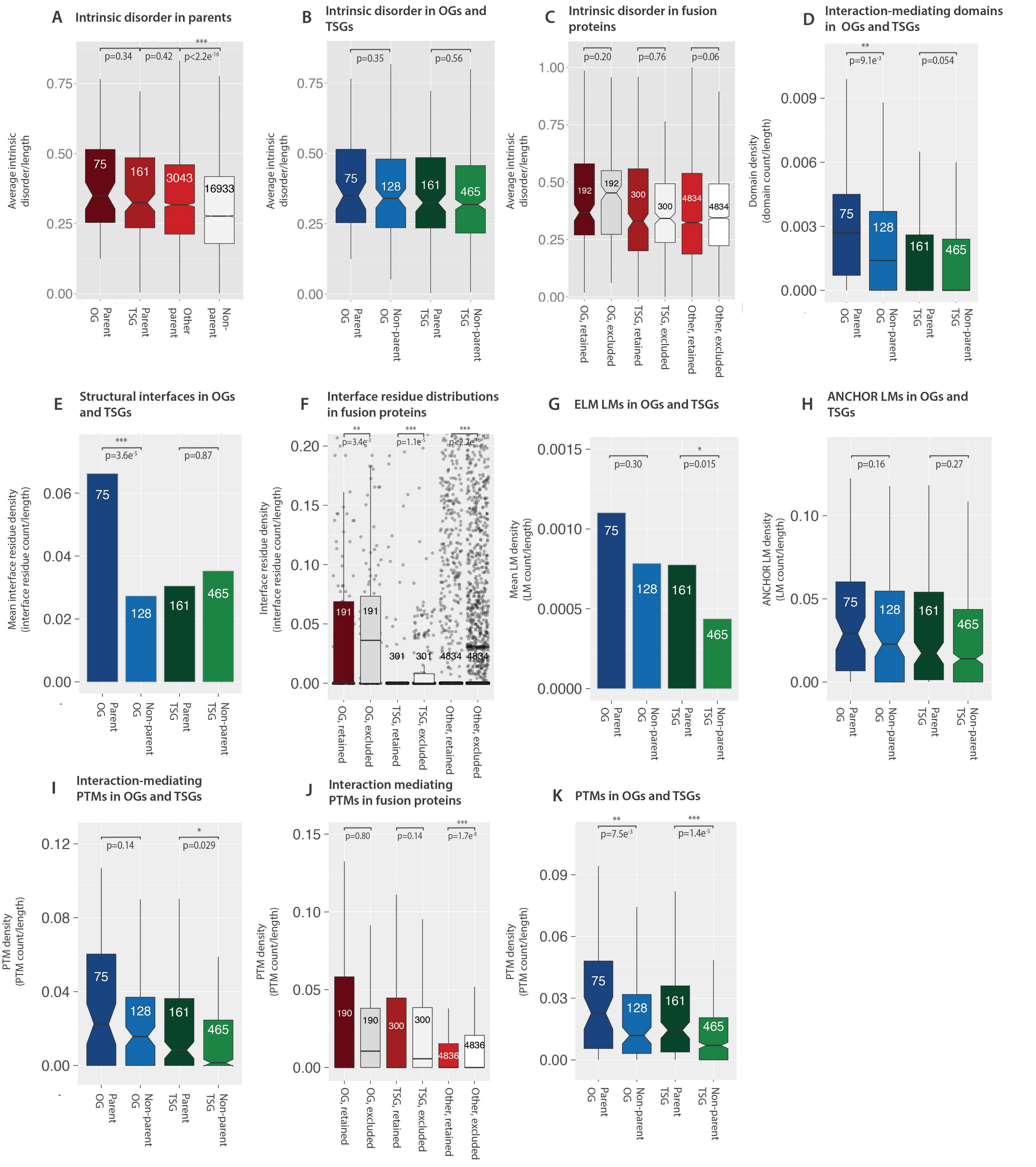

Figure S5. Molecular features of fusion proteins (related to Figure 3). (A) Average intrinsic disorder by gene for OG parents, TSG parents, other parents and non-parents. **(B)** Average intrinsic disorder in parent OGs and TSGs versus non-parent OGs and TSGs. **(C)** Average intrinsic disorder in included and excluded fusion segments by gene category. **(D)** Densities of interaction-mediating domains in parent versus non-parent OGs and TSGs. **(E)** Structural interface residue densities in parent versus non-parent OGs and TSGs. **(F)** Structural interface residue densities in included and excluded fusion segments by gene category. Plot data is identical to Figure 3D but with detailed distributions displayed instead of means. (**G**) Mean ELM linear motif densities in parent versus non-parent OGs and TSGs. (**H**) ANCHOR linear motif densities in parent versus non-parent OGs and TSGs. **(I)** Interaction-mediating PTM densities in parent versus non-parent OGs and TSGs. **(J)** Interaction-mediating PTM densities in included and excluded fusion segments by gene category. **(K)** PTM densities in parent versus non-parent OGs and TSGs.

Latysheva, Fig. S6 (related to Fig. 4)

A Retained domain-domain interactions in fusion proteins resulting from domain transfer

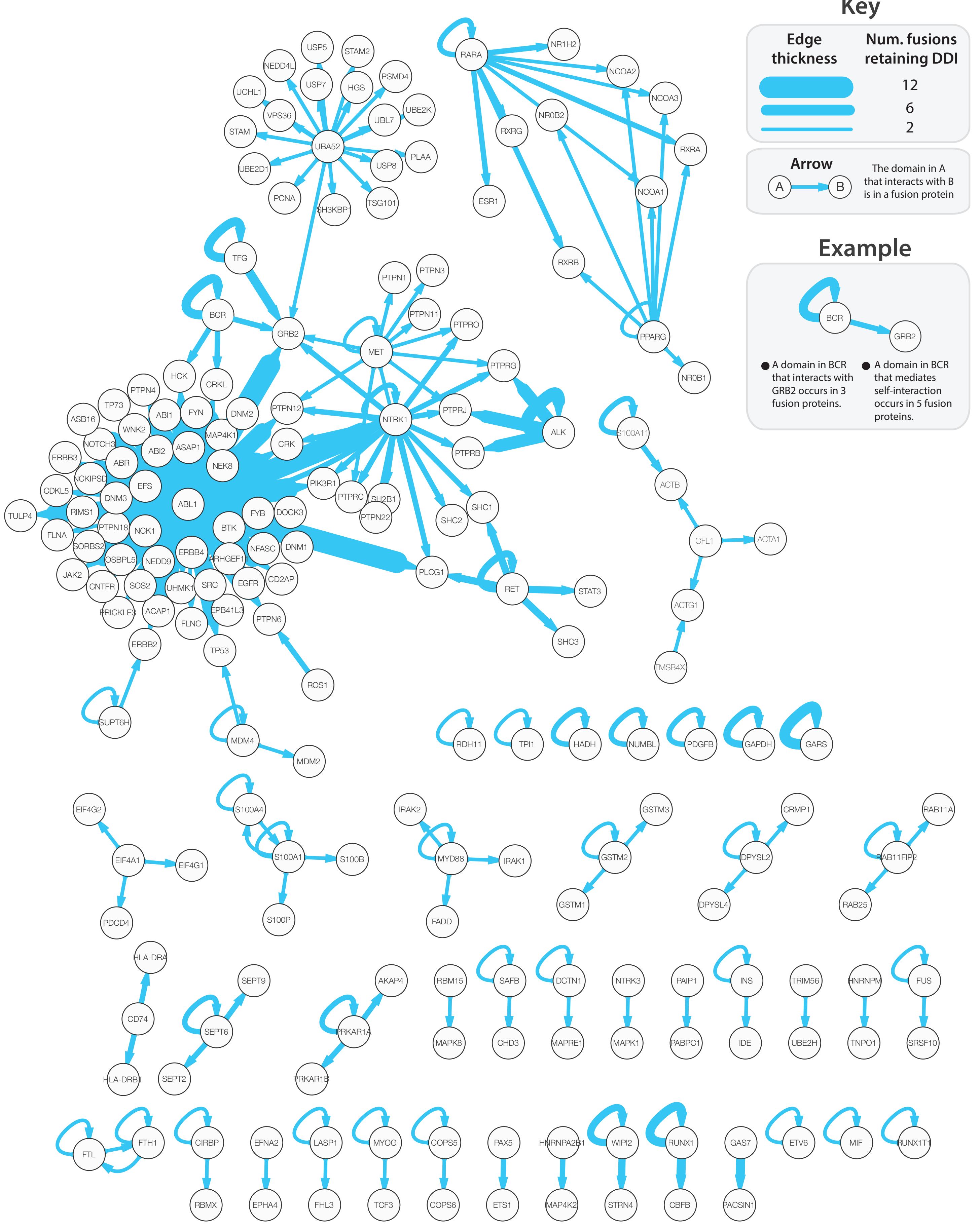

B Novel protein-protein interactions resulting from domain transfer

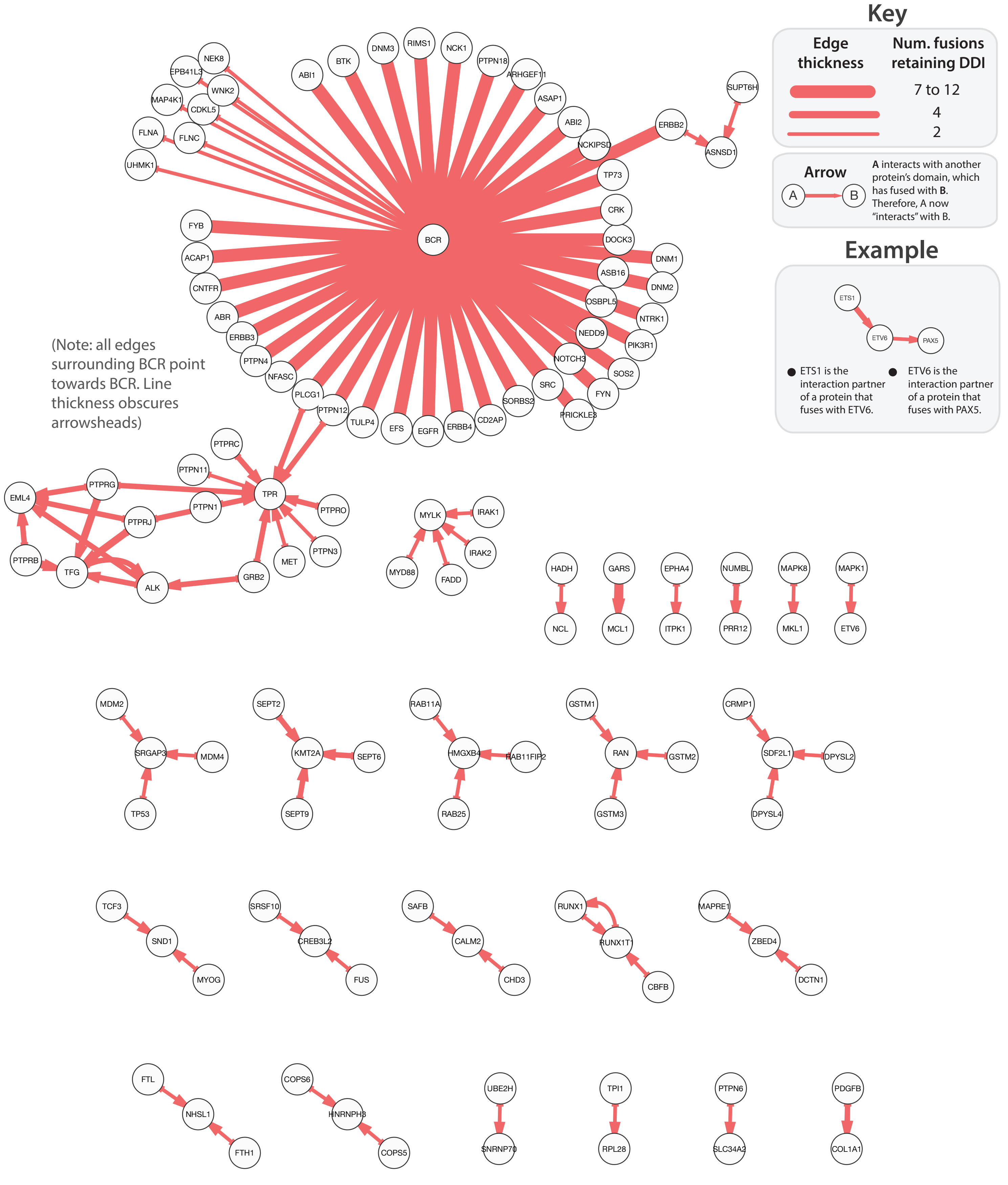

C Shortest path length distributions

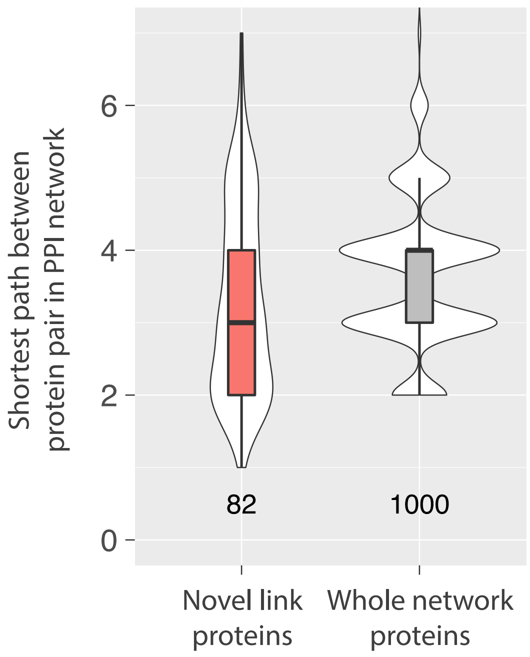

D Disconnected protein pairs

	No connecting path exists	Connecting path exists
Novel link protein pairs	34	82
All other protein pairs	5695274	47638419

Fisher's exact test, odds ratio=3.468, p=2.96e-08

E Protein pairs with no previous connecting path which are linked by fusion

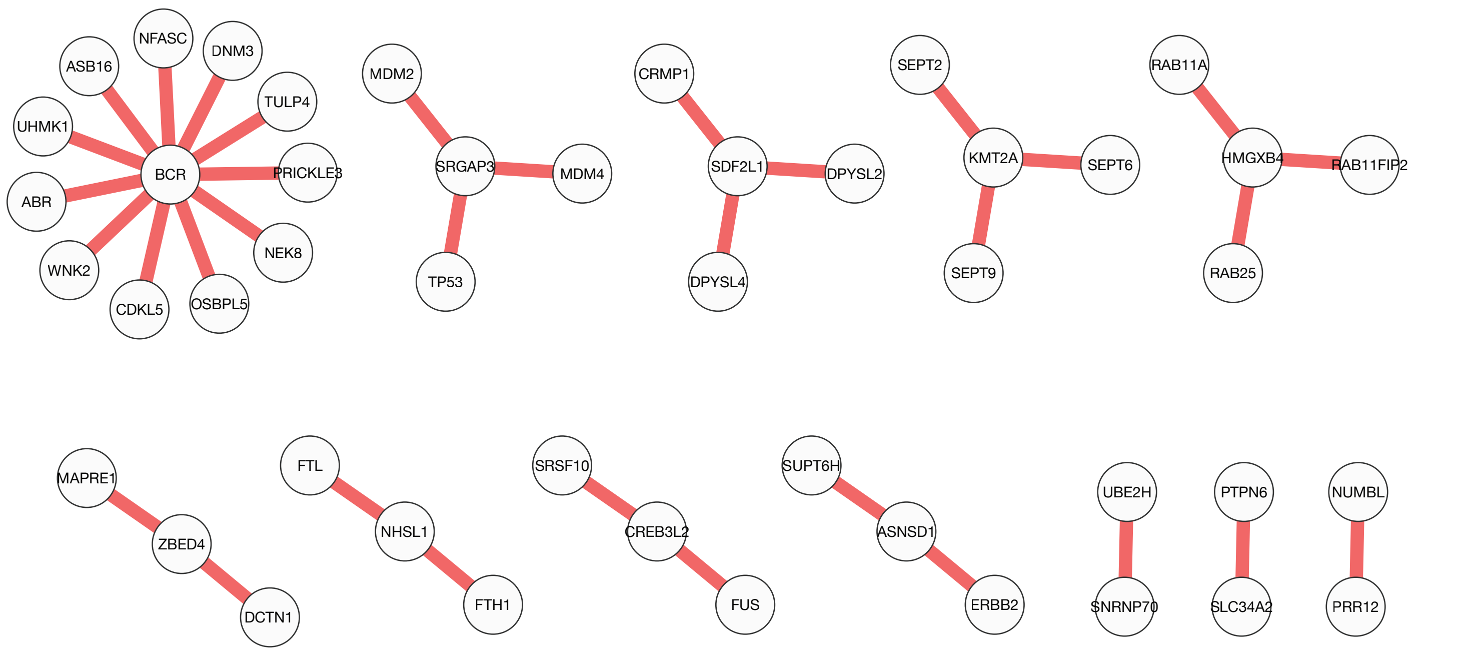

Figure S6. Retained and novel protein-protein interactions caused by fusion events (related to Figure 4). **(A)** Protein-protein interactions which are recurrently retained within fusion proteins as a result of the repeated inclusion of large portions (≥90% of residues) of interaction-mediating domains (IMDs). The most frequently conserved domain-domain interactions in fusion proteins include: the numerous interactions of ABL1, ALK interactions with ALK and three receptor-type protein tyrosine phosphatases (PTPRB, PTPRG, PTPRJ); GARS-GARS interaction; BCR interactions with BCR, CRKL, GRB2, HCK; and several RET interactions. The conserved domain-domain interactions can repeat - in certain cases, several different fusions conserve the same domain-domain interactions. For example, fusion proteins incorporating NTRK1, MET, RET, ALK, and BCR recurrently retain domains which mediate similar interactions. **(B)** Novel protein-protein links which arise from the recurrent transfer of large portions (≥90% of residues) of interaction-mediating domains (IMDs). A protein interaction link was drawn between proteins A and B if there existed some fusion protein B-C, where C normally interacts with A and at least 90% of C's interaction mediating domain was retained. Previously known PPIs were filtered out. We use "link" instead of "interaction" since these proteins do not necessarily interact – they are simply brought into proximity of each other, which may or may not result in interaction. **(C)** Fusion-mediated network rewiring of disparate areas of the native protein interaction network. Comparison of the shortest path distributions between proteins newly linked by fusion events and a set of 1000 random protein pairs from the interaction network. **(D)** Proportions of protein pairs with no available shortest path in the novel link set and in the whole network. The shortest paths between novel links were in fact slightly shorter than in other protein pairs in the network (on average, 3.28 compared to 3.71, respectively; W=32444, $p=1.0e^{-3}$). However, whereas only 10.7% of protein pairs in the PPI network had no connecting path, 29.3% of protein pairs in the novel links had no connecting path, indicating that the novel link set of protein pairs is disproportionately composed of protein pairs which previously had no existing path between them within the normal network. This reflects a strong enrichment for fusion proteins to interconnect protein sets which previously resided in completely separate sections of the interactome (Fisher's exact test on contingency table, odds ratio=3.47, $p=3.0e^{-8}$). **(E)** Newly linked protein pairs with no previous shortest path between them. For these proteins, fusion changes the previously infinite shortest path distances to a distance of 1. KTM2A (MLL), fusions of which are associated with aggressive leukaemia, is now connected to three members of the septin family (SEPT2, SEPT6, SEPT9), which are cytoskeletal components implicated in cancer development. Further, SRGAP3, a Rho GTPase activating protein, is now brought into proximity with MDM2, MDM4, and TP53, which are crucial regulators of cancer development and progression. Interestingly, MDM2 and MDM4 are known negative regulators of p53. Furthermore, BCR gains 11 novel links, which include proteins with roles in protein degradation and cell cycle progression.

Latysheva, Fig. S7 (related to Fig. 6)

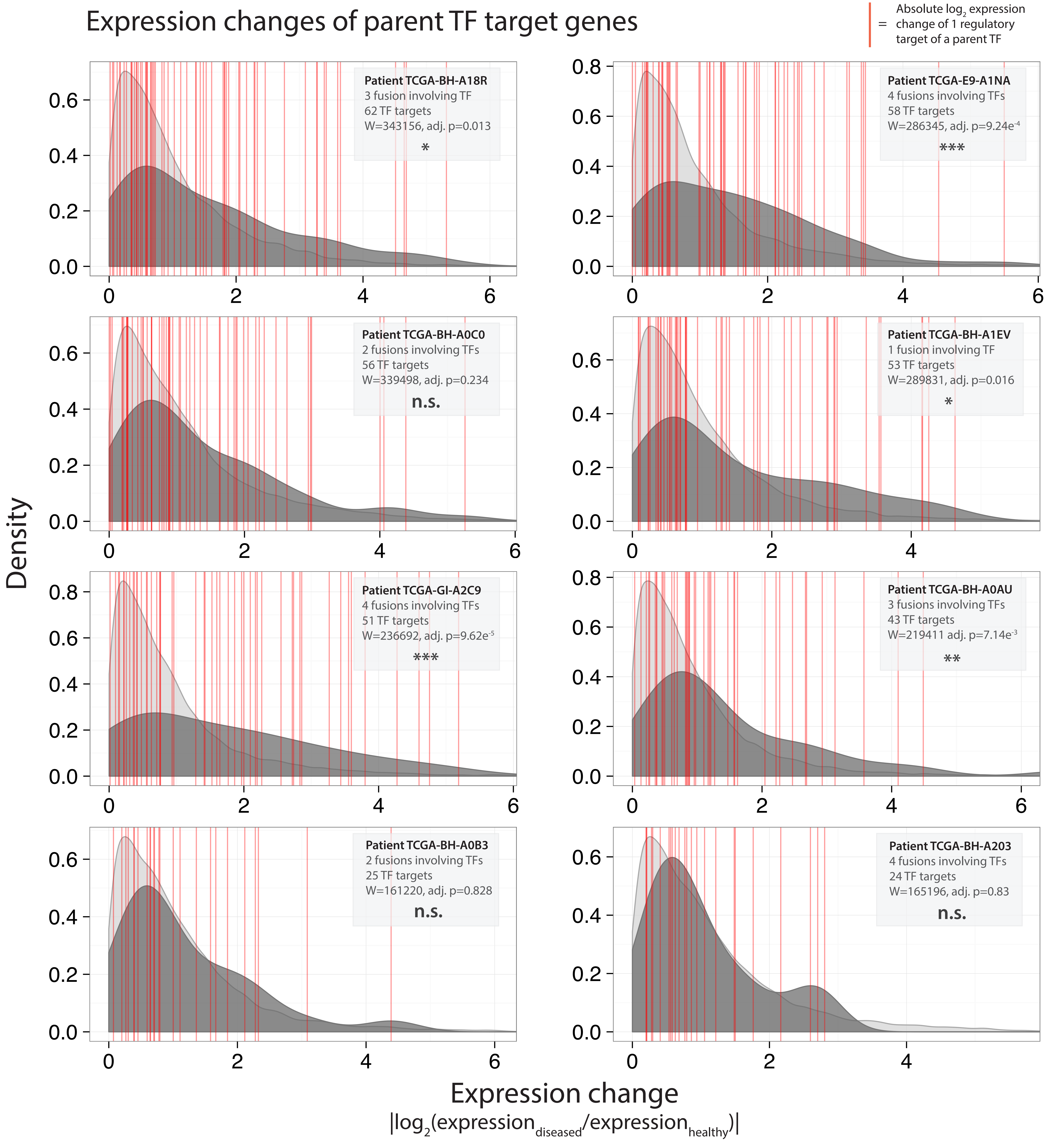

Figure S7. Fusion-mediated deregulation of transcription factor target genes in breast cancer (related to Figure 6). In fusions affecting transcription factor proteins, the downstream rewiring effects of the fusion may be investigated by analysing expression changes in the target genes of the transcription factor parent. Plots display comparisons of the differential gene expression (DGE) values of all genes (light grey distributions) in 8 breast cancer samples against DGE values of the regulatory targets (red vertical lines and dark grey distributions) of transcription factors forming the fusion transcripts within samples.

SUPPLEMENTAL TABLE LEGENDS

Table S1. Fusion proteins and parent functions (related to Figure 1). Description of fusion proteins used in this study and biological process and protein class enrichments of parent genes. Gene symbols in the original ChiTaRS mapping can differ from gene names associated with mapped Ensembl proteins (see **Supplemental Experimental Procedures**), and we provide both gene name sets in the fusion protein listing (see the fusion protein mapping web server http://fusion.d2p2.pro/ for further details).

Table S2. Functions of the top quartile centrality genes (related to Figure 2). Biological process and protein class enrichments of parent and non-parent genes with the highest PPI network centralities.

Table S3. Tissue specific network centrality (related to Figure 2). Averaged network centrality measures for parent and non-parent proteins in tissue-specific interaction networks.

Table S4. Interaction-mediating domains in fusion proteins and parent functions (related to Figure 3). Interaction-mediating domain (IMD) residues incorporated into fusion proteins and biological process and protein class enrichments of parent genes which donate ≥20% of an IMD.

Table S5. Retained and novel protein-protein interactions arising from fusion-mediated domain recombination (related to Figure 4). Retained and novel protein-protein interactions resulting from the transfer of largely intact (≥90% of the domain sequence) interaction-mediating domains into fusion proteins.

Table S6. Interfaces, linear motifs, and PTMs in fusion proteins and parent functions (related to Figure 3). Structural interfaces of protein complexes which are incorporated into fusion proteins and biological process and protein class enrichments of parent genes which donate 10 or more interface-forming residues; Experimentally validated short linear peptide motifs incorporated into fusion proteins and biological process and protein class enrichments of parent genes which donate at least one such linear motif; Experimentally validated post-translational modifications incorporated into fusion proteins. Biological process and protein class enrichments for parent genes which either retain or lose ≥90% of their PTM content upon fusion. Certain PTM types were found to occur in both included and excluded segments more frequently than expected given the global frequencies of all known PTMs, such as S-Nitrosylation (1.7x enrichment in included segments, 1.6x in excluded segments). Other PTM types showed differential presence/absence patterns based on segment inclusion: methylation sites are more highly enriched in included segments (3.5x enrichment) than in excluded segments (2.5x), as are acetylation sites (1.8x included, 1.3x excluded). Interestingly, both N-linked and O-linked glycosylation, which are involved in protein folding and stability and cancer processes like migration and invasion, are generally depleted in parent proteins.

Table S7. Fusion-mediated deregulation of transcription factor target genes (related to Figure 6). Differential expression analysis of breast cancer samples containing fusion transcripts composed of at least 1 transcription factor (TF) parent. Differential gene expression values of the TF targets were compared to those of all other genes.

SUPPLEMENTAL EXPERIMENTAL PROCEDURES

To compare properties of parent genes and proteins against non-parents, all human gene and proteins and their sequences were first acquired using Ensembl's ftp page (http://www.ensembl.org/info/data/ftp/index.html). Where possible, parent versus non-parent comparisons were performed at the gene level to avoid systematic biases for or against proteins with multiple isoforms. Unless otherwise specified, calculations of structural features for genes were obtained by considering the longest protein isoform. For network and structural analyses, parent genes were categorized according to whether they were known oncogenes or tumour suppressor genes. Throughout this study, the biological function of gene sets was assessed using statistical overrepresentation tests on PANTHER GO-Slim biological process and PANTHER protein class annotations, and unless stated otherwise, functional enrichments were only reported in the main text if 10 or more genes were present in a category.

For evaluating statistical significance of the differences in distributions between gene sets, non-parametric Wilcoxon rank sum tests were performed using R. In plots comparing more than two distributions against each other (e.g. Figure 3A, 3C), Holm's correction for multiple testing was employed. Following plotting convention, 1 star ('*') indicates $p \leq 0.05$, 2 stars ('**') indicates $p \leq 0.01$, and 3 stars ('***') indicates $p \leq 0.001$. All data integration and analysis was performed using custom R, Python and bash scripts and SQL queries. Visualization was done using the ggplot2 package in R, Cytoscape, and Adobe Illustrator. The webserver displaying fusion protein mappings (http://fusion.d2p2.pro) was generated via integration with the D^2P^2 resource (Oates et al. 2013).

Database identification, processing and integration

To compose a set of human fusion proteins, we acquired a database (ChiTaRS database v1; Frenkel-Morgenstern et al. 2013) of 9,237 fusion mRNAs, which represented the largest collection of human fusion sequences until the release of ChiTaRS v2. ChiTaRS is derived from a large scale analysis of EST and RNA-seq data from GenBank (Benson et al., 2012). GenBank obtains submissions primarily from individual laboratories and large-scale sequencing projects. The ChiTaRS project labeled entries as 'chimeric' if mRNA sequences, aligned to reference genomic sequences using UCSC BLAT program (Dreszer et al., 2012; Kent, 2002), mapped to two different genes at least 750 kb apart (maximum intron size for BLAT) and were less than 50 nt away from a splice site, since genuine fusions tend to have junctions proximal to splice sites (Hahn et al., 2004; Kim et al., 2010). Read-through fusions, which would be excluded by these criteria, were added at a later stage. ChiTaRS also contains fusion transcripts extracted from previous efforts to catalogue fusions, including the TICdb (Novo et al., 2007), dbCrid (Kong et al., 2011), ChimerDB 2.0 (Kim et al., 2010) and Mitelman (Mitelman et al., 2007) databases. Hence, ChiTaRS represents a comprehensive catalogue of fusion transcripts generated by a variety of mechanisms and detected in diverse studies, supported by manual inspection of research articles.

The fusion transcripts were mapped onto known protein sequences in the Ensembl database by translating ChiTaRS genomic alignment coordinates into corresponding protein coordinates. Instances in which genomic coordinates mapped to non-exonic regions such as intronic, UTR or intergenic sequences were discarded in order to isolate fusions affecting protein-coding regions. We further limited our analysis to fusion proteins in which both parents were mapped onto known Ensembl proteins. Full details of the fusion protein mapping are available at http://fusion.d2p2.pro. The gene symbols provided by the original ChiTaRS database for ChiTaRS genomic regions occasionally differ from gene symbols associated with mapped Ensembl proteins, often due to gene symbol aliasing, the use of cDNA alignment accessions instead of gene names by ChiTaRS, or ambiguity associated with the presence of overlapping UTRs and exons in the genome. We default to using gene symbols consistent with the Ensembl framework, and label gene names as ChiTaRS gene names where required. Original ChiTaRS v1 gene names for fusion events are available at http://chitars-old.bioinfo.cnio.es/, are provided along with Ensembl protein mappings at http://fusion.d2p2.pro, and both gene name sets are also presented as part of **Table S1**.

Oncogene and tumour suppressor gene datasets

A list of 239 human oncogenes was retrieved from the Tumour Associated Gene (TAG) database (Chen et al. 2013; from http://www.binfo.ncku.edu.tw/TAG/GeneFinder_chr.php), and 216 protein-coding oncogenes were successfully integrated into our fusion protein database. The TAG database identified both oncogenes and tumour suppressor genes by text-mining the PubMed database and collecting well-studied genes confidently implicated in oncogenesis. 716 human tumour suppressor genes (TSGs) were acquired from the TSGene web resource for tumour suppressor genes (Zhao et al., 2013; from http://bioinfo.mc.vanderbilt.edu/TSGene/) and of these, 626 protein-coding tumour suppressor genes were integrated. TSGene is a comprehensive literature-based knowledgebase of human, mouse and rat tumour suppressor genes presented with supporting functional and expression annotation. Structural feature density calculations were performed on parent proteins across gene sets (parent OG n=75; parent TSG n=161; other parent n=3043; non-parent n=16933; the 3 parent OGs which were also TSGs were classed as TSGs), as well as on included and excluded segments of parent proteins (OG segments n=198; TSG segments n=302; other parent segments n=4837). Minor differences in these sample sizes across certain calculations can result from constraints of overlapping multiple data sets (i.e. accurately mapping between different accession frameworks).

Parent protein mRNA and protein abundance and half-lives

Protein and mRNA abundances were acquired from (Vogel et al., 2010), which experimentally measured absolute protein and mRNA concentrations in the Daoy medulloblastoma cell line using shotgun proteomics and microarrays, respectively. The dataset covers 1,051 proteins, for which protein expression levels (molecules per cell) and mRNA expression levels (arbitrary units) are available. In the 86 cases where measurements were available for multiple isoforms of the same gene, the values were averaged to obtain gene-level measures. The identity of the 3,339 parent genes was overlapped onto the dataset, leading to 374 parents mapped to the abundance data. The remaining genes were regarded as non-parents. Statistical significances of the differences in abundance distributions were quantified using non-parametric Wilcoxon rank-sum tests.

Protein half-lives were acquired from a SILAC (stable isotopic atoms for quantitative mass spectrometry analysis) study in HeLa cells (Boisvert et al., 2012), which quantified protein abundance and turnover. The study identified and quantified 80,098 peptides and mapped these onto 8,041 endogenous HeLa cell proteins (yielding an average of ~10 peptides per protein). Protein turnover rates ranged from under 10 minutes to over a hundred hours, with an average turnover rate of ~20 h. 1,545 parent genes were mapped to the dataset, and the remainder was regarded as non-parent genes. Differences in half-life distributions were quantified as before.

Parent gene participation in oncogenic signaling blocks

To investigate if parent proteins are more likely than expected to participate in cancer signaling processes, 328 gene members of 12 oncogenic 'signaling blocks' were acquired. Signaling blocks were derived from an analysis that integrated a manually curated human signaling network with information on cancer-associated genetically and epigenetically altered genes (Cui et al., 2007). 181 of the provided 'gene names' were recognized and incorporated into the analysis. A contingency table of parent genes against signaling genes was constructed and tested using a chi-squared test of independence.

Protein-protein interaction network datasets

The Wang dataset of binary protein-protein interactions (Wang et al., 2012) consists of 12,500 literature-curated binary interactions taken from 6 databases and 8,000 well-verified high-throughput yeast two-hybrid interactions. In total, 20,614 binary interactions were acquired from the dataset, composed of 7,401 unique genes, of which 1,738 were parent genes. Network centrality measures were calculated (**Experimental Procedures**) for parent and non-parents. Furthermore, an unbiased network of 56,553 protein-protein interactions from 10,961 proteins, derived from affinity purity mass spectrometry experiments (Huttlin et al., 2015), was acquired for further validation of centrality trends (data available at http://wren.hms.harvard.edu/bioplex/).

The tissue specific set of protein-protein interactions (Bossi and Lehner, 2009) was defined by acquiring ~81,000 interactions from 21 sources, with each interaction supported by at least one piece of direct experimental evidence demonstrating physical association, and integrating gene expression data to infer tissue specificity. A given protein-protein interaction was labeled as being present in a tissue if the two genes leading to the proteins are co-expressed above a certain threshold in the cells of that tissue, indicating that they could potentially interact in those cells. The resulting dataset contains 78 tissue-specific PPI networks and 1 "consensus", non-tissue specific network. The consensus network was composed of 8,383 unique genes, of which 1,974 were parent genes and 6,409 non-parents. Network centrality measures were calculated and compared between: 1) parents and non-parents, 2) different categories of parents (OGs, TSGs, and other parents) and 3) parent and non-parent OGs and TSGs. Averaged centrality values for parents and non-parents were also calculated for each cell or tissue type.

Protein-protein interaction network centrality in parent proteins

We first performed network centrality calculations on a non-tissue specific protein-protein interaction network (Wang et al., 2012). Using the igraph R package (http://igraph.org), the (undirected) degree centrality, betweenness centrality (Gursoy et al., 2008) and Kleinberg's hub score (Kleinberg, 2000) were calculated for both parent and non-parent genes/proteins. The degree of each node i in an undirected graph is defined as the number of edges incident upon a node. In PPI networks, this gives the number of interacting partners for a given protein. The degree centrality $C_d(i)$ of a node i is simply its degree, given by

$$C_d(i) = deg(i) = |N(i)|$$

where $N(i)$ is the number of neighbours of node i. Nodes with especially high degree centrality are also called 'hubs'.

The betweenness centrality of a node reflects the number of shortest paths (geodesics) from all nodes to all others that pass through it. Nodes take on high values for betweenness centrality if they lie on a high proportion of paths connecting all other nodes in the graph. Formally, the C_b of a node i is given by:

$$C_b(i) = \sum_{j<k} g_{jk}(i) / g_{jk}$$

where g_{jk} is the number of geodesics connecting nodes j and k, and $g_{jk}(i)$ is the number of geodesics that the node i lies on. Proteins with high betweenness centrality function as connectors within interaction networks and are sometimes referred to as 'bottlenecks'.

Kleinberg's hub score was first developed to rank web searches and identify "authoritative" pages (which are linked to by many other pages) and "hub" pages (which themselves link to many authorities). We calculate the hub score for each protein in the network, which captures each protein's connectedness to high degree proteins. The hub scores of nodes can be defined recursively by referencing authority scores, or using linear algebra notation can be more concisely defined as the principal eigenvector of the matrix $A*A^T$, where A is the adjacency matrix of the graph.

Network centrality of parents detected in cell lines derived from metastatic and primary tumours
All gene fusions that were detected in a recent complete transcriptomic screen of 675 human cancer cell lines (Klijn et al., 2015) were acquired. The screening of fusion candidates procedure involved checking for multiple breakpoint-spanning reads, correcting for read number, confirming their absence in normal tissues, and checking for in-frame status (Klijn et al., 2015). The dataset comprises 2,371 gene fusions formed from 3,161 parent genes. 2,119 fusions had available cell line information, allowing the identification of 220 gene fusions detected in metastatic tumours and the remainder in primary tumours. We mapped 1,641 parent genes to the consensus, non-tissue specific PPI network (**Figure 3A**)(Bossi and Lehner, 2009) and compared centrality metrics between 'primary parent' genes, 'metastatic parent' genes and 'other' genes. Genes were allocated to the 'metastatic' category if the gene formed a fusion that was detected in at least one cell line of metastatic tumour origin and to the 'primary' category if detected only in cell lines of primary tumour origin. All other genes were classed as non-parent genes.

Intrinsic structural disorder in parent proteins
Residue by residue predictions for structural disorder in the human proteome were calculated using IUPred (Dosztányi et al., 2005; http://iupred.enzim.hu/) as in the **Experimental Procedures**. All sequences in the human proteome was downloaded from Ensembl, processed using custom Python scripts, and input into IUPred (using "long" for the length parameter) to generate disorder predictions. The IUPred algorithm is based on analyzing amino acid composition to estimate the likelihood of polypeptides forming stabilizing inter-residue contacts.

Interaction-mediating domain dataset
The INstruct database (Meyer et al., 2013) is composed of a curated protein interactome network annotated to the structural resolution of individual domains. The dataset encompasses 6,585 interactions in human. Binary interactions were curated from popular interaction databases and then filtered to meet strict quality conditions: the interaction must have at least two separate supporting publications and each of these publications must have a binary evidence code. These interactions were then used to reconstruct 3D interaction interfaces by using co-crystal structures in PDB using a validated homology-based interaction interface inference approach. Densities of interaction-mediating domain residues were analyzed as described in the **Experimental Procedures**. Interaction-mediating domain (IMD) retention was calculated using IMD residue densities instead of simply counting the presence of untruncated IMDs due to the functional potential of large portions of retained IMDs even if they are not 100% retained (cf. linear motifs, for which a requirement of full retention was imposed due to their short length).

Analysis of interaction interfaces in parents
Structures of proteins in complex with either proteins, DNA or RNA molecules were obtained from the PDB and PISA database (http://pdbe.org/pisa). Residues at the interface were identified by parsing

PISA XML files using custom bash and Python scripts. Coordinates from PDB structures were corrected and mapped onto UniProt sequences using the Structure Integration with Function, Taxonomy and Sequence (SIFTS) API (Velankar et al., 2013). A total of 173,199 unique interface-forming residues were extracted from 3,125 proteins (97% of interfaces are protein-protein interfaces, 2.2% DNA-protein interfaces, and 0.8% RNA-protein interfaces). PISA residue densities were calculated by counting unique positions and dividing by protein length. Only the unique interface residues were analysed to control for highly studied proteins having more known interactions or complexes. Differences in the distributions of interface-forming PISA residue densities, as well as PISA residue retention in fusion proteins, were analyzed as before. Biological process and protein class enrichments for parent genes which donate 10 or more interface-forming residues to fusion proteins were calculated using PantherDB.

Linear motif datasets

Using the ELM database of 1,410 experimentally validated, manually curated short linear motifs (LMs) in eukaryotes (Dinkel et al., 2014), we tested for enrichment of human LMs in parent proteins. The linear motifs subtypes are: proteolytic cleavage sites, general ligand binding sites, degradation motifs (regions that promote protein polyubiquitylation and proteasomal degradation), docking sites (motifs that recruit an enzyme to a protein region that is not the active site), sites for post-translational modification and sub-cellular targeting sites. We tested for enrichment of LMs in parent proteins compared to other genes, and calculated the retention vs. exclusion of LMs in fusion proteins (see **Experimental Procedures**). The larger set of putative linear motifs was produced using the ANCHOR predictive method (Dosztányi et al., 2009), which is based on estimating the likelihood of a residue being a part of a disordered binding region by combining information on estimated energies (using the same method as the IUPred algorithm) and the local structural environment. Linear motif densities were calculated by counting unique ELM accessions and dividing by protein length. Differences in linear motif densities were assessed across parent gene sets, and across included versus excluded segments. Due to small sample sizes, functional enrichments were reported even if the number of genes in an enriched category was less than 10. Parent proteins which donate ELMs to fusion proteins were assessed for specific functions as before. To expand the linear motif analysis, putative linear motifs in the human proteome were computationally predicted using the ANCHOR resource (Dosztányi et al., 2009), identifying 1,036,282 short protein-binding regions. All predicted motifs were 6 amino acids in length with consistent scores over 0.4 and disorder scores over 0.2, with cut-offs based on a survey of annotated motifs from ELM (Davey et al., 2012). Due to the lower likelihood that partial linear motifs would retain functionality (due to their short length), both ELM and ANCHOR linear motif densities were calculated as the count of unique, untruncated linear motifs per amino acid residue.

Post-translational modification datasets and analysis

The dbPTM (Lu et al., 2013; http://dbptm.mbc.nctu.edu.tw/) database integrates experimental PTMs obtained from eleven public resources (UniProtKB/Swiss-Prot, Phospho.ELM, PHOSIDA, etc.) as well as from manual curation of research articles. PTMcode v2 catalogues pairs of PTMs within or between interacting proteins which are known or predicted to be functionally associated. These associated PTM sites, which were identified using co-evolution and structural distance models, are good candidate sites for regulating protein interactions (Minguez et al., 2014; http://ptmcode.embl.de/) through either their positioning in protein interfaces (Beltrao et al., 2012) or potentially through allosteric mechanisms (Nussinov et al., 2013).

Enrichments of specific types of modification sites were quantified for included and excluded segments as follows: the background PTM type frequencies for all of dbPTM were calculated, as were the PTM type frequencies for included and excluded segments. For PTM types that occurred 50 or more times in dbPTM, included fusion segments, and excluded fusion segments, fold changes were calculated for both included and excluded segments by dividing by background dbPTM frequencies. Normalized fold changes (1 - fold changes) were plotted to visualize enrichments.

Ubiquitination site gain and loss was analysed in oncogenes and tumour suppressor genes. Experimentally validated ubiquitination (UB) sites were gathered by filtering dbPTM. UB sites were mapped onto fusion protein coordinates, and the number of UB sites lost in oncogene parents and the number of UB sites in segments partnering with TSG parents was tabulated and compared to all other parental segments.

Analysis of transcription factor fusions and the expression levels of target genes

A database of all fusion transcripts identified in TCGA samples (Yoshihara et al., 2015)(http://54.84.12.177/PanCanFusV2/) was filtered to identify all fusion events involving transcription factors (TFs), yielding 1131 TF fusions from 818 samples (**Table S10**). The cancer type acting as the largest source of these fusions (BRCA, breast cancer invasive carcinoma; 461 fusions in 297 samples) was used for further analysis. TCGA was queried to identify instances in which Illumina HiSeq 2000 RNAseqV2 data was available for both the fusion-containing BRCA sample and a matched, healthy solid tissue sample. For these 29 instances, the gene-wise Level 3 RSEM normalized expression counts (i.e. upper quartile normalized RSEM count estimates) for each diseased/healthy pair were extracted. Genes with extremely small read counts ($n<10$) were removed to exclude genes with very low expression values. The RNAseq data was merged for each patient-matched diseased and healthy sample, and differential gene expression (DGE) values were calculated as the absolute $\log_2$ fold change between the diseased and healthy samples. For each gene fusion involving a TF in each BRCA sample, the regulatory targets of the TFs were acquired from the TRRUST database (Han et al., 2015). The absolute $\log_2$ fold change metric was chosen in order to simultaneously examine evidence for either up- or down-regulation of TF targets; further, the regulatory link (i.e. either "activation" or "repression") between TFs and their targets was frequently unknown (Han et al., 2015). DGE values for the TF targets were compared against the corresponding values for all other genes using non-parametric Wilcoxon rank-sum tests in cases where sufficient regulatory targets ($n\geq20$) were available ($n=8$). The resulting p-values were corrected for multiple testing using Holm's procedure.

We note that TCGA does not (and possibly could not) provide data from cells with a clean genetic background. In addition to a specific fusion, tumour samples contain a variety of other mutations (often in addition to several fusions), and hence the "background" levels of differential expression are likely to be affected by myriad factors. Furthermore, gene expression levels are influenced by a variety of post-transcriptional regulatory mechanisms (e.g. transcript stability). Despite these caveats and within these limitations, we identify evidence of heightened differential expression of target genes of transcription factors involved in fusions in our dataset. This provides support for potential downstream rewiring effects due to the fusion event for some proteins (TFs) in our dataset.

SUPPLEMENTAL REFERENCES

Beltrao, P., Albanèse, V., Kenner, L.R., Swaney, D.L., Burlingame, A., Villén, J., Lim, W.A., Fraser, J.S., Frydman, J., and Krogan, N.J. (2012). Systematic functional prioritization of protein posttranslational modifications. Cell *150*, 413–425.

Benson, D.A., Karsch-Mizrachi, I., Clark, K., Lipman, D.J., Ostell, J., and Sayers, E.W. (2012). GenBank. Nucleic Acids Res. *40*, D48–D53.

Chen, J.-S., Hung, W.-S., Chan, H.-H., Tsai, S.-J., and Sun, H.S. (2013). In silico identification of oncogenic potential of fyn-related kinase in hepatocellular carcinoma. Bioinformatics *29*, 420–427.

Dreszer, T.R., Karolchik, D., Zweig, A.S., Hinrichs, A.S., Raney, B.J., Kuhn, R.M., Meyer, L.R., Wong, M., Sloan, C.A., Rosenbloom, K.R., et al. (2012). The UCSC Genome Browser database: extensions and updates 2011. Nucleic Acids Res. *40*, D918–D923.

Gursoy, A., Keskin, O., and Nussinov, R. (2008). Topological properties of protein interaction networks from a structural perspective. Biochem. Soc. Trans. *36*, 1398–1403.

Hahn, Y., Bera, T.K., Gehlhaus, K., Kirsch, I.R., Pastan, I.H., and Lee, B. (2004). Finding fusion genes resulting from chromosome rearrangement by analyzing the expressed sequence databases. Proc. Natl. Acad. Sci. U. S. A. *101*, 13257–13261.

Kent, W.J. (2002). BLAT--the BLAST-like alignment tool. Genome Res. *12*, 656–664.

Kim, P., Yoon, S., Kim, N., Lee, S., Ko, M., Lee, H., Kang, H., Kim, J., and Lee, S. (2010). ChimerDB 2.0--a knowledgebase for fusion genes updated. Nucleic Acids Res. *38*, D81–D85.

Kleinberg, J. (2000). Navigation in a small world. Nature *406*, 845.

Klijn, C., Durinck, S., Stawiski, E.W., Haverty, P.M., Jiang, Z., Liu, H., Degenhardt, J., Mayba, O.,

Gnad, F., Liu, J., et al. (2015). A comprehensive transcriptional portrait of human cancer cell lines. Nat. Biotechnol. *33*, 306–312.

Kong, F., Zhu, J., Wu, J., Peng, J., Wang, Y., Wang, Q., Fu, S., Yuan, L.-L., and Li, T. (2011). dbCRID: a database of chromosomal rearrangements in human diseases. Nucleic Acids Res. *39*, D895–D900.

Novo, F.J., de Mendíbil, I.O., and Vizmanos, J.L. (2007). TICdb: a collection of gene-mapped translocation breakpoints in cancer. BMC Genomics *8*, 33.

Nussinov, R., Ma, B., Tsai, C.J., and Csermely, P. (2013). Allosteric conformational barcodes direct signaling in the cell. Structure *21*, 1509–1521.

Velankar, S., Dana, J.M., Jacobsen, J., van Ginkel, G., Gane, P.J., Luo, J., Oldfield, T.J., O'Donovan, C., Martin, M.-J., and Kleywegt, G.J. (2013). SIFTS: Structure Integration with Function, Taxonomy and Sequences resource. Nucleic Acids Res. *41*, D483–D489.

Zhao, M., Sun, J., and Zhao, Z. (2013). TSGene: a web resource for tumor suppressor genes. Nucleic Acids Res. *41*, D970–D976.
