# Supplementary material for: Molecular Principles of Gene Fusion Mediated Rewiring of Protein Interaction Networks in Cancer
Source: Mol Cell. 2016 Aug 18;63(4):579–92. doi: 10.1016/j.molcel.2016.07.008 (PMC5003813; doi:10.1016/j.molcel.2016.07.008)
Supplement: Document S2. Article plus Supplemental Information [file mmc9.pdf]

Article

Molecular Cell

Molecular Principles of Gene Fusion Mediated Rewiring of Protein Interaction Networks in Cancer

Graphical Abstract

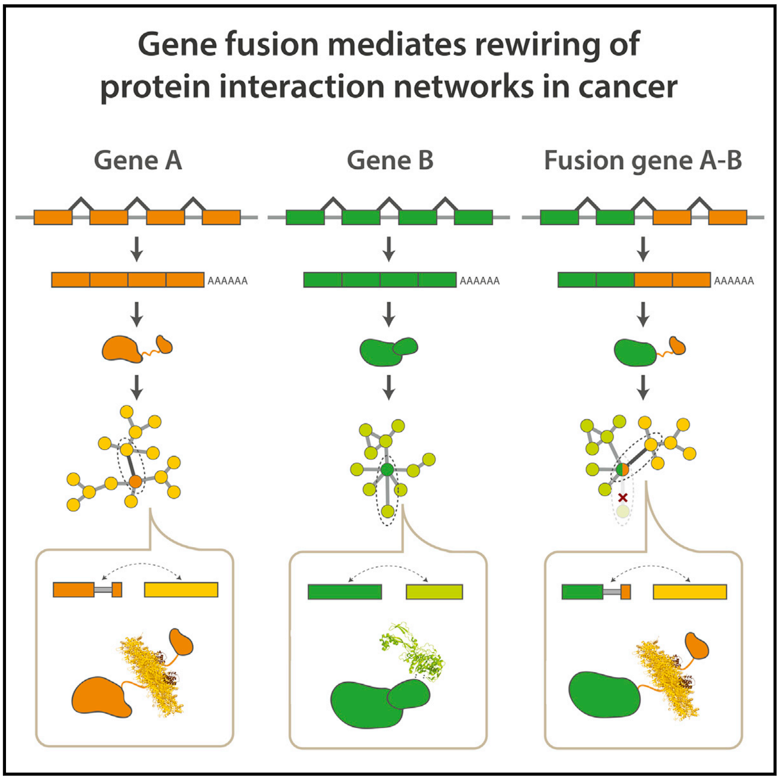

Highlights

- Parents of fusion proteins occupy central positions in protein interaction networks
- Parents are rich in interaction-mediating features, which are often lost via fusion
- Fusions preferentially join proteins with no previous connection in protein networks
- Fusion proteins escape regulation by losing post-translational modification sites

Authors

Natasha S. Latysheva, Matt E. Oates, Louis Maddox, ..., Marija Buljan, Robert J. Weatheritt, M. Madan Babu

Correspondence

natashal@mrc-lmb.cam.ac.uk (N.S.L.), madanm@mrc-lmb.cam.ac.uk (M.M.B.)

In Brief

The molecular mechanisms of fusion-mediated interactome disruption are currently unclear. Latysheva et al. find that fusion-forming proteins occupy central positions in interaction networks. They lose much of their extensive interaction-mediating ability and capacity for regulation upon fusion. These findings provide insights into how fusion proteins could rewire networks in cancer.

Latysheva et al., 2016, Molecular Cell *63*, 579–592
August 18, 2016 © 2016 The Authors. Published by Elsevier Inc.
http://dx.doi.org/10.1016/j.molcel.2016.07.008

CellPress

Molecular Principles of Gene Fusion Mediated Rewiring of Protein Interaction Networks in Cancer

Natasha S. Latysheva,[1,*] Matt E. Oates,[2] Louis Maddox,[1] Tilman Flock,[1] Julian Gough,[2] Marija Buljan,[1] Robert J. Weatheritt,[1,3] and M. Madan Babu[1,*]
[1]MRC Laboratory of Molecular Biology, Francis Crick Avenue, Cambridge CB2 0QH, UK
[2]Department of Computer Science, University of Bristol, Bristol BS8 1UB, UK
[3]The Donnelly Centre, University of Toronto, Toronto, ON M5S 3E1, Canada
*Correspondence: natashal@mrc-lmb.cam.ac.uk (N.S.L.), madanm@mrc-lmb.cam.ac.uk (M.M.B.)
http://dx.doi.org/10.1016/j.molcel.2016.07.008

SUMMARY

Gene fusions are common cancer-causing mutations, but the molecular principles by which fusion protein products affect interaction networks and cause disease are not well understood. Here, we perform an integrative analysis of the structural, interactomic, and regulatory properties of thousands of putative fusion proteins. We demonstrate that genes that form fusions (i.e., parent genes) tend to be highly connected hub genes, whose protein products are enriched in structured and disordered interaction-mediating features. Fusion often results in the loss of these parental features and the depletion of regulatory sites such as post-translational modifications. Fusion products disproportionately connect proteins that did not previously interact in the protein interaction network. In this manner, fusion products can escape cellular regulation and constitutively rewire protein interaction networks. We suggest that the deregulation of central, interaction-prone proteins may represent a widespread mechanism by which fusion proteins alter the topology of cellular signaling pathways and promote cancer.

INTRODUCTION

Fusion genes are hybrid genes formed from two previously independent parent genes. Historically, gene fusions have been viewed as common driver mutations in malignancies associated with blood, lymph, and bone marrow tissue, but are becoming increasingly recognized as important players in solid tumors (Mertens et al., 2015a, 2015b; Yoshihara et al., 2015). For example, translocation-induced gene fusions are found in about 90% of all lymphomas and over half of all leukemias (Lobato et al., 2008), and the *TMPRSS2-ERG* fusion is the most frequent genetic aberration in prostate cancer (Nam et al., 2007). In accord with their important role in oncogenesis, fusion transcripts and proteins have been utilized in many areas of clinical care, from biomarker development and diagnostics to acting as therapeutic targets (Kumar-Sinha et al., 2015; Mertens et al., 2015b). Yet, aside from a relatively small number of well-studied fusions, the functions of fusion proteins and the cellular context in which they operate remain unclear.

A variety of mechanisms can lead to the fusion of two genes, such as insertions, deletions, inversions, and translocations. Continuous transcription of neighboring genes (Varley et al., 2014) or *trans*- and *cis*-splicing of pre-mRNAs (Jividen and Li, 2014; Zhang et al., 2012) can also generate fusion transcripts and proteins. If fusion transcripts are translated, the resulting fusion proteins have the potential to redirect cellular signaling pathways and act as principal oncogenic drivers (see Watson et al., 2013; Yoshihara et al., 2015). Despite some concerns over whether certain putative fusion mRNAs may be artifacts of the sequencing procedure (Yu et al., 2014), the widespread finding of recurrent gene fusions in tumor samples, the clinical utility of an increasing number of gene fusions, and a growing body of literature on fusion protein functionality adds support to their potential for significant biological impact.

There are now approximately 10,000 known gene fusions, most of which have only recently been discovered using deep sequencing technology (Mertens et al., 2015a). The molecular functions of gene fusions, and the fusion proteins they encode, remain relatively poorly understood. Recent bioinformatics work on gene fusions (reviewed in Latysheva and Babu, 2016) has examined fusion protein domain content and recombination, reading frame conservation, intrinsic disorder at fusion junctions, and expression properties. However, the molecular principles of fusion-mediated rewiring of protein networks and how fusion proteins could disrupt native protein interactions remain unclear. Here, we devise a genome-scale computational data analysis framework to investigate the molecular principles by which fusion proteins affect protein interactions (Figures 1A and 1B). Understanding the structural features of fusion proteins, as well as the interactions that are recurrently disrupted or created as a result of fusion, will help clarify how fusions contribute to specific cellular phenotypes and influence cancer initiation and progression.

RESULTS

To compose a set of human fusion proteins, a list of fusion transcripts from the ChiTaRS v1 database (Frenkel-Morgenstern et al., 2013) was acquired and mapped onto Ensembl protein sequences (Experimental Procedures; Figure 1C). In this study,

CrossMark

This is an open access article under the CC BY license (http://creativecommons.org/licenses/by/4.0/).

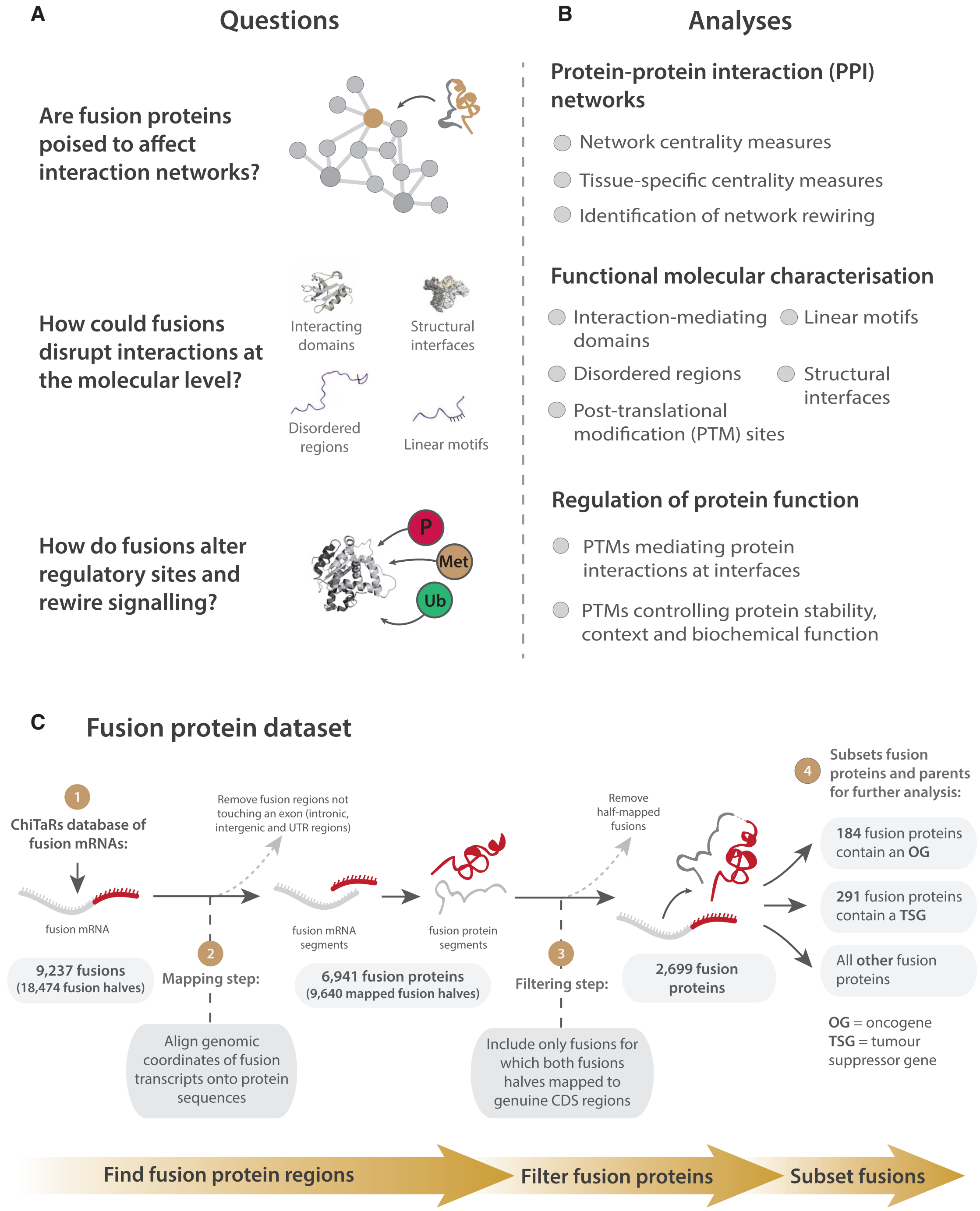

Figure 1. Study Outline

(A) Investigating how gene fusions and fusion proteins could affect molecular interactions in cancer.

(B) Summary of analyses employed.

(C) Description of processing procedure applied to the ChiTaRS database of fusion ("chimeric") mRNA sequences to obtain a data set of fusion proteins. See also Figures S1 and S2 and Table S1.

only fusions affecting protein-coding regions were examined. In total, we mapped 2,699 distinct fusion proteins derived from 3,279 genes (Table S1; fusion protein mappings are available as a web resource at http://fusion.d2p2.pro, integrated into the D2P2 database; Oates et al., 2013). Genes that form fusions ("parent genes") are enriched for functions related to translation, mRNA splicing, and the cell cycle, and for protein classes related to translation, acetyltransferase activity, and the binding of actin,

chromatin, and RNA (Table S1). Parent genes that form multiple fusions, especially five or more, are further enriched for functions relating to translation, RNA binding, and nucleic acid binding.

Gene fusion events can be summarized as a network, in which nodes indicate genes and a link between nodes indicates the occurrence of a fusion between genes. Our resulting network of gene fusions involving 3,209 genes (as gene symbols; Figure S1A) expands upon previous networks of ~300 gene fusions (Höglund et al., 2006; Mitelman et al., 2007); we confirm the presence of several major hubs, i.e., nodes with many edges (e.g., *MLL*, *ETV6*, *NUP98*, *EWSR1*, and *ALK*), and highlight novel fusion hubs (e.g., *COL1A1*, *HSP90AA1*, *MT1A*, *NCL*, and *AFF1*; Table S1; Figure S1A). The number of fusions formed for each gene follows a power law distribution (Figure S1B), with most parent genes forming few fusions (e.g., only 21 genes form ten or more fusion proteins). Over a third of known oncogenes (OGs) and a quarter of known tumor suppressor genes (TSGs) form fusions in this data set (Figure S1).

Parent Proteins Have More Central Roles in Protein Interaction Networks and Are Expressed at Higher Levels

To examine whether parent genes encode proteins with central positions in the human interactome, a high-confidence data set of human protein-protein interactions (PPIs) (Wang et al., 2012) was analyzed. In addition to a much higher number of interaction partners (node degree; Figure 2A), parent proteins have a significantly higher tendency to interconnect interaction clusters, as quantified by betweenness centrality, which measures the extent to which a given node in a network lies on the shortest paths between all other nodes (Figure 2B). Furthermore, parent proteins have higher Kleinberg's hub scores (see Experimental Procedures), which measure a protein's connection to network hubs (Figure 2C). Compared to central non-parents, the most central parent proteins were more likely to be involved in functions such as mRNA splicing, cell proliferation, DNA replication, and repair (Table S2).

We observed that parent mRNAs and proteins are more abundant compared to non-parents (~3-fold difference between averages; Figures S2A and S2B) in medulloblastoma cell lines (Vogel et al., 2010). Additionally, parent proteins have very similar half-lives to non-parent proteins (Figure S2C). Further, by integrating data on 12 oncogenic signaling blocks (Cui et al., 2007), we find that parent proteins are over twice as likely to be involved in signaling processes implicated in oncogenesis ($\chi^2 = 29.5$, df = 1, and $p = 5.7 \times 10^{-8}$) (Figure S2D) and are over 2.5 times as likely to be genes essential for cellular viability ($\chi^2 = 396.8$, df = 1, and $p < 2.2 \times 10^{-16}$) (Figure S2E). Although these trends need to be analyzed in different tissues, these results suggest that altering parent proteins could have a major effect on critical cellular functions and for a sustained period of time.

Parent genes were grouped into OG parent genes, TSG parent genes, and all other parent genes (Figure S3A). Parent genes that are neither OGs nor TSGs possess significantly higher network centrality than non-parent genes, indicating that centrality is a feature of parent genes more broadly and not simply reflective of the centrality of OGs and TSGs. Further, parent OGs and TSGs tend toward higher centrality than non-parent OGs and TSGs, respectively (Figure S3B). For example, average centrality measures for parent TSGs are approximately 30% higher than non-parent TSGs. Replicate network centrality calculations on two additional PPI data sets—the consensus network used in further analyses (see below; Bossi and Lehner, 2009) (Figure S3C) and an unbiased interaction network derived using mass spectrometry (Huttlin et al., 2015) (Figure S3D)—were consistent with those described above.

Parent Proteins Have Higher Centrality in the Interaction Networks of Cancer-Associated Cell and Tissue Types

Next, the role of parent proteins in tissue-specific protein interaction networks (Bossi and Lehner, 2009) was examined. PPIs involving parent proteins are present in more human tissues (median of 64 of 79 tissues, compared to 52 of 79 for non-parents; $p < 2.2 \times 10^{-16}$; Figure 2D), indicating that fusion events do not only affect tissue-specific interactions. Parent proteins consistently have on average ~5 additional interaction partners across most tissues (Figure 2E). Interestingly, the tissues and cell types with the highest degrees for parent proteins—e.g., B and T cells, bone marrow cells, and blood cells—are cell types often associated with fusion-induced cancers (gold dots, Figure 2E). Furthermore, parent proteins in the five cancer cell types in the data set (teal dots) have on average 9.1% higher degree than non-cancer cells and 12.1% higher degree than the set of non-cancer and non-blood/bone/lymph cell types (Table S3). This trend is not observed for betweenness (Figure S3E), but is for hub scores (Figure S3F), which may indicate that gene fusions in cancer may preferentially affect nodes of high degree (either directly or indirectly) rather than alter global network cohesion. Fusions could therefore be especially disruptive in tissues with interaction networks containing proteins with unusually high degree. Finally, fusion transcripts detected in cell lines of metastatic tumor origin may have parent genes with higher centrality compared to those from primary tumors (Figures S4A–S4D; Supplemental Information), suggesting a possible connection between cancer aggressiveness and parent centrality. Although this trend was not observed in the mass spectrometry PPI data set (Huttlin et al., 2015; data not shown), the concept of a link between cancer stage and the roles of parent proteins in PPI networks may be relevant in specific contexts (e.g., certain cancer types).

Parent Proteins Are Unstructured and Enriched for Interaction-Mediating Domains, which Are Preferentially Excluded from Fusion Proteins

The structural features of parent proteins and their retention within fusion proteins were investigated (Figures 3A–3L and S5A–S5K). In agreement with a previous study (Hegyi et al., 2009), parent proteins in our expanded data set (3,279 parent proteins versus 406) have significantly higher intrinsic structural disorder scores than non-parents (Figure S5A): OG parents have on average 1.27 × (0.39 versus 0.31; $p = 2.8 \times 10^{-4}$; and pairwise Wilcoxon rank-sum tests with Holm multiple testing correction), TSG parents 1.15 × ($p = 1.5 \times 10^{-3}$), and other parents 1.13 × ($p < 2 \times 10^{-16}$) higher disorder compared to non-parents. Parent OGs and TSGs are approximately equally disordered as

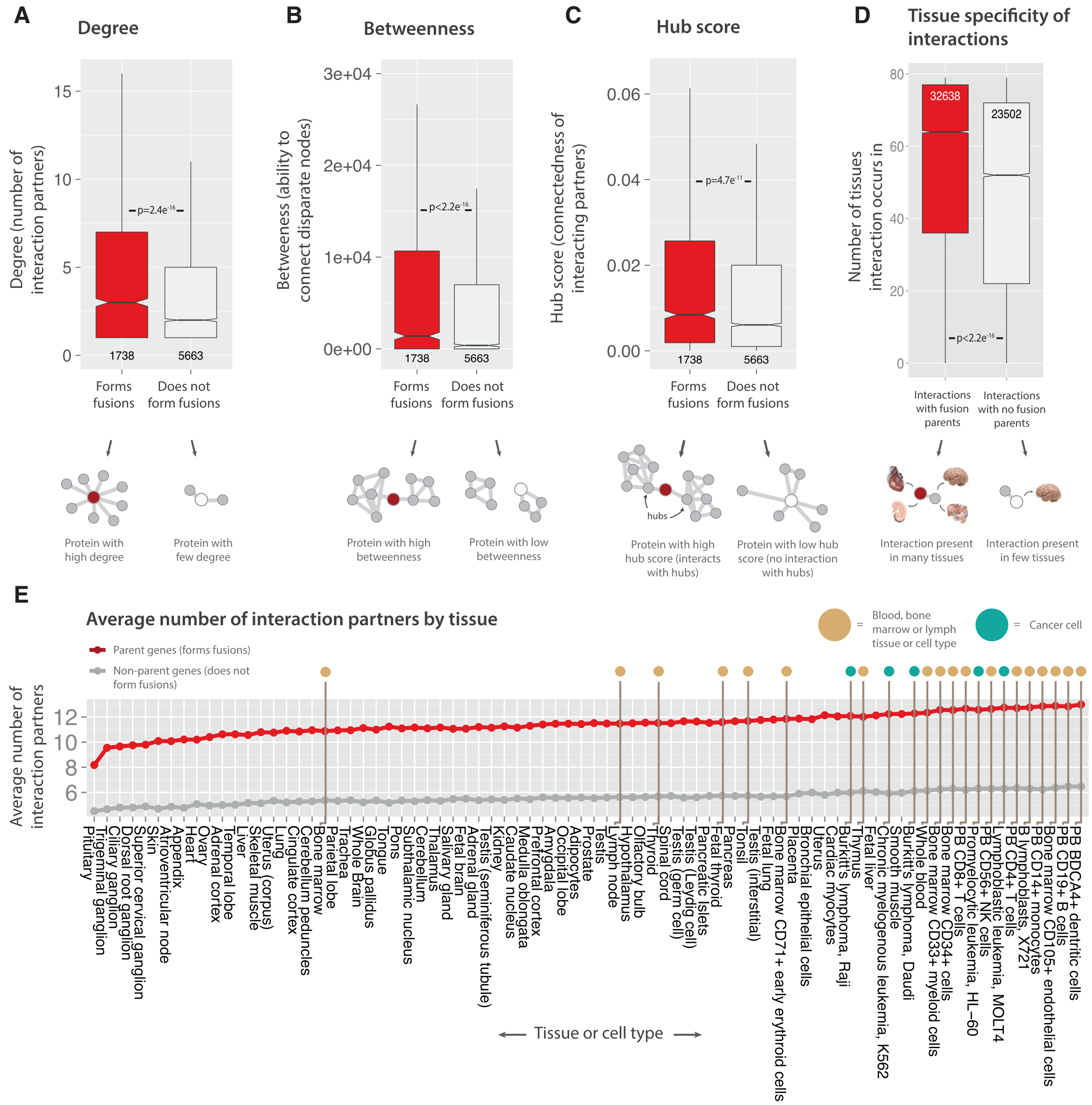

Figure 2. Network Centrality of Parent Genes and Proteins

(A–C) Parent genes possess more interaction partners in PPI networks (A), have higher betweenness centrality (B), and higher hub scores (C).

(D) PPIs involving parent proteins occur in more human tissues than interactions not involving parent proteins.

(E) The average number of interaction partners for parent proteins and all other proteins by tissue or cell type (gold = blood, bone marrow, and lymph tissues and teal = cancer cells). Throughout this study, distribution outliers are excluded from boxplots for presentation purposes, but included in statistical analyses. See also Figures S3 and S4 and Tables S2 and S3.

non-parent OGs and TSGs (Figure S5B), as are included versus excluded fusion protein segments (Figure S5C). This suggests that any observed enrichment of linear motifs and post-translational modifications (PTMs) in included segments (see below), which are features correlated with disorder (Davey et al., 2012), are not simply due to included segments being more disordered. Throughout the structural feature calculations, densities instead of counts are used to control for protein length.

Using a database of PPIs defined at the structurally resolved level of domains (Meyer et al., 2013), we investigated parent versus non-parent densities of interaction-mediating domains (IMDs). Parent proteins, especially OG and TSG parent proteins, have higher densities of IMDs (Figure 3A). On average, compared to non-parent proteins, OG parents have 4.6×, TSG parents 2.7×, and other parents 1.5× the IMD densities (all corrected p values: $<2.2 \times 10^{-16}$). There is a slight tendency for parent OGs to have higher IMD densities than non-parent OGs (on average 1.3×; $p = 9.1 \times 10^{-3}$; Figure S5D). Hence, although parent proteins are generally more intrinsically disordered, they are also enriched in structured domains that mediate protein interactions. IMDs tend to largely be excluded from fusion proteins (Figure 3B; Table S4). OG parent proteins, in contrast to

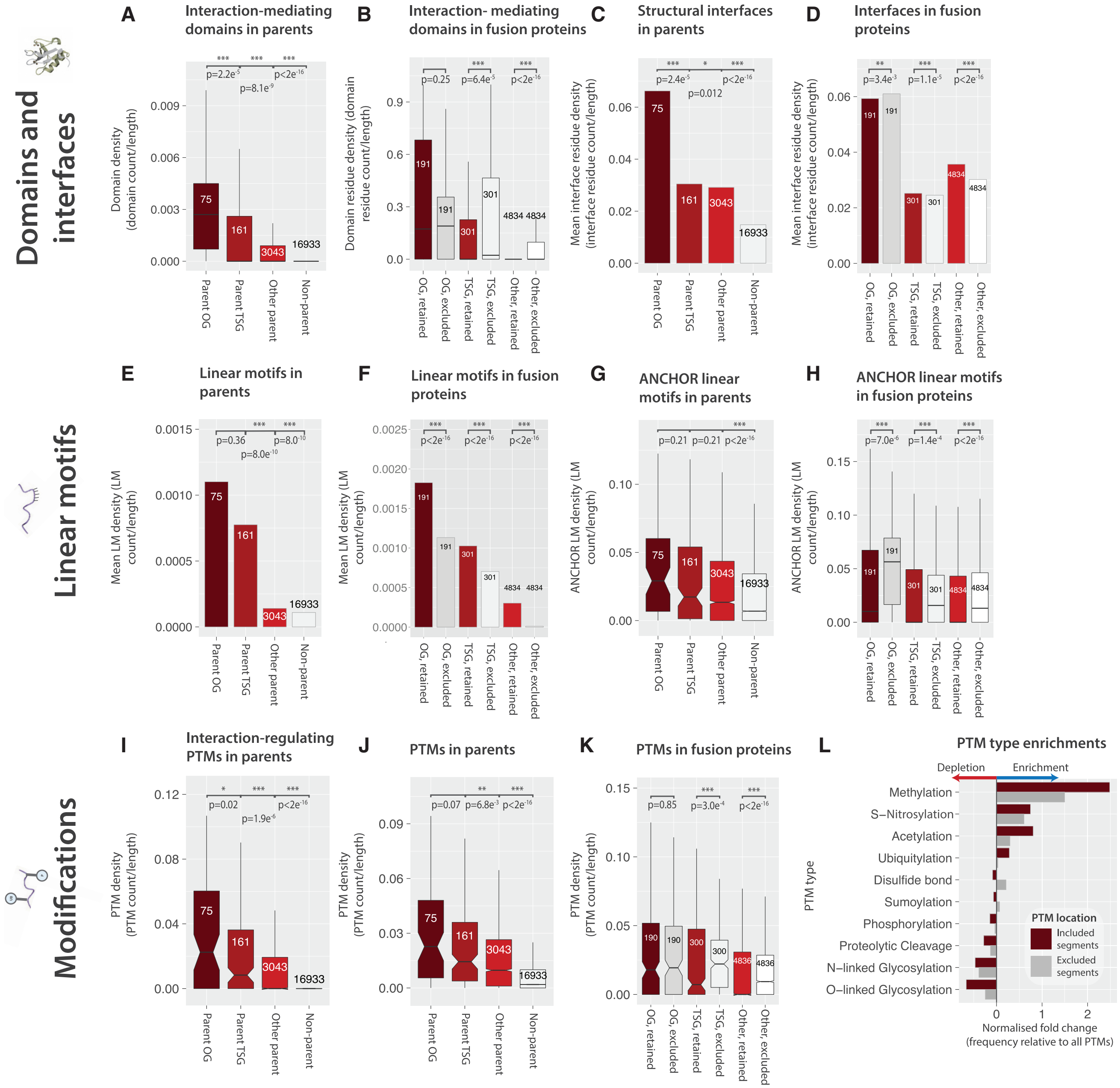

Figure 3. Interaction-Mediating Molecular Features in Fusion Proteins

(A and B) IMDs in parent proteins (A) and fusion proteins (B).

(C and D) The PPI interface residues in parent proteins (C) and fusion proteins (D) are shown.

(E and F) The ELM LMs in parent proteins (E) and fusion proteins (F) are shown.

(G and H) The predicted ANCHOR LMs in parent proteins (G) and fusion proteins (H) are shown.

(I) The putative interaction-regulating PTMs in parent proteins are shown.

(J and K) Other PTM sites in parent proteins (J) and fusion proteins (K) are shown.

(L) The PTM type enrichments in included and excluded parent protein segments are shown. Within each subplot, Holm's sequential Bonferroni correction for multiple testing was applied.

See also Figure S5 and Tables S4 and S6.

TSG and other parent proteins, tend to retain IMDs upon fusion. Overall, the most frequently retained IMDs include RNA-recognition, tyrosine kinase, pleckstrin homology (signaling and cytoskeleton), and SH3 and SH2 signaling domains (Table S4). The average level of domain truncation upon transfer varies significantly by domain type, and the most intact IMDs which

occur $\geq$10 times include ubiquitin conjugating domains, the ubiquitin-like PB1 domain (a specificity adaptor to kinases), and the proliferation modulating S_100 domain. Parents that repeatedly donate large portions of IMDs are enriched for functions in translation, cell structure morphogenesis, and cell cycle and protein modification (Table S4).

Transfer of IMDs Can Create Novel Interactions and Preserve Important Natural Interactions

The repeated inclusion of large portions of specific IMDs in fusion proteins is interesting for two reasons (Figure 4A). First, it can point to the importance of a particular domain-domain interaction (DDI) for a fusion protein's function. Second, as a result of the fusion, a novel interaction-like link can occur between the interaction partner of the included domain and the fusion partner. We map which domain-mediated PPIs are repeatedly conserved in fusion proteins (Figures 4B and S6A; Table S5). We find that 192 IMD-mediated PPIs are recurrently retained in fusion proteins and comment on the most frequently conserved DDIs (see the Figure S6A legend).

We also map novel protein links that are created through IMD transfer (Figures 4C and S6B; Table S5). A protein interaction "link" was drawn between proteins A and B if there existed some fusion protein B-C, where C normally interacts with A and at least 90% of C's IMD was retained (Figure 4A). Of the 126 novel links, 116 (92%) do not normally occur in the cell. The most frequent novel links include many connections for BCR, with the newly linked proteins being enriched for functions in cell proliferation and cellular component movement (Table S5), and 11 new connections for the nuclear trafficking protein TPR, including eight tyrosine protein phosphatases (Figures 4C and S6B). Certain fusion-induced novel links are recurrent, e.g., fusion proteins involving both EML4 and TFG lead to the gain of similar links (i.e., connections to receptor-type protein tyrosine phosphatases PTPRB, PTPRG, and PTPRJ).

Fusion-Generated Novel Links Disproportionately Connect Proteins that Are Distant in the Interaction Network

We examined the distance between the protein pairs in the novel links set in a non-diseased PPI network. Where a path existed between the novel links pairs, the distance was overall slightly shorter than in other protein pairs in the network (Figure S6C). However, fusion was found to disproportionately connect proteins which normally reside in separate sections of the interactome, whereas only 10.7% of protein pairs in the PPI network had no connecting path, 29.3% of protein pairs in the novel links set had no previous connecting path (Fisher's exact test on contingency table, odds ratio = 3.47, $p = 3.0 \times 10^{-8}$) (Figure S6D). We examine the 34 newly connected protein pairs in Figure S6E (see the legend).

Independent Structural Evidence Supports the Potential of Fusion Proteins to Disrupt PPI, Protein-RNA Interactions, and Protein-DNA Interactions

Structural interfaces in fusion proteins were identified by analyzing the Protein Interfaces, Surfaces, and Assemblies (PISA) database, which houses macromolecular interfaces (involving proteins, RNA, and DNA) in the Protein Data Bank (PDB). Parent proteins in the PDB contain more interface-forming residues (Figure 3C). On average, 1.5% of residues in non-parents form interfaces, and OG parents have on average 4.5×, TSG parents 2.1×, and other parents 2.0× this PISA residue density. Parent OGs have 2.4× the average interface residue density of non-parent OGs ($p = 3.6 \times 10^{-5}$; Figure S5E). Interface residue densities on included and excluded segments of parent proteins are similar (Figure 3D), though the distribution is skewed toward exclusion (Figure S5F). The 302 parent proteins which donate ten or more interface-forming residues to fusion proteins are enriched for functions relating to cell cycle signaling, carbohydrate and lipid metabolism, cellular component morphogenesis, and cell death (Table S6).

Parent Proteins Are Enriched in Interaction-Mediating Short Linear Motifs, which May Be Preferentially Excluded from Fusion Products

Linear motifs (LMs) are short sequence motifs, usually <10 residues, often found in intrinsically disordered regions (Tompa et al., 2014). Using 1,410 experimentally validated LMs from the ELM database (Dinkel et al., 2014) and over a million putative LMs identified using the ANCHOR program (Dosztányi et al., 2009), we tested for enrichment of LMs within parent proteins compared to all other proteins. Parent proteins have more experimentally verified LMs on average (Figure 3E), with OG and TSG parents harboring more motifs. Although most parents have zero experimental LMs due to the small size of this data set, on average, OG parents have 10.1× ($p < 2 \times 10^{-16}$), TSG parents 7.1× ($p < 2 \times 10^{-16}$), and other parents 1.3× ($p = 8.0 \times 10^{-10}$) the LM density of non-parents. Parent TSGs have slightly higher LM densities compared to non-parent TSGs (Figure S5G). Fusion proteins tend to retain ELM LMs, as shown by higher mean LM densities in included segments (Figure 3F). Parent proteins, which donate ELM LMs, function in the regulation of cell death, the stress response, protein metabolism, and nucleic acid binding (Table S6). Similarly, the expanded ANCHOR data shows higher densities of LMs in parents (Figure 3G), though parent OGs and TSGs have similar densities to the non-parent categories (Figure S5H). Interestingly, the larger ANCHOR data set shows a strong trend toward the exclusion of LMs (Figure 3H). Either trend implies that fusion substantially disrupts transient interactions mediated by LMs.

PTMs that Regulate Protein Interactions Are Enriched in Parent Proteins

We mapped putative interaction-regulating PTMs (PTMcode v2 database; Minguez et al., 2015) onto proteins and found that compared to non-parents, OG parents have on average 4.6×, TSG parents 3.5×, and other parents 2.2× the PTM density (all corrected $p < 2 \times 10^{-16}$; Figure 3I). Parent TSGs have slightly more interaction-regulating PTMs compared to non-parent TSGs (1.5×, $p = 0.03$; Figure S5I). These PTM sites overall tend toward exclusion from fusion proteins (Figure S5J), though the retention and loss is comparable in OG and TSG parents.

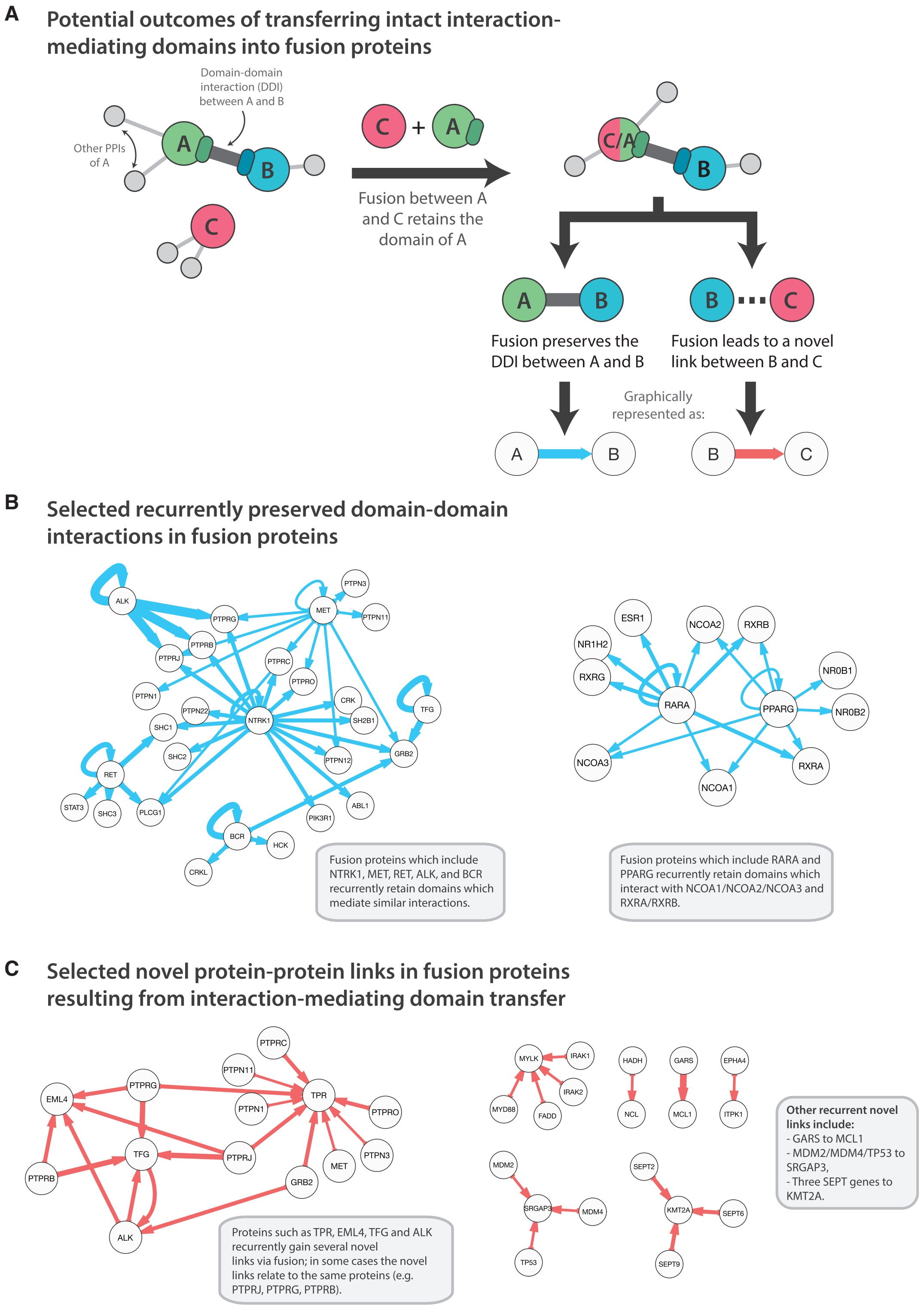

Figure 4. Retained and Novel PPI in Fusion Proteins

(A) The repeated inclusion of large portions of specific IMDs in fusion proteins can lead to the retention of domain-mediated interactions or the creation of novel interaction-like links between proteins.

(B and C) Subsets of the recurrently retained domain-mediated PPIs (B) and novel links (C) are shown.

See also Figure S6 and Table S5.

Parent Proteins Are Enriched in PTM Sites, and Fusion Proteins Tend to Selectively Escape Regulation by PTMs

In addition to regulating protein interactions, post-translational and co-translational modification sites can regulate protein stability (e.g., by ubiquitination), subcellular localization (e.g., N-myristoylation), and protein function (e.g., acetylation). Parent proteins have significantly more PTMs (Figure 3J) compared to non-parents (on average 0.009 PTMs/residue): OG parents have 3.5×, TSG parents 3.5×, and other parents 2.3× the PTM densities of non-parents (all corrected $p < 2 \times 10^{-16}$). This suggests that the function, stability, and subcellular location of parent proteins are extensively regulated by PTMs. Further, on average, parent OGs have 1.5× ($p = 7.5 \times 10^{-3}$) the PTM content of non-parent OGs, and parent TSGs have 2.1× ($p = 1.4 \times 10^{-5}$) the PTM content of non-parent TSGs (Figure S5K). PTMs are generally excluded from fusion proteins, though not in OG parents (Figure 3K). The selective exclusion of PTM sites suggests that fusion proteins tend to escape regulation by signaling pathways. TSG parents experience the heaviest loss of PTMs, with excluded segments having over triple the median PTM density of included segments (excluded: 0.022 PTMs/residue; included: 0.007; $p = 3.0 \times 10^{-4}$; Figure 3K). Parent proteins which retain at least 90% of their PTM content are enriched for functions in translation, ion transport, and metabolism (Table S6), while parent proteins which lose at least 90% of their PTMs have a wide range of functions, including splicing and cell matrix adhesion.

Next, we examined the PTM profiles in included and excluded fusion protein segments (Experimental Procedures; Figure 3L). Certain PTM types (e.g., S-Nitrosylation) occur in either parental segment more frequently than expected given the global frequencies of all PTMs in dbPTM, while other PTM types (e.g., methylation and acetylation) showed marked presence/absence patterns based on segment inclusion (Table S6).

Fusion Can Lead to the Gain and Loss of Ubiquitination Sites, which May Deregulate the Activity of OGs and TSGs

Ubiquitination (UB) sites are of particular interest since their loss and gain upon fusion could "upregulate" OG activity or "downregulate" TSG activity, due to the role of UB sites in mediating protein stability and degradation. We find 14 fusion proteins in which OGs lose $\geq$5 UB sites and ten fusion proteins in which a TSG gains $\geq$5 UB sites (Table 1). As an illustrative example, we profile the well-known *EWSR1-FLI1* gene fusion from Ewing's sarcoma (Figure 5A). The specific pattern of segment retention in EWSR1-FLI1 fusion proteins leads to UB site loss, which may confer increased stability onto the fusion product, adding to the known oncogenic mechanism of transcriptional deregulation. Notably, decreased UB-mediated degradation of ETS family transcription factors (e.g., FLI1) has been linked to cancer (Vitari et al., 2011). Conversely, one of the most extreme examples of UB site gain by a TSG occurs in the previously unstudied *ATP50-TGFB1* fusion (Figure 5B), which results in the amalgamation of a heavily ubiquitinated segment with a short portion of the TGFB1 tumor suppressor domain, hinting at a fusion-mediated loss of TSG function. TGF-β signaling is known to inhibit cell proliferation and is normally tightly regulated by UB (Huang and Chen, 2012). OG parents do not lose and TSG parents do not gain UB sites more often than expected (data not shown), but individual cases identified here (Table 1) could be of substantial biological interest for follow-up studies.

Fusions Involving Transcription Factors Are Linked to Significant Alterations in Downstream Target Gene Expression Levels

To investigate the potential downstream network rewiring effects due to fusion events, we investigated whether fusions involving transcription factors (TFs) are associated with downstream expression changes in the TFs' regulatory targets. TCGA tumor samples with TF-containing fusion transcripts and paired normal controls were identified (Experimental Procedures). The regulatory target genes of TFs were acquired from the TRRUST database (Han et al., 2015). Differential gene expression (DGE) values were calculated (absolute $\log_2$ fold change between diseased and healthy samples). The targets of TFs had significantly (i.e., corrected $p < 0.05$) higher DGE values in five of the eight paired breast cancer samples when compared to all other genes (Figure S7). For example, four fusion transcripts containing TFs were detected in patient TCGA-GI-A2C9; these four TFs together affected 51 mapped regulatory targets, the mean (absolute $\log_2$) DGE of which is 2.0× the mean DGE of all other genes (Table S7; corrected $p = 9.6 \times 10^{-5}$). Across the eight available biospecimen pairs, the average DGE of TF targets is 1.41× (mean) and 1.45× (median) the DGE of all other genes.

DISCUSSION

Many disease states result from altered dynamics of complex regulatory and signaling interactions. Representing interactions as networks provides a conceptual framework for understanding how mutations in proteins can affect entire cellular systems and cause disease (Wang et al., 2011; Wu et al., 2010), especially when combined with structural analyses of interacting proteins (Sudha et al., 2014; Wang et al., 2012). Here, we investigated the interaction properties and structural features of thousands of putative fusion proteins. Based on our observations, we delineate genome-scale molecular principles by which gene fusions can affect protein networks, rewire signaling pathways, and contribute to disease (Figure 6). These trends will be useful for setting novel gene fusions into context, building on the performance of previous driver gene fusion prioritization algorithms (Abate et al., 2014; Shugay et al., 2013), and interpreting studies of fusion protein functionality.

Fusion Preferentially Affects Highly Central, Interaction-Prone Proteins

Although it is likely that not all of the analyzed fusion proteins drive disease (e.g., genomic instability can produce passenger fusions; Mertens et al., 2015a), parent proteins are nonetheless enriched for a wide variety of interaction-prone elements, such as IMDs, interface-forming residues, LMs, and PTM sites that regulate PPIs. The observed density of interaction-mediating features in parent proteins is in accord with their centrality in interaction networks. These results are consistent with other computational work on disease mutations, which have shown

Table 1. OGs Losing ≥5 UB Sites and Tumor Suppressor Genes Gaining ≥5 UB Sites as a Result of Fusion Events

Fusion Accession[a]	OG	Description	Number of UB Sites Lost	Length of OG Retained Segment	Fusion Partner	Description of Fusion Partner
BF736842	EGFR	epidermal growth factor receptor	17	25	SLC12A9	solute carrier family 12, member 9
AK098472	CTNNB1	catenin (cadherin-associated protein), beta 1, and 88 kDa	9	420	RP11-345J4.5	bolA-like protein 2
BE176861	COPS5	COP9 signalosome subunit 5	9	112	HNRNPH3	heterogeneous nuclear ribonucleoprotein H3 (2H9)
BE176782	COPS5	COP9 signalosome subunit 5	9	112	HNRNPH3	heterogeneous nuclear ribonucleoprotein H3 (2H9)
BG953255	CTTN	cortactin	9	21	MYC	v-myc avian myelocytomatosis viral OG homolog
BP430745	CSE1L	CSE1 chromosome segregation 1-like (yeast)	7	41	UGP2	UDP-glucose pyrophosphorylase 2
CN278368	TRIM32	tripartite motif-containing protein 32	7	36	DDX21	DEAD (Asp-Glu-Ala-Asp) box helicase 21
CV340327	ERBB2	v-erb-b2 avian erythroblastic leukemia viral OG homolog 2	6	21	NOMO1	NODAL modulator 1
BE273347	DCUN1D1	DCN1, defective in cullin neddylation 1, and domain containing 1	6	24	QTRT1	queuine tRNA-ribosyltransferase 1
BC001010	CDK4	cyclin-dependent kinase 4	6	30	RPL4	ribosomal protein L4
AW371253	ERBB2	v-erb-b2 avian erythroblastic leukemia viral OG homolog 2	5	49	RABGAP1	RAB GTPase activating protein 1
U08818	MET	met proto-OG	5	380	MIR548F1	microRNA 548f-1
U19348	MET	met proto-OG	5	380	MIR548F1	microRNA 548f-1
DA624159	TFG	TRK-fused gene	5	90	GPR128	G protein-coupled receptor 128
**Fusion Accession**	**Tumor Suppressor Gene**	**Description**	**Number of UB Sites Gained**	**Length of TSG Retained Segment**	**Fusion Partner**	**Description of Fusion Partner**
CD368725	TGFB1	transforming growth factor, beta 1	13	45	ATP50	ATP synthase, H^+ transporting, mitochondrial F1 Complex, and O subunit
DB041801	SMARCA4	SWI/SNF related, matrix associated, actin dependent regulator of chromatin, subfamily a, and member 4	9	78	UBB	ubiquitin B
BP213958	ARID1A	AT rich interactive domain 1A (SWI-like)	6	34	DNAJA2	DnaJ (Hsp40) homolog, subfamily A, and member 2
DB120764	EEF1A1	eukaryotic translation elongation factor 1 alpha 1	6	4	HIST1H2AM	histone cluster 1, H2am
BG035867	EIF1	eukaryotic translation initiation factor 1	6	38	RALY	RALY heterogeneous nuclear ribonucleoprotein
AB209020	GJA1	gap junction protein, alpha 1, and 43 kDa	6	136	IFT140	intraflagellar transport 140
BG926120	PDCD4	programmed cell death 4 (neoplastic transformation inhibitor)	5	110	GAPDH	glyceraldehyde-3-phosphate dehydrogenase
BC001412	EEF1A1	eukaryotic translation elongation factor 1 alpha 1	5	462	LASP1	LIM and SH3 protein 1
BQ962146	E2F1	E2F TF 1	5	8	RDH11	retinol dehydrogenase 11 (all-*trans*/9-*cis*/11-*cis*)
CK004088	NDRG2	NDRG family member 2	5	153	RPL38	ribosomal protein L38

[a]ChiTaRS fusion event accessions are listed along with affected genes, retained segment lengths, and tallies of UB site gain or loss.

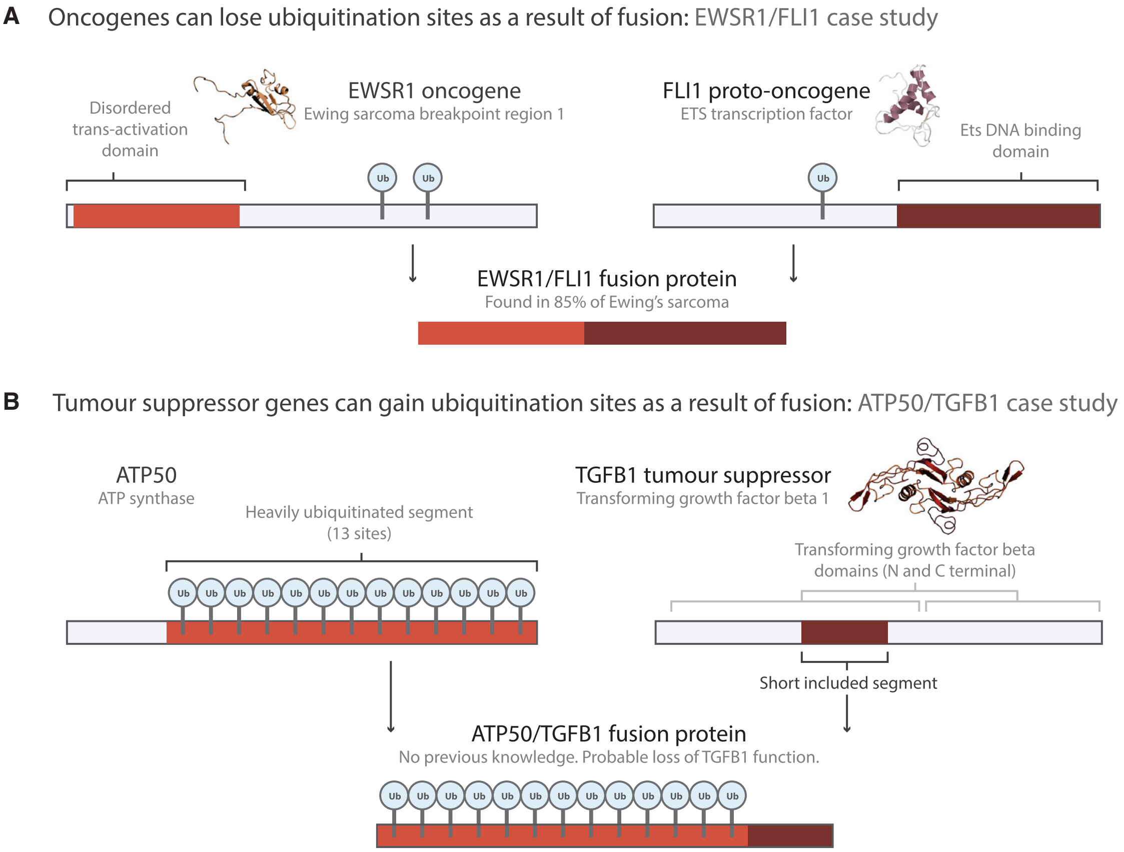

Figure 5. Fusion-Induced UB Site Gain and Loss in Cancer-Associated Proteins

Fusion proteins involving OGs and TSGs can lead to the loss or gain of ubiquitination sites.

(A) Example of an OG losing UB sites upon fusion.

(B) Example of a TSG gaining UB sites upon fusion. The protein structure cartoons are of EWSR1 (PDB: 2CPE), FLI1 (PDB: 1FLI), and TGFB1 (PDB: 1KLA).

that disease-related in-frame mutations (Wang et al., 2012) and disease-causing non-synonymous single nucleotide polymorphisms (David et al., 2012) are preferentially located on PPI interfaces. Finally, the finding that many parent genes are essential genes dovetails with the concept of "edgetic" perturbations in cancer, i.e., mutations that disrupt specific interactions (or edges) of proteins rather than the entire node (Charloteaux et al., 2011; Rolland et al., 2014; Wang et al., 2015), given that disrupting essential genes is associated with lethality, fusion may offer an opportunity to disrupt only a portion of an essential protein's function, such as specific interactions.

Network disruption may play a role in fusion proteins that first appear to have relatively simple mechanisms of oncogenesis (Figure 6A), for example, the concurrent rewiring of signaling pathways can be critical for BCR-ABL1 mediated transformation (Pawson and Warner, 2007). Importantly, targeting the interacting partners or downstream signaling of fusion proteins could be a fruitful area for therapeutic agent development (see Tognon et al., 2011). In this context, our observation that TF fusions significantly perturb target gene expression in breast cancer lends further weight to the signaling perturbation capabilities of fusion events.

Fusion Results in a Loss of Parental Interaction-Mediating Features and Regulatory Sites

Although parent proteins are enriched for interaction-mediating features, the segments of parents that are included within fusion proteins appear to be depleted of functional regions (though OG parents retain more of these features than other parents). Examining specific cases of fusion-mediated loss and gain of molecular features (Figures 3A–3L), as well as interaction preservation and creation (Figures 4A–4C), is a rich resource for hypothesis generation. For example, fusion proteins characterized by the repeated inclusion of largely complete tyrosine kinase domains (e.g., Figure 6B) could be promising targets for kinase inhibitors. Proteins dependent on the function of several distinct molecular features (such as the interface residues and nuclear import/export signal motifs in nucleophosmin; Figure 6B), as well as proteins sensitive to changes in PTM content (such as EWSR1; Figure 6C), may be especially disrupted by fusion events.

Although we largely addressed each interaction-mediating and regulatory molecular feature of parent and fusion proteins separately, these entities are not independent. For instance, LMs tend to form interactions conditionally on PTM site status

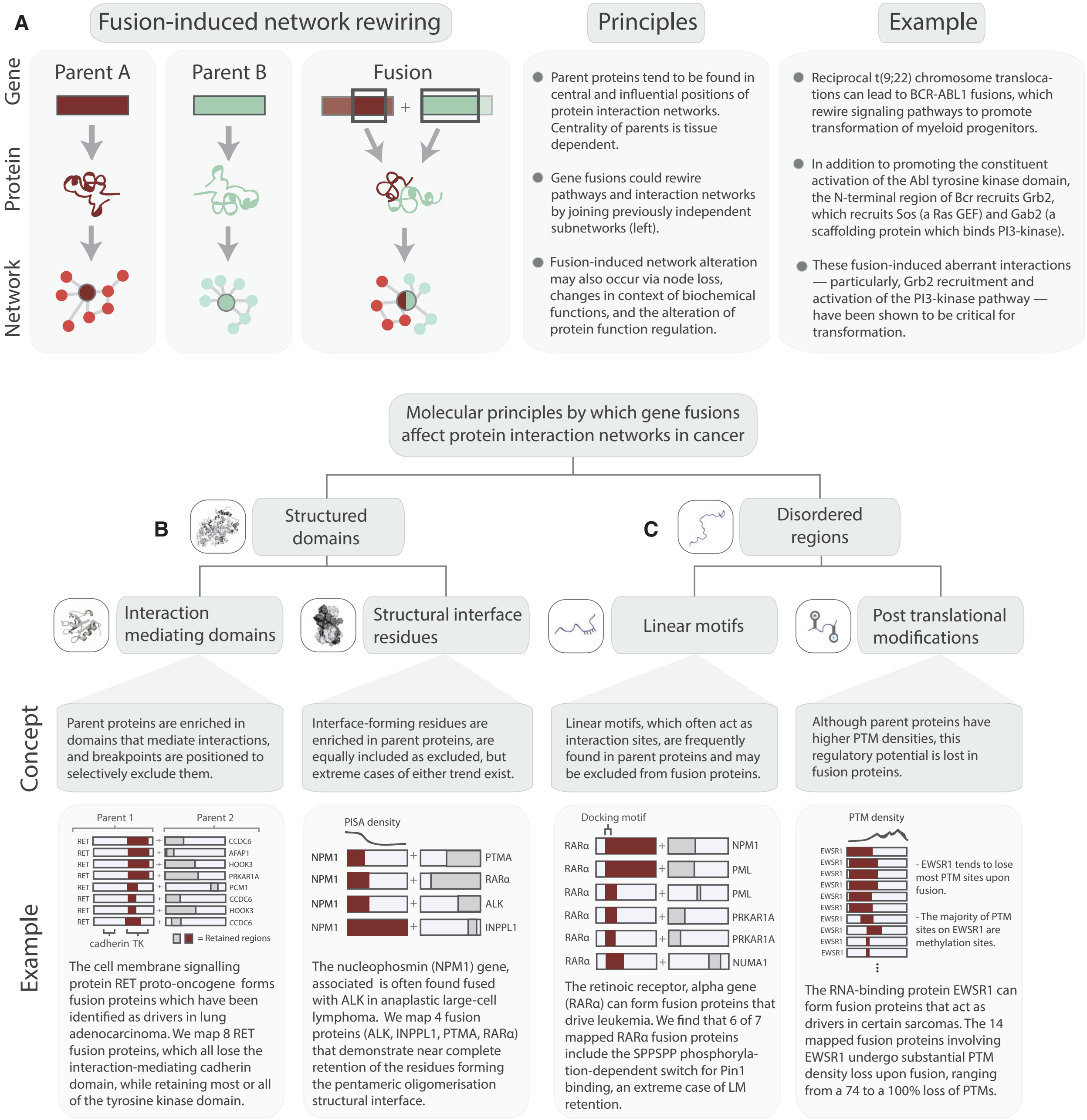

Figure 6. Molecular Principles by which Gene Fusions Can Alter Protein Interaction Networks in Cancer

(A) Fusion tends to involve highly central proteins in interaction networks and can alter networks by several mechanisms. Rewiring effects can play key roles in seemingly straightforward fusion events, as in the constitutive kinase activation found in the BCR-ABL1 fusion.

(B and C) More generally, fusion can affect molecular interactions of proteins by shuffling interaction-prone regions within ordered (B) and disordered (C) protein segments.

See also Figure S7 and Table S7.

(Van Roey et al., 2013). For example, the retinoic receptor alpha gene (*RARα*) encodes a LM that acts as a phosphorylation-dependent switch for binding Pin1. RARα forms driver fusion proteins in acute promyelocytic leukemia, for which Pin1 suppression is used as a treatment (Gianni et al., 2009). We find a RARα fusion protein that excludes the LM in question (Figure 6C), which could correspond to a treatment resistant patient. Knowledge of the specific retained sequence of fusion proteins has previously been observed to be key to patient treatment (Robinson et al., 2011).

Conclusions

Our findings demonstrate that proteins that form fusions tend to be highly interactive and positioned in critical regions of PPI networks. Disruption of such proteins may alter the topology of signaling and regulatory pathways of cells and promote cancer. A detailed understanding of the molecular impact of the rewired network will be helpful for future drug discovery studies. For example, in cases where driver fusion proteins retain the ability to form interactions, their carcinogenic activity could be reduced by the targeted disruption of specific interaction interfaces with small molecules (Cierpicki and Grembecka, 2015; Jin et al., 2014; Kuenemann et al., 2015). Additionally, recent methodological advances in therapeutically degrading specific proteins in vivo (Bondeson et al., 2015; Winter et al., 2015) could be instrumental to targeting oncogenic fusion proteins that have escaped normal regulatory pathways.

EXPERIMENTAL PROCEDURES

Database Identification, Processing, and Integration

To compose a set of human fusion proteins, we acquired a database (ChiTaRS v1 database; Frenkel-Morgenstern et al., 2013) of 9,237 fusion mRNAs. The fusion transcripts were mapped onto known proteins in the Ensembl database using ChiTaRS genomic coordinates and segments that mapped to non-exonic regions (intronic, UTR, or intergenic sequences) were discarded. The resulting data set maps all fusion protein segments defined at the DNA/gene, mRNA, and protein levels (Table S1). We limit our analysis to fusion proteins in which both parents were mapped to known Ensembl proteins. Fusion protein mapping information is made available via a web server (http://www.fusion.d2p2.pro). A fusion network of all gene fusions was constructed using Cytoscape. Throughout this study, gene sets were tested for enrichments of GO-Slim molecular functions and protein classes using PantherDB (Mi et al., 2013). See the Supplemental Information for further methodological details.

mRNA and Protein Abundance and Half-Lives of Parents

Protein and mRNA abundances were acquired from a microarray and shotgun proteomics study performed on the Daoy medulloblastoma cell line (Vogel et al., 2010), and protein half-life data were taken from a SILAC study in HeLa cells (Boisvert et al., 2012). These data sets were overlapped onto parent and non-parent gene sets, and differences in distributions of abundance and half-life by category were quantified by non-parametric Wilcoxon rank-sum tests.

Parent Gene Participation in Oncogenic Signaling Blocks

Disproportionate parent protein participation in cancer signaling processes (Cui et al., 2007) was assessed using a contingency table and a chi-square test of independence.

Parent Gene Essentiality

1,734 “core” essential genes shared between two cell lines (Blomen et al., 2015) were acquired and tested for enrichment among parent genes as above.

PPI Network Centrality

Network centrality calculations for both parent and non-parent genes/proteins were performed on a non-tissue specific PPI network (Wang et al., 2012) using the igraph R package. See the Supplemental Information for definitions of centrality measures. A tissue-specific PPI network (Bossi and Lehner, 2009) was acquired in order to calculate tissue-specific PPI metrics (Buljan et al., 2012). A more recent, expanded, and unbiased protein interaction data set from human cells (Huttlin et al., 2015) was also investigated.

Intrinsic Structural Disorder in Parent Proteins

Residue-by-residue predictions for disorder for each protein in the human proteome were generated using the IUPred program (Dosztányi et al., 2005; http://www.iupred.enzim.hu/). Scores range from 0 to 1, where higher scores indicate a higher propensity toward intrinsic disorder. Intrinsic disorder was calculated for genes (i.e., longest isoform Ensembl protein) and for specific included and excluded segments as an average over either the protein or segment length.

Analysis of Interacting Domains within Proteins

A data set of curated, structurally resolved PPIs was acquired (Meyer et al., 2013), and residues that form IMDs were mapped onto parent and non-parent proteins. IMD retention was quantified by calculating IMD residue densities on included and excluded segments. The frequency and completeness of retention of different domain types was summarized across the fusion protein set. Statistically significant differences between gene sets in the distributions of IMD residues were assessed as before. Parents which donate $\geq$20% of at least one IMD were analyzed for functional and protein class enrichments.

Identifying Novel and Retained PPIs of Fusion Proteins

The above set of domain-mediating PPIs was analyzed to identify which PPIs are recurrently (two or more times) retained in fusion proteins. DDIs were deemed to be “retained” if at least one fusion protein incorporated at least 90% of the IMD. Novel interactions created as a result of the transfer of IMDs were between protein A and B if there existed at least one fusion protein B-C, where C normally interacts with A and at least 90% of C’s IMD was retained. Novel links were those that did not appear in a set of known PPIs (Wang et al., 2012).

Identifying Shortest Path Distances between Proteins Newly Linked by Fusion

Pairwise shortest path lengths (geodesics) between all protein pairs in a PPI network (Wang et al., 2012) were calculated using igraph. The distribution of shortest path lengths in the novel link set was compared to the distribution of path lengths in 1,000 randomly sampled protein pairs from the complete geodesic matrix as before. Disconnected protein pairs had infinite shortest path lengths, reflecting the absence of a geodesic. A contingency table containing the counts of disconnected novel links versus other disconnected protein pairs was constructed and tested for independence using Fisher’s exact test.

Analysis of Interaction Interfaces in Parents

Structures of proteins in complex with proteins, DNA, or RNA molecules were obtained from the PDB and PISA database (http://www.ebi.ac.uk/pdbe/pisa/). Interface residues were identified and their positions converted into Ensembl protein coordinates. PISA residue densities were calculated by counting unique positions and dividing by protein lengths. Differences in the distributions of interface-forming PISA residue densities were analyzed as before. Biological process and protein class enrichments for parent genes that donate ten or more interface-forming residues to fusion proteins were calculated.

Analysis of Short Linear Peptide Motifs in Parents

A set of 1,410 experimentally validated (Dinkel et al., 2014) and 1,036,282 computationally predicted (Dosztányi et al., 2009) LMs were acquired and mapped onto proteins. LM densities were calculated by counting unique ELM accessions and dividing by protein length. Differences in LM density were assessed across parent gene sets and across included versus excluded segments. Due to the small sample size of experimentally verified LMs, functional enrichments were reported even if the number of genes in an enriched category was less than ten. Parent proteins that donate LMs to fusion proteins were assessed for functional enrichments.

Analysis of PTM Sites

PTM sites, which are candidate sites for regulating protein interactions, were acquired from the PTMcode v2 data set (Minguez et al., 2015). Differences in PTMcode site densities per gene were assessed for different parent gene sets and across included versus excluded segments. Further, we obtained and cleaned a data set of experimentally validated PTMs (dbPTM 3.0 database; Lu et al., 2013). PTM densities were analyzed as before at the whole protein and fusion segment level. Enrichments of specific types of modification sites were quantified in included and excluded segments.

Analysis of TF Fusions and the Expression Levels of Target Genes

Fusion transcripts in TCGA samples (Yoshihara et al., 2015) were filtered to identify fusions involving TFs (n = 1,131) (Table S7). The TCGA database (Tomczak et al., 2015) was queried to identify matched RNaseq data for TF fusion containing samples (n = 29). Normalized expression counts for each matched sample pair were extracted, genes with extremely small read counts (n < 10) removed, and DGE calculated as the absolute $\log_2$ fold change between the diseased and healthy samples. The regulated target genes of TFs were acquired from the TRRUST database (Han et al., 2015). DGE values for the TF targets were compared against all other genes using non-parametric Wilcoxon rank-sum tests in cases where sufficient regulatory targets ($n \geq 20$) were available (n = 8). The resulting p values were corrected for multiple testing using Holm's procedure.

SUPPLEMENTAL INFORMATION

Supplemental Information includes Supplemental Experimental Procedures, seven figures, and seven tables and can be found with this article online at http://dx.doi.org/10.1016/j.molcel.2016.07.008.

AUTHOR CONTRIBUTIONS

Study Conception and Design: N.S.L., R.J.W., and M.M.B.; Acquisition of Data: N.S.L., L.M., M.E.O., J.G., and R.J.W.; Analysis and Interpretation of Data: N.S.L., L.M., M.B., R.J.W., T.F., and M.M.B.; Manuscript Writing: N.S.L. and M.M.B.; and Critical Inputs to Manuscript: N.S.L., L.M., T.F., M.E.O., R.J.W., M.B., and M.M.B. The project was led by N.S.L. and supervised by M.M.B.

ACKNOWLEDGMENTS

We thank A. Krishnan, A. Bateman, B. Luisi, C. Ravarani, G. Chalancon, and S. Chavali for helpful discussions and feedback on the manuscript and M. Frenkel-Morgenstern for providing genomic coordinates from the ChiTaRS database for fusion protein mapping. This work was supported by the Medical Research Council (MC_U105185859 to M.M.B., N.S.L., L.M., R.J.W., and T.F and MC-A025-5PK11-6801 to M.B.), the Human Frontier Science Program (RGY0073/2010 to M.B. and M.M.B.), the Boehringer Ingelheim Fond (to T.F.), the Canadian Institute of Health Research (to R.J.W.), the IOF Marie Curie Fellowship (to R.J.W.), and the Lister Institute Research Prize Fellowship (to M.M.B.). We apologize for not being able to cite several relevant papers on this topic due to space constraints. We have extensively discussed a number of important papers in Latysheva and Babu (2016).

Received: April 12, 2016
Revised: June 14, 2016
Accepted: July 14, 2016
Published: August 18, 2016

REFERENCES

Abate, F., Zairis, S., Ficarra, E., Acquaviva, A., Wiggins, C.H., Frattini, V., Lasorella, A., Iavarone, A., Inghirami, G., and Rabadan, R. (2014). Pegasus: a comprehensive annotation and prediction tool for detection of driver gene fusions in cancer. BMC Syst. Biol. *8*, 97.

Blomen, V.A., Májek, P., Jae, L.T., Bigenzahn, J.W., Nieuwenhuis, J., Staring, J., Sacco, R., van Diemen, F.R., Olk, N., Stukalov, A., et al. (2015). Gene essentiality and synthetic lethality in haploid human cells. Science *350*, 1092–1096.

Boisvert, F.-M., Ahmad, Y., Gierliński, M., Charrière, F., Lamont, D., Scott, M., Barton, G., Lamond, A.I., Gierlinski, M., Charriere, F., et al. (2012). A quantitative spatial proteomics analysis of proteome turnover in human cells. Mol. Cell. Proteomics *11*, M111.011429.

Bondeson, D.P., Mares, A., Smith, I.E.D., Ko, E., Campos, S., Miah, A.H., Mulholland, K.E., Routly, N., Buckley, D.L., Gustafson, J.L., et al. (2015). Catalytic in vivo protein knockdown by small-molecule PROTACs. Nat. Chem. Biol. *11*, 611–617.

Bossi, A., and Lehner, B. (2009). Tissue specificity and the human protein interaction network. Mol. Syst. Biol. *5*, 260.

Buljan, M., Chalancon, G., Eustermann, S., Wagner, G.P., Fuxreiter, M., Bateman, A., and Babu, M.M. (2012). Tissue-specific splicing of disordered segments that embed binding motifs rewires protein interaction networks. Mol. Cell *46*, 871–883.

Charloteaux, B., Zhong, Q., Dreze, M., Cusick, M.E., Hill, D.E., and Vidal, M. (2011). Protein-protein interactions and networks: forward and reverse edgetics. Methods Mol. Biol. *759*, 197–213.

Cierpicki, T., and Grembecka, J. (2015). Targeting protein-protein interactions in hematologic malignancies: still a challenge or a great opportunity for future therapies? Immunol. Rev. *263*, 279–301.

Cui, Q., Ma, Y., Jaramillo, M., Bari, H., Awan, A., Yang, S., Zhang, S., Liu, L., Lu, M., O'Connor-McCourt, M., et al. (2007). A map of human cancer signaling. Mol. Syst. Biol. *3*, 152.

Davey, N.E., Van Roey, K., Weatheritt, R.J., Toedt, G., Uyar, B., Altenberg, B., Budd, A., Diella, F., Dinkel, H., and Gibson, T.J. (2012). Attributes of short linear motifs. Mol. Biosyst. *8*, 268–281.

David, A., Razali, R., Wass, M.N., and Sternberg, M.J.E. (2012). Protein-protein interaction sites are hot spots for disease-associated nonsynonymous SNPs. Hum. Mutat. *33*, 359–363.

Dinkel, H., Van Roey, K., Michael, S., Davey, N.E., Weatheritt, R.J., Born, D., Speck, T., Krüger, D., Grebnev, G., Kuban, M., et al. (2014). The eukaryotic linear motif resource ELM: 10 years and counting. Nucleic Acids Res. *42*, D259–D266.

Dosztányi, Z., Csizmok, V., Tompa, P., and Simon, I. (2005). IUPred: web server for the prediction of intrinsically unstructured regions of proteins based on estimated energy content. Bioinformatics *21*, 3433–3434.

Dosztányi, Z., Mészáros, B., and Simon, I. (2009). ANCHOR: web server for predicting protein binding regions in disordered proteins. Bioinformatics *25*, 2745–2746.

Frenkel-Morgenstern, M., Gorohovski, A., Lacroix, V., Rogers, M., Ibanez, K., Boullosa, C., Andres Leon, E., Ben-Hur, A., and Valencia, A. (2013). ChiTaRS: a database of human, mouse and fruit fly chimeric transcripts and RNA-sequencing data. Nucleic Acids Res. *41*, D142–D151.

Gianni, M., Boldetti, A., Guarnaccia, V., Rambaldi, A., Parrella, E., Raska, I., Jr., Rochette-Egly, C., Del Sal, G., Rustighi, A., Terao, M., and Garattini, E. (2009). Inhibition of the peptidyl-prolyl-isomerase Pin1 enhances the responses of acute myeloid leukemia cells to retinoic acid via stabilization of RARalpha and PML-RARalpha. Cancer Res. *69*, 1016–1026.

Han, H., Shim, H., Shin, D., Shim, J.E., Ko, Y., Shin, J., Kim, H., Cho, A., Kim, E., Lee, T., et al. (2015). TRRUST: a reference database of human transcriptional regulatory interactions. Sci. Rep. *5*, 11432.

Hegyi, H., Buday, L., and Tompa, P. (2009). Intrinsic structural disorder confers cellular viability on oncogenic fusion proteins. PLoS Comput. Biol. *5*, e1000552.

Höglund, M., Frigyesi, A., and Mitelman, F. (2006). A gene fusion network in human neoplasia. Oncogene *25*, 2674–2678.

Huang, F., and Chen, Y.-G. (2012). Regulation of TGF-β receptor activity. Cell Biosci. *2*, 9.

Huttlin, E.L., Ting, L., Bruckner, R.J., Gebreab, F., Gygi, M.P., Szpyt, J., Tam, S., Zarraga, G., Colby, G., Baltier, K., et al. (2015). The BioPlex Network: A systematic exploration of the human interactome. Cell *162*, 425–440.

Jin, L., Wang, W., and Fang, G. (2014). Targeting protein-protein interaction by small molecules. Annu. Rev. Pharmacol. Toxicol. *54*, 435–456.

Jividen, K., and Li, H. (2014). Chimeric RNAs generated by intergenic splicing in normal and cancer cells. Genes Chromosomes Cancer *53*, 963–971.

Kuenemann, M.A., Sperandio, O., Labbé, C.M., Lagorce, D., Miteva, M.A., and Villoutreix, B.O. (2015). In silico design of low molecular weight protein-protein interaction inhibitors: Overall concept and recent advances. Prog. Biophys. Mol. Biol. *119*, 20–32.

Kumar-Sinha, C., Kalyana-Sundaram, S., and Chinnaiyan, A.M. (2015). Landscape of gene fusions in epithelial cancers: seq and ye shall find. Genome Med. *7*, 129.

Latysheva, N.S., and Babu, M.M. (2016). Discovering and understanding oncogenic gene fusions through data intensive computational approaches. Nucleic Acids Res. *44*, 4487–4503.

Lobato, M.N., Metzler, M., Drynan, L., Forster, A., Pannell, R., and Rabbitts, T.H. (2008). Modeling chromosomal translocations using conditional alleles to recapitulate initiating events in human leukemias. J. Natl. Cancer Inst. Monogr. *39*, 58–63.

Lu, C.-T., Huang, K.-Y., Su, M.-G., Lee, T.-Y., Bretaña, N.A., Chang, W.-C., Chen, Y.-J., Chen, Y.-J., and Huang, H.-D. (2013). DbPTM 3.0: an informative resource for investigating substrate site specificity and functional association of protein post-translational modifications. Nucleic Acids Res. *41*, D295–D305.

Mertens, F., Johansson, B., Fioretos, T., and Mitelman, F. (2015a). The emerging complexity of gene fusions in cancer. Nat. Rev. Cancer *15*, 371–381.

Mertens, F., Antonescu, C.R., and Mitelman, F. (2015b). Gene fusions in soft tissue tumors: recurrent and overlapping pathogenetic themes. Genes Chromosomes Cancer *55*, 291–310.

Meyer, M.J., Das, J., Wang, X., and Yu, H. (2013). INstruct: a database of high-quality 3D structurally resolved protein interactome networks. Bioinformatics *29*, 1577–1579.

Mi, H., Muruganujan, A., Casagrande, J.T., and Thomas, P.D. (2013). Large-scale gene function analysis with the PANTHER classification system. Nat. Protoc. *8*, 1551–1566.

Minguez, P., Letunic, I., Parca, L., Garcia-Alonso, L., Dopazo, J., Huerta-Cepas, J., and Bork, P. (2015). PTMcode v2: a resource for functional associations of post-translational modifications within and between proteins. Nucleic Acids Res. *43*, D494–D502.

Mitelman, F., Johansson, B., and Mertens, F. (2007). The impact of translocations and gene fusions on cancer causation. Nat. Rev. Cancer *7*, 233–245.

Nam, R.K., Sugar, L., Yang, W., Srivastava, S., Klotz, L.H., Yang, L.-Y., Stanimirovic, A., Encioiu, E., Neill, M., Loblaw, D.A., et al. (2007). Expression of the TMPRSS2:ERG fusion gene predicts cancer recurrence after surgery for localised prostate cancer. Br. J. Cancer *97*, 1690–1695.

Oates, M.E., Romero, P., Ishida, T., Ghalwash, M., Mizianty, M.J., Xue, B., Dosztányi, Z., Uversky, V.N., Obradovic, Z., Kurgan, L., et al. (2013). D^2P^2: database of disordered protein predictions. Nucleic Acids Res. *41*, D508–D516.

Pawson, T., and Warner, N. (2007). Oncogenic re-wiring of cellular signaling pathways. Oncogene *26*, 1268–1275.

Robinson, D.R., Kalyana-Sundaram, S., Wu, Y.-M., Shankar, S., Cao, X., Ateeq, B., Asangani, I.A., Iyer, M., Maher, C.A., Grasso, C.S., et al. (2011). Functionally recurrent rearrangements of the MAST kinase and Notch gene families in breast cancer. Nat. Med. *17*, 1646–1651.

Rolland, T., Taşan, M., Charloteaux, B., Pevzner, S.J., Zhong, Q., Sahni, N., Yi, S., Lemmens, I., Fontanillo, C., Mosca, R., et al. (2014). A proteome-scale map of the human interactome network. Cell *159*, 1212–1226.

Shugay, M., Ortiz de Mendíbil, I., Vizmanos, J.L., and Novo, F.J. (2013). Oncofuse: a computational framework for the prediction of the oncogenic potential of gene fusions. Bioinformatics *29*, 2539–2546.

Sudha, G., Nussinov, R., and Srinivasan, N. (2014). An overview of recent advances in structural bioinformatics of protein-protein interactions and a guide to their principles. Prog. Biophys. Mol. Biol. *116*, 141–150.

Tognon, C.E., Somasiri, A.M., Evdokimova, V.E., Trigo, G., Uy, E.E., Melnyk, N., Carboni, J.M., Gottardis, M.M., Roskelley, C.D., Pollak, M., and Sorensen, P.H. (2011). ETV6-NTRK3-mediated breast epithelial cell transformation is blocked by targeting the IGF1R signaling pathway. Cancer Res. *71*, 1060–1070.

Tomczak, K., Czerwińska, P., and Wiznerowicz, M. (2015). The Cancer Genome Atlas (TCGA): an immeasurable source of knowledge. Contemp. Oncol. (Pozn.) *19* (1A), A68–A77.

Tompa, P., Davey, N.E., Gibson, T.J., and Babu, M.M. (2014). A million peptide motifs for the molecular biologist. Mol. Cell *55*, 161–169.

Van Roey, K., Dinkel, H., Weatheritt, R.J., Gibson, T.J., and Davey, N.E. (2013). The switches.ELM resource: a compendium of conditional regulatory interaction interfaces. Sci. Signal. *6*, rs7.

Varley, K.E., Gertz, J., Roberts, B.S., Davis, N.S., Bowling, K.M., Kirby, M.K., Nesmith, A.S., Oliver, P.G., Grizzle, W.E., Forero, A., et al. (2014). Recurrent read-through fusion transcripts in breast cancer. Breast Cancer Res. Treat. *146*, 287–297.

Vitari, A.C., Leong, K.G., Newton, K., Yee, C., O'Rourke, K., Liu, J., Phu, L., Vij, R., Ferrando, R., Couto, S.S., et al. (2011). COP1 is a tumour suppressor that causes degradation of ETS transcription factors. Nature *474*, 403–406.

Vogel, C., Abreu, R. de S., Ko, D., Le, S.-Y., Shapiro, B.A., Burns, S.C., Sandhu, D., Boutz, D.R., Marcotte, E.M., and Penalva, L.O. (2010). Sequence signatures and mRNA concentration can explain two-thirds of protein abundance variation in a human cell line. Mol. Syst. Biol. *6*, 400.

Wang, X., Gulbahce, N., and Yu, H. (2011). Network-based methods for human disease gene prediction. Brief. Funct. Genomics *10*, 280–293.

Wang, X., Wei, X., Thijssen, B., Das, J., Lipkin, S.M., and Yu, H. (2012). Three-dimensional reconstruction of protein networks provides insight into human genetic disease. Nat. Biotechnol. *30*, 159–164.

Wang, Y., Sahni, N., and Vidal, M. (2015). Global edgetic rewiring in cancer networks. Cell Syst. *1*, 251–253.

Watson, I.R., Takahashi, K., Futreal, P.A., and Chin, L. (2013). Emerging patterns of somatic mutations in cancer. Nat. Rev. Genet. *14*, 703–718.

Winter, G.E., Buckley, D.L., Paulk, J., Roberts, J.M., Souza, A., Dhe-Paganon, S., and Bradner, J.E. (2015). Drug Development. Phthalimide conjugation as a strategy for in vivo target protein degradation. Science *348*, 1376–1381.

Wu, G., Feng, X., and Stein, L. (2010). A human functional protein interaction network and its application to cancer data analysis. Genome Biol. *11*, R53.

Yoshihara, K., Wang, Q., Torres-Garcia, W., Zheng, S., Vegesna, R., Kim, H., and Verhaak, R.G.W. (2015). The landscape and therapeutic relevance of cancer-associated transcript fusions. Oncogene *34*, 4845–4854.

Yu, C.-Y., Liu, H.-J., Hung, L.-Y., Kuo, H.-C., and Chuang, T.-J. (2014). Is an observed non-co-linear RNA product spliced in trans, in cis or just in vitro? Nucleic Acids Res. *42*, 9410–9423.

Zhang, Y., Gong, M., Yuan, H., Park, H.G., Frierson, H.F., and Li, H. (2012). Chimeric transcript generated by cis-splicing of adjacent genes regulates prostate cancer cell proliferation. Cancer Discov. *2*, 598–607.

Molecular Cell, Volume *63*

Supplemental Information

Molecular Principles of Gene Fusion Mediated Rewiring of Protein Interaction Networks in Cancer

Natasha S. Latysheva, Matt E. Oates, Louis Maddox, Tilman Flock, Julian Gough, Marija Buljan, Robert J. Weatheritt, and M. Madan Babu

Latysheva, Fig. S1 (related to Fig. 1)

A Gene fusion network
(zoomable svg format)

Nodes = genes
Edges = presence of a gene fusion between the two genes

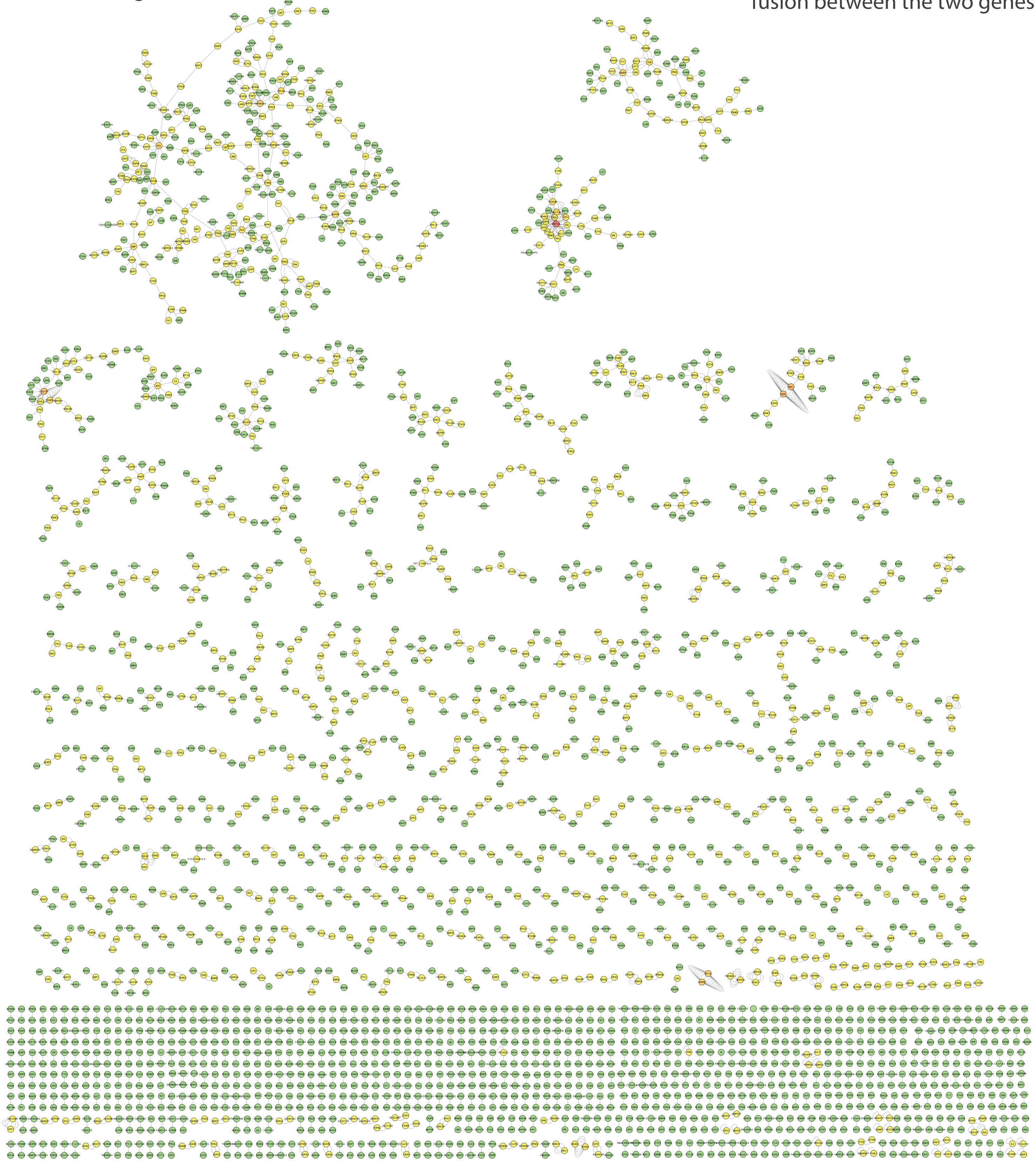

B Distribution of degree

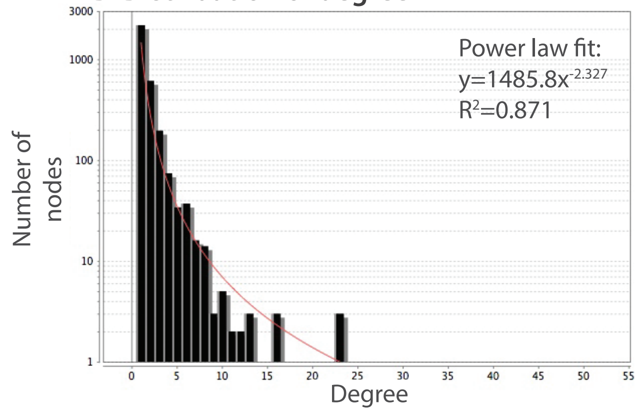

Node colour:

1 Degree 53

Figure S1. Network of gene fusions (related to Figure 1). (A) Nodes indicate genes and edges indicate the occurrence of a fusion between genes. Node colour indicates the degree of each node, or the count of distinct genes the node/gene fuses with. **(B)** Distribution of degree in the gene fusion network with a fitted power law curve. Note that one instance of fusion degree greater than 23 exists (KTM2A degree=53) but is excluded from the degree chart due to logarithmic scales. Oncogenes (OGs) and tumour suppressor genes (TSGs) are overrepresented in the parent set (OG: χ^2=62.1, df=1, p=3.3e^{-15}; TSG: χ^2=42.1, df=1, p=8.5e^{-11}). Over a third of known OGs (78/216) and a quarter of known TSGs (161/626) form gene fusions ($n_{OG\ fusions}$=184; $n_{TSG\ fusions}$=291) within this dataset. There exist instances of OG-TSG (n=38), OG-OG (n=14) and TSG-TSG (n=11) fusions.

Latysheva, Fig. S2 (related to Fig. 1)

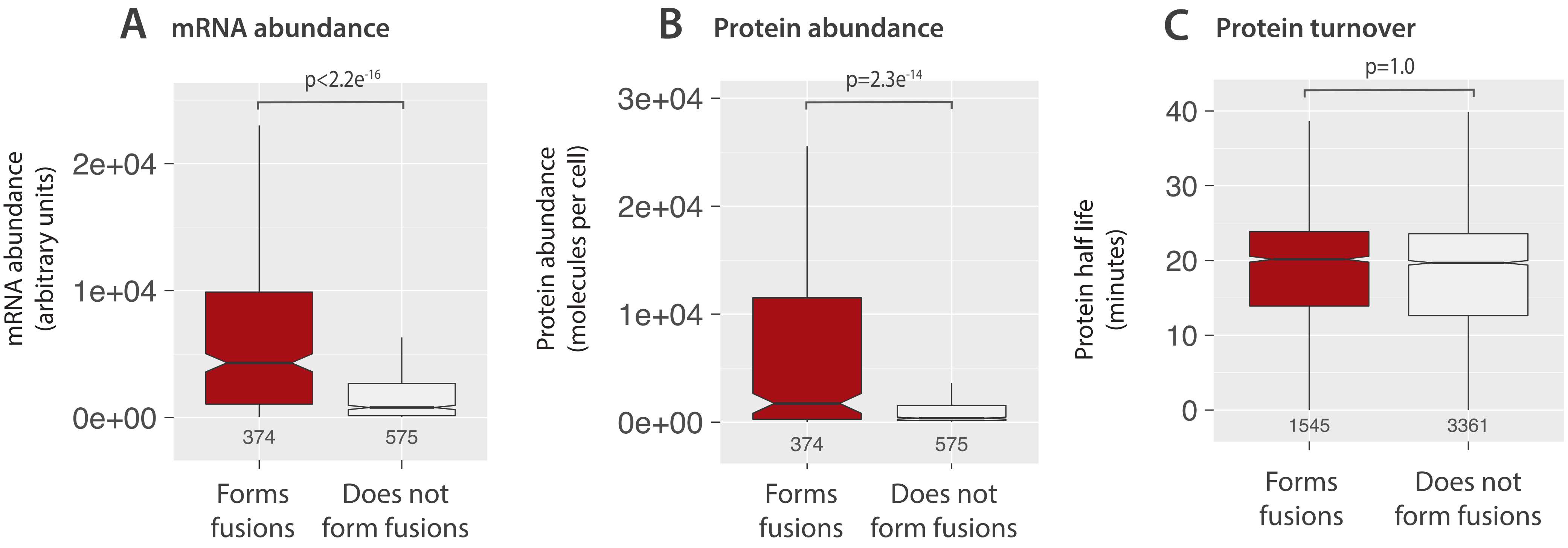

D Oncogenic signaling genes

	Oncogenic signaling gene	Not oncogenic signaling gene
Parent gene	57	3281
Non-parent gene	124	16941

χ^2=29.5, df=1, p=5.7e-08

E Essentiality of parent genes

	Essential gene	Not essential gene
Parent gene	576	2762
Non-parent gene	1151	15914

χ^2=396.8, df=1, p<2.2e-16

Figure S2. mRNA abundance, protein abundance, protein half-lives and gene identities in parents (related to Figure 1). (A) mRNA and **(B)** protein abundances of parent and non-parent genes from the Daoy medulloblastoma cell line. **(C)** Protein half-lives of parent and non-parent genes from SILAC experiments in HeLa cells. Enrichment of parent genes for **(D)** cancer signaling genes and **(E)** genes essential for cellular viability.

Latysheva, Fig. S3 (related to Fig. 2)

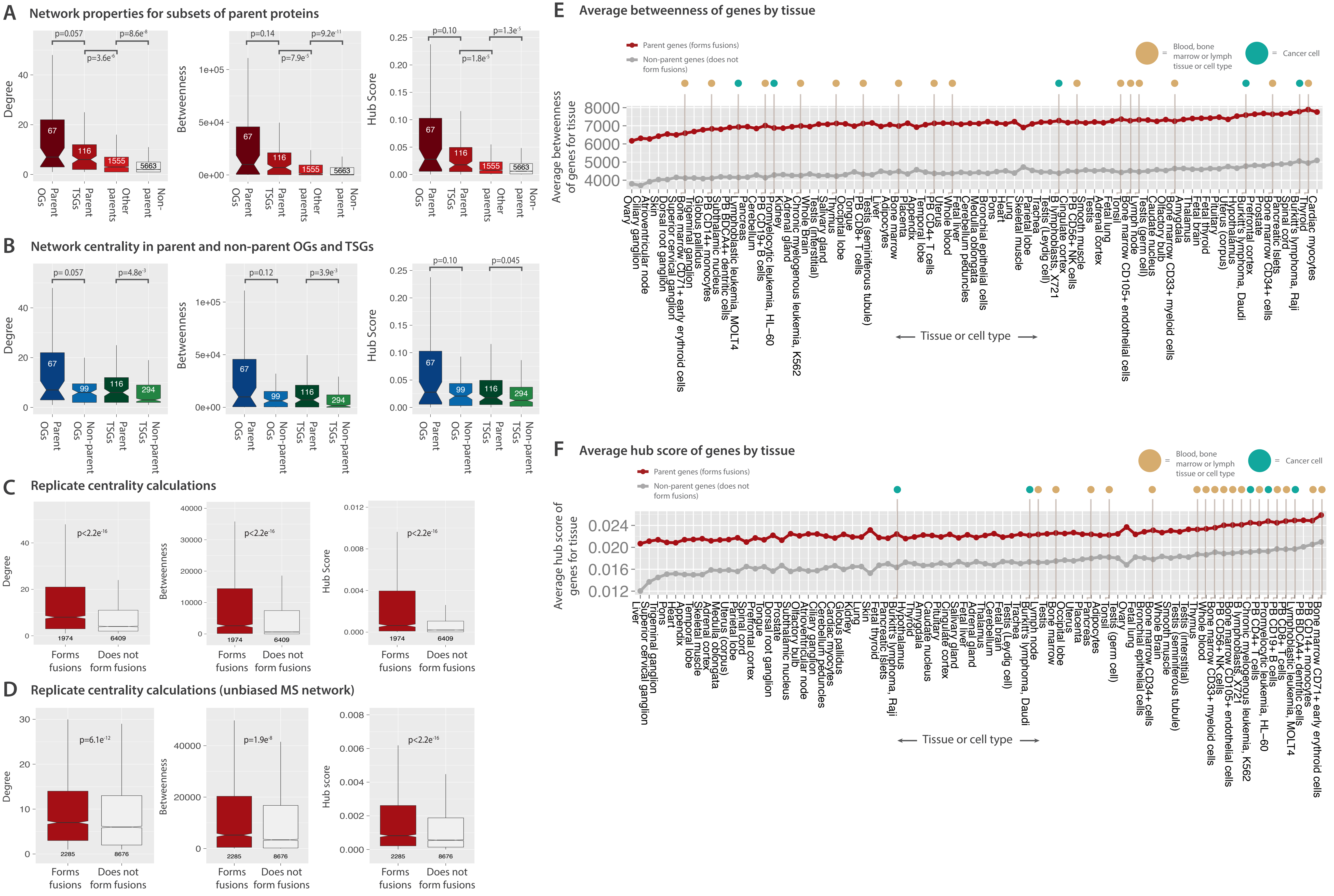

Figure S3. Network centrality support calculations (related to Figure 2). (A) Network centrality scores for parent gene sets and non-parents. **(B)** Network centrality scores for parent and non-parent oncogenes and tumour suppressor genes. **(C)** Replicate network centrality calculations for parent and non-parent genes on an additional PPI dataset. **(D)** Replicate network centrality calculations for parent and non-parent genes on an unbiased PPI dataset based on mass spectrometry data. Average tissue-specific betweenness centrality **(E)** and hub score centrality **(F)** of parent genes.

Latysheva, Fig. S4 (related to Fig. 2)

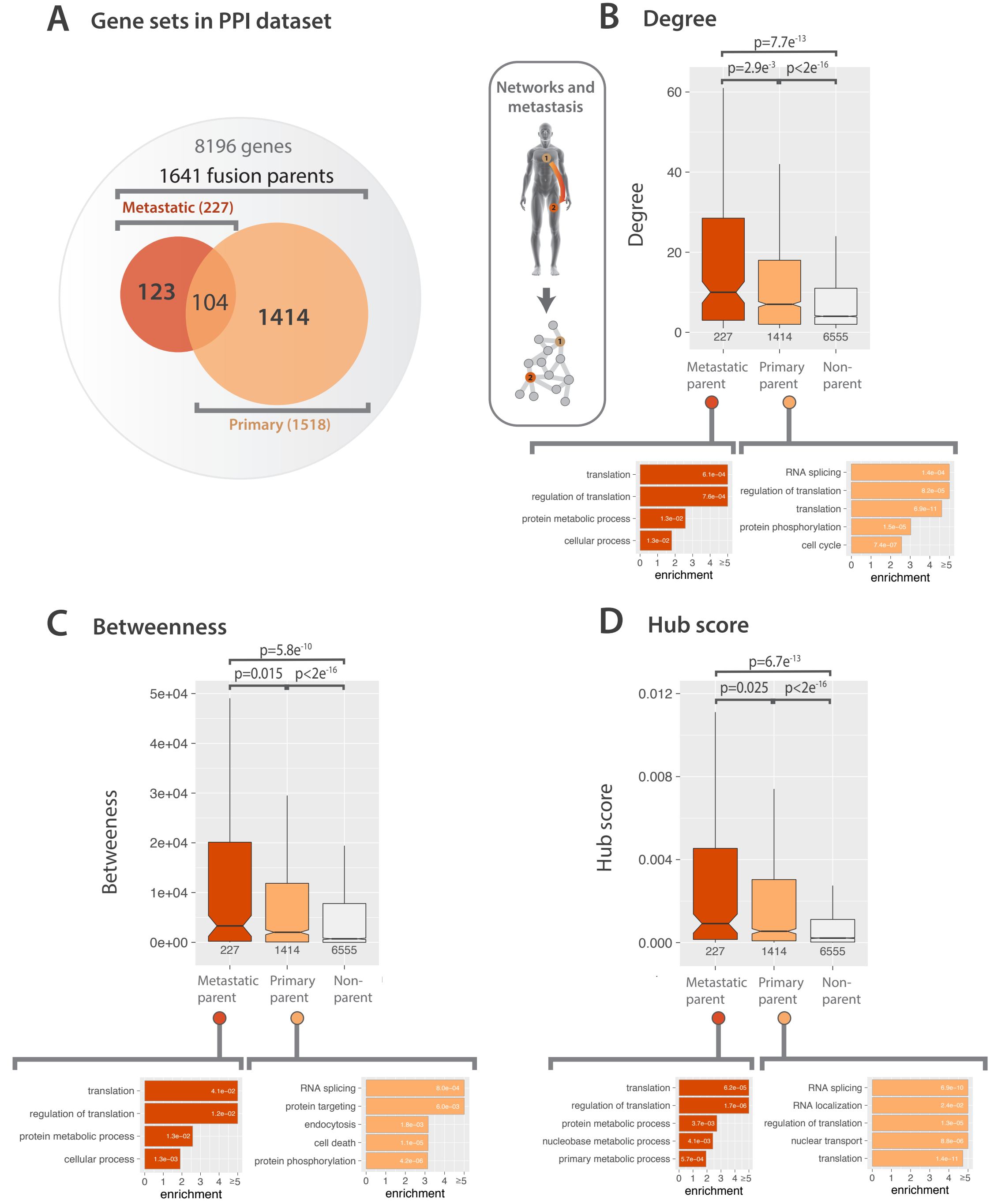

Figure S4. Network centrality in metastatic and primary tumour parent genes (related to Figure 2). Parent genes were labeled as "metastatic" if the gene formed a fusion that was detected in at least 1 cell line of metastatic tumour origin and "primary" if the gene only formed fusions detected in cell lines derived from primary tumours. **(A)** Counts of metastatic tumour parent genes, primary tumour parent genes, and non-parent genes within the PPI dataset. The degree **(B)**, betweenness centrality **(C)** and hub score **(D)** distributions by gene class. Bar plots show gene ontology enrichments in metastatic fusion genes in the top quartile of centrality values of the class. Although we find that parent genes from metastatic tumours have borderline significantly higher degree, betweenness and hub scores compared to parent genes from primary tumours, an alternative PPI dataset derived from MS data (Huttlin et al., 2015) does not replicate this trend (data not shown). There is an indication that the top quartile of metastatic and primary parents may have distinct functions (see also **Table S4**) - however, terms classically associated with metastasis (e.g. motility, tissue invasion, anoikis) were not found to be enriched.

Latysheva, Fig. S5 (related to Fig. 3)

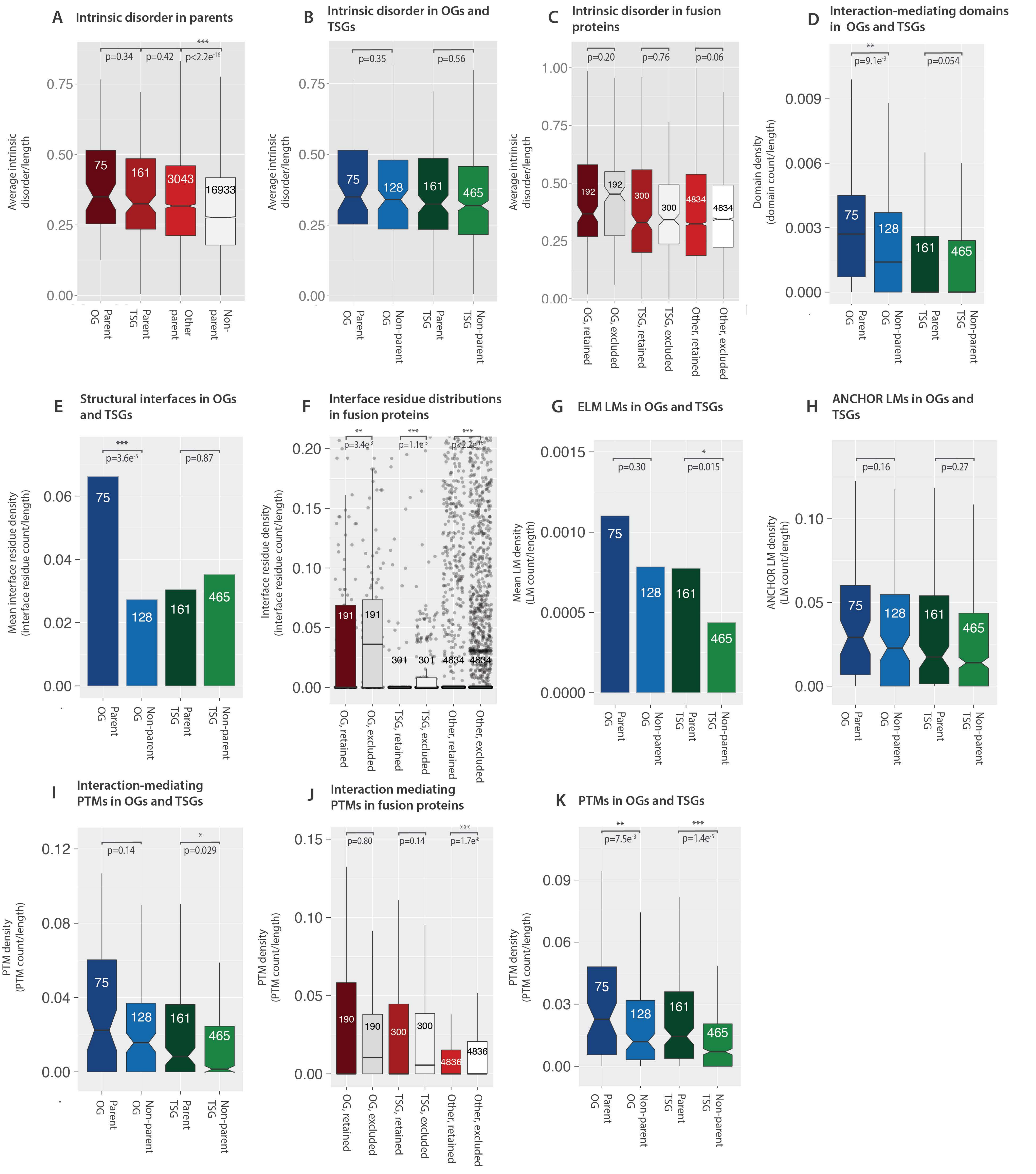

Figure S5. Molecular features of fusion proteins (related to Figure 3). (A) Average intrinsic disorder by gene for OG parents, TSG parents, other parents and non-parents. **(B)** Average intrinsic disorder in parent OGs and TSGs versus non-parent OGs and TSGs. **(C)** Average intrinsic disorder in included and excluded fusion segments by gene category. **(D)** Densities of interaction-mediating domains in parent versus non-parent OGs and TSGs. **(E)** Structural interface residue densities in parent versus non-parent OGs and TSGs. **(F)** Structural interface residue densities in included and excluded fusion segments by gene category. Plot data is identical to Figure 3D but with detailed distributions displayed instead of means. (**G**) Mean ELM linear motif densities in parent versus non-parent OGs and TSGs. (**H**) ANCHOR linear motif densities in parent versus non-parent OGs and TSGs. **(I)** Interaction-mediating PTM densities in parent versus non-parent OGs and TSGs. **(J)** Interaction-mediating PTM densities in included and excluded fusion segments by gene category. **(K)** PTM densities in parent versus non-parent OGs and TSGs.

Latysheva, Fig. S6 (related to Fig. 4)

A Retained domain-domain interactions in fusion proteins resulting from domain transfer

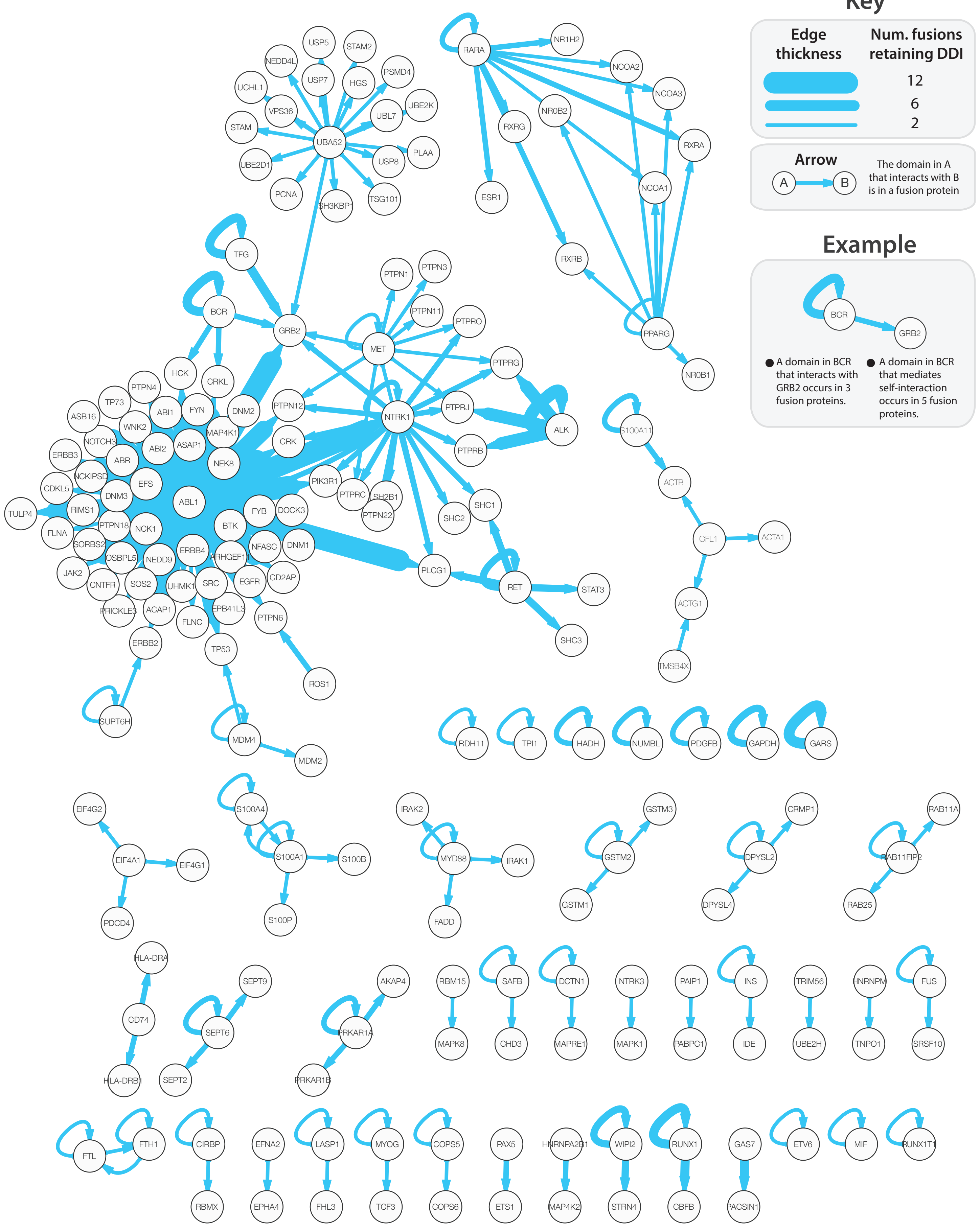

B Novel protein-protein interactions resulting from domain transfer

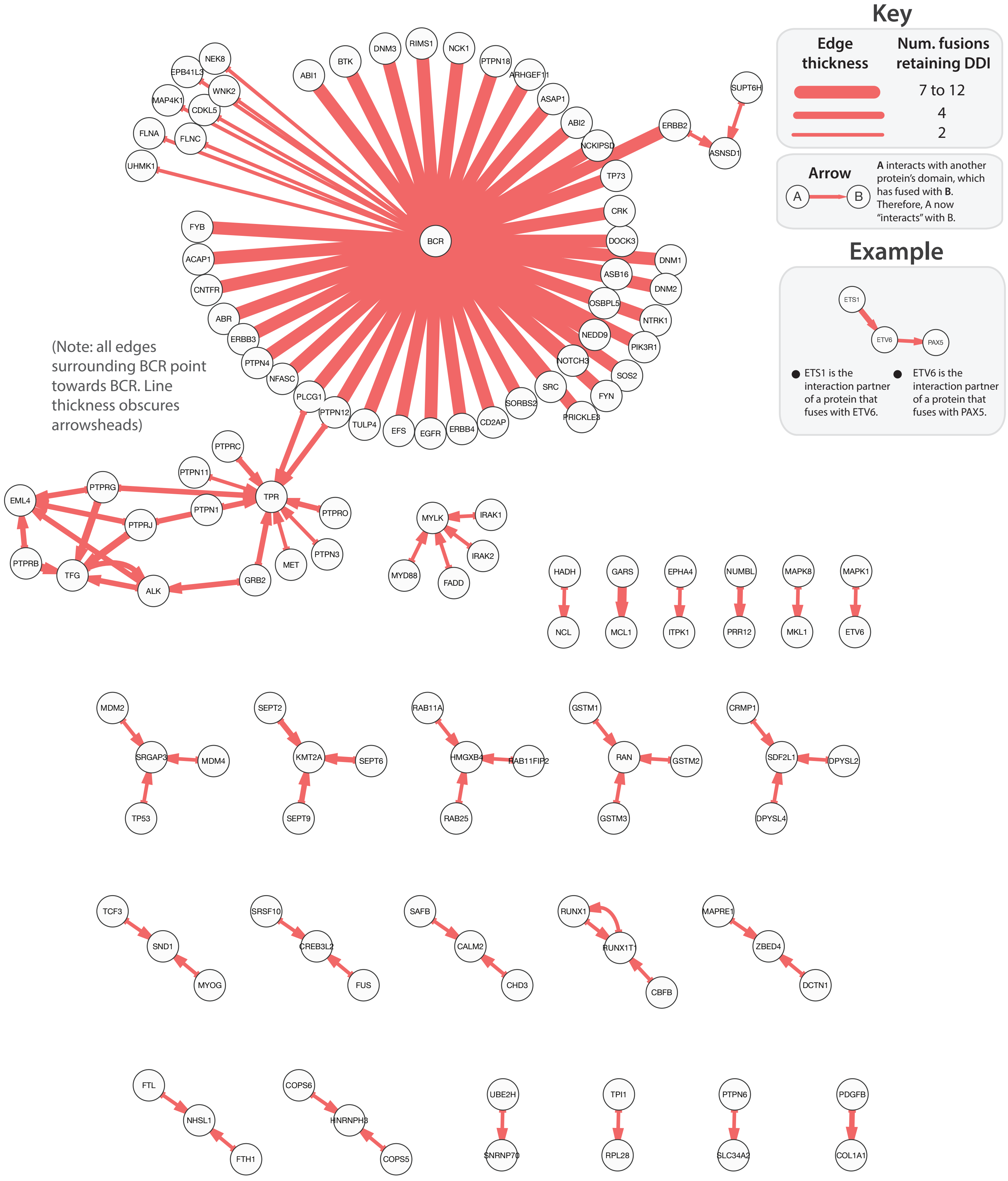

C Shortest path length distributions

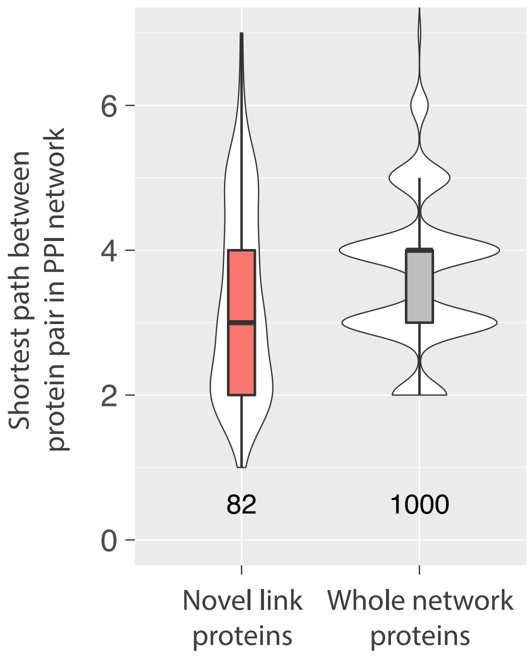

D Disconnected protein pairs

	No connecting path exists	Connecting path exists
Novel link protein pairs	34	82
All other protein pairs	5695274	47638419

Fisher's exact test, odds ratio=3.468, $p=2.96e^{-08}$

E Protein pairs with no previous connecting path which are linked by fusion

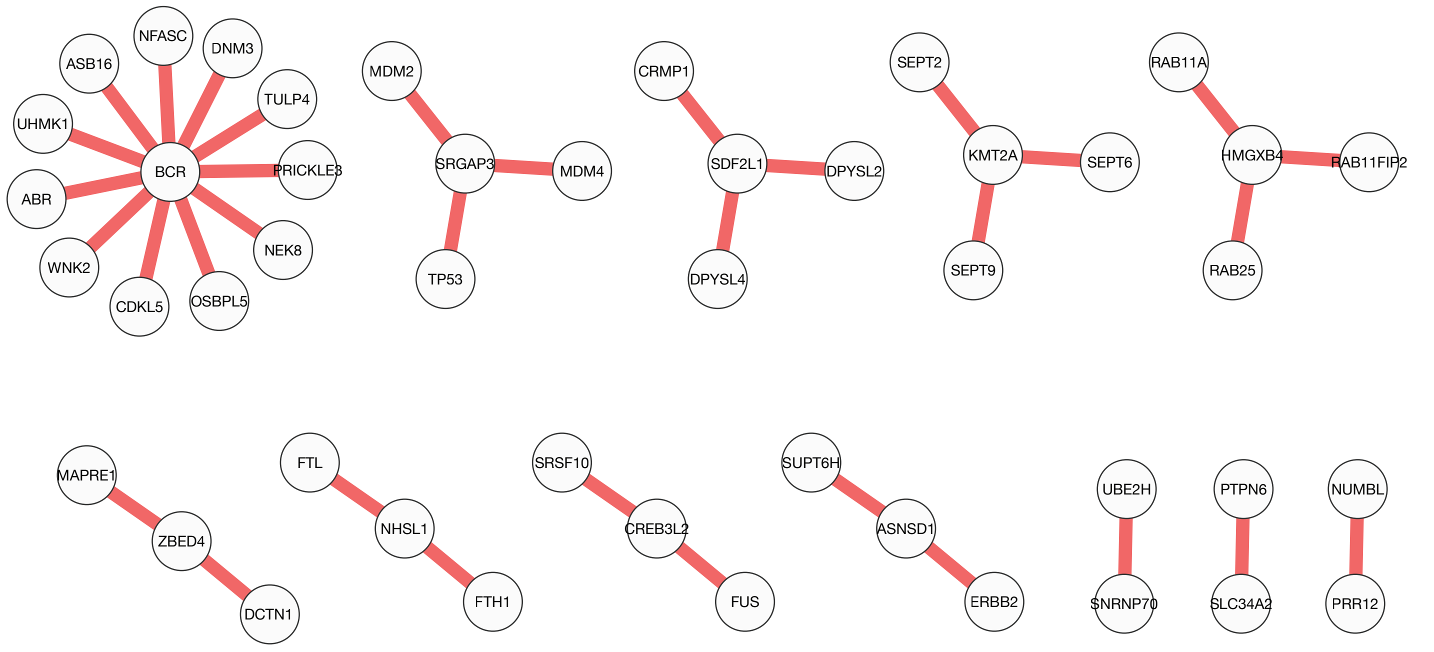

Figure S6. Retained and novel protein-protein interactions caused by fusion events (related to Figure 4). **(A)** Protein-protein interactions which are recurrently retained within fusion proteins as a result of the repeated inclusion of large portions (≥90% of residues) of interaction-mediating domains (IMDs). The most frequently conserved domain-domain interactions in fusion proteins include: the numerous interactions of ABL1, ALK interactions with ALK and three receptor-type protein tyrosine phosphatases (PTPRB, PTPRG, PTPRJ); GARS-GARS interaction; BCR interactions with BCR, CRKL, GRB2, HCK; and several RET interactions. The conserved domain-domain interactions can repeat - in certain cases, several different fusions conserve the same domain-domain interactions. For example, fusion proteins incorporating NTRK1, MET, RET, ALK, and BCR recurrently retain domains which mediate similar interactions. **(B)** Novel protein-protein links which arise from the recurrent transfer of large portions (≥90% of residues) of interaction-mediating domains (IMDs). A protein interaction link was drawn between proteins A and B if there existed some fusion protein B-C, where C normally interacts with A and at least 90% of C's interaction mediating domain was retained. Previously known PPIs were filtered out. We use "link" instead of "interaction" since these proteins do not necessarily interact – they are simply brought into proximity of each other, which may or may not result in interaction. **(C)** Fusion-mediated network rewiring of disparate areas of the native protein interaction network. Comparison of the shortest path distributions between proteins newly linked by fusion events and a set of 1000 random protein pairs from the interaction network. **(D)** Proportions of protein pairs with no available shortest path in the novel link set and in the whole network. The shortest paths between novel links were in fact slightly shorter than in other protein pairs in the network (on average, 3.28 compared to 3.71, respectively; W=32444, $p=1.0e^{-3}$). However, whereas only 10.7% of protein pairs in the PPI network had no connecting path, 29.3% of protein pairs in the novel links had no connecting path, indicating that the novel link set of protein pairs is disproportionately composed of protein pairs which previously had no existing path between them within the normal network. This reflects a strong enrichment for fusion proteins to interconnect protein sets which previously resided in completely separate sections of the interactome (Fisher's exact test on contingency table, odds ratio=3.47, $p=3.0e^{-8}$). **(E)** Newly linked protein pairs with no previous shortest path between them. For these proteins, fusion changes the previously infinite shortest path distances to a distance of 1. KTM2A (MLL), fusions of which are associated with aggressive leukaemia, is now connected to three members of the septin family (SEPT2, SEPT6, SEPT9), which are cytoskeletal components implicated in cancer development. Further, SRGAP3, a Rho GTPase activating protein, is now brought into proximity with MDM2, MDM4, and TP53, which are crucial regulators of cancer development and progression. Interestingly, MDM2 and MDM4 are known negative regulators of p53. Furthermore, BCR gains 11 novel links, which include proteins with roles in protein degradation and cell cycle progression.

Latysheva, Fig. S7 (related to Fig. 6)

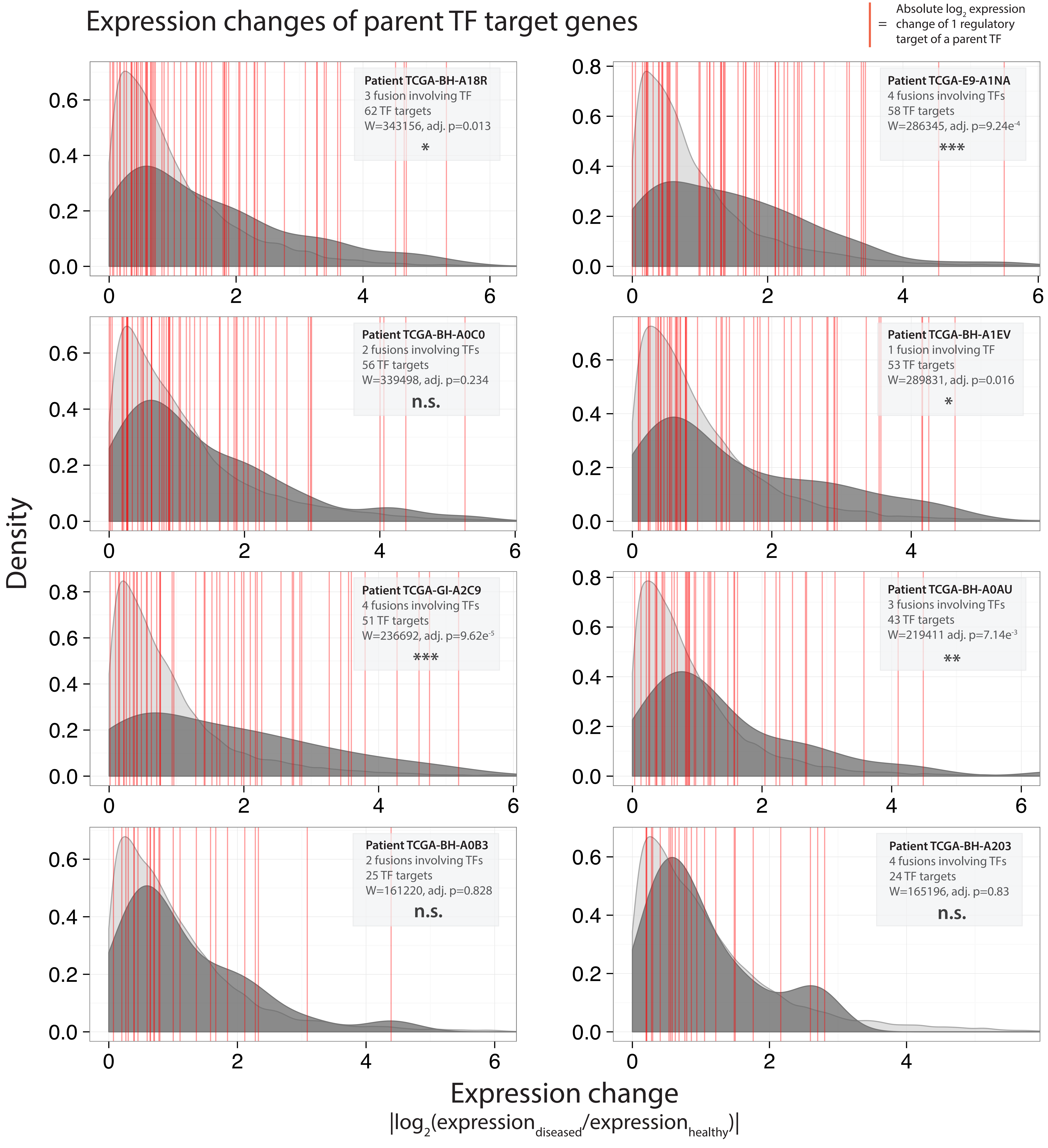

Figure S7. Fusion-mediated deregulation of transcription factor target genes in breast cancer (related to Figure 6). In fusions affecting transcription factor proteins, the downstream rewiring effects of the fusion may be investigated by analysing expression changes in the target genes of the transcription factor parent. Plots display comparisons of the differential gene expression (DGE) values of all genes (light grey distributions) in 8 breast cancer samples against DGE values of the regulatory targets (red vertical lines and dark grey distributions) of transcription factors forming the fusion transcripts within samples.

SUPPLEMENTAL TABLE LEGENDS

Table S1. Fusion proteins and parent functions (related to Figure 1). Description of fusion proteins used in this study and biological process and protein class enrichments of parent genes. Gene symbols in the original ChiTaRS mapping can differ from gene names associated with mapped Ensembl proteins (see **Supplemental Experimental Procedures**), and we provide both gene name sets in the fusion protein listing (see the fusion protein mapping web server http://fusion.d2p2.pro/ for further details).

Table S2. Functions of the top quartile centrality genes (related to Figure 2). Biological process and protein class enrichments of parent and non-parent genes with the highest PPI network centralities.

Table S3. Tissue specific network centrality (related to Figure 2). Averaged network centrality measures for parent and non-parent proteins in tissue-specific interaction networks.

Table S4. Interaction-mediating domains in fusion proteins and parent functions (related to Figure 3). Interaction-mediating domain (IMD) residues incorporated into fusion proteins and biological process and protein class enrichments of parent genes which donate ≥20% of an IMD.

Table S5. Retained and novel protein-protein interactions arising from fusion-mediated domain recombination (related to Figure 4). Retained and novel protein-protein interactions resulting from the transfer of largely intact (≥90% of the domain sequence) interaction-mediating domains into fusion proteins.

Table S6. Interfaces, linear motifs, and PTMs in fusion proteins and parent functions (related to Figure 3). Structural interfaces of protein complexes which are incorporated into fusion proteins and biological process and protein class enrichments of parent genes which donate 10 or more interface-forming residues; Experimentally validated short linear peptide motifs incorporated into fusion proteins and biological process and protein class enrichments of parent genes which donate at least one such linear motif; Experimentally validated post-translational modifications incorporated into fusion proteins. Biological process and protein class enrichments for parent genes which either retain or lose ≥90% of their PTM content upon fusion. Certain PTM types were found to occur in both included and excluded segments more frequently than expected given the global frequencies of all known PTMs, such as S-Nitrosylation (1.7x enrichment in included segments, 1.6x in excluded segments). Other PTM types showed differential presence/absence patterns based on segment inclusion: methylation sites are more highly enriched in included segments (3.5x enrichment) than in excluded segments (2.5x), as are acetylation sites (1.8x included, 1.3x excluded). Interestingly, both N-linked and O-linked glycosylation, which are involved in protein folding and stability and cancer processes like migration and invasion, are generally depleted in parent proteins.

Table S7. Fusion-mediated deregulation of transcription factor target genes (related to Figure 6). Differential expression analysis of breast cancer samples containing fusion transcripts composed of at least 1 transcription factor (TF) parent. Differential gene expression values of the TF targets were compared to those of all other genes.

SUPPLEMENTAL EXPERIMENTAL PROCEDURES

To compare properties of parent genes and proteins against non-parents, all human gene and proteins and their sequences were first acquired using Ensembl's ftp page (http://www.ensembl.org/info/data/ftp/index.html). Where possible, parent versus non-parent comparisons were performed at the gene level to avoid systematic biases for or against proteins with multiple isoforms. Unless otherwise specified, calculations of structural features for genes were obtained by considering the longest protein isoform. For network and structural analyses, parent genes were categorized according to whether they were known oncogenes or tumour suppressor genes. Throughout this study, the biological function of gene sets was assessed using statistical overrepresentation tests on PANTHER GO-Slim biological process and PANTHER protein class annotations, and unless stated otherwise, functional enrichments were only reported in the main text if 10 or more genes were present in a category.

For evaluating statistical significance of the differences in distributions between gene sets, non-parametric Wilcoxon rank sum tests were performed using R. In plots comparing more than two distributions against each other (e.g. Figure 3A, 3C), Holm's correction for multiple testing was employed. Following plotting convention, 1 star ('*') indicates p≤0.05, 2 stars ('**') indicates p≤0.01, and 3 stars ('***') indicates p≤0.001. All data integration and analysis was performed using custom R, Python and bash scripts and SQL queries. Visualization was done using the ggplot2 package in R, Cytoscape, and Adobe Illustrator. The webserver displaying fusion protein mappings (http://fusion.d2p2.pro) was generated via integration with the D^2P^2 resource (Oates et al. 2013).

Database identification, processing and integration

To compose a set of human fusion proteins, we acquired a database (ChiTaRS database v1; Frenkel-Morgenstern et al. 2013) of 9,237 fusion mRNAs, which represented the largest collection of human fusion sequences until the release of ChiTaRS v2. ChiTaRS is derived from a large scale analysis of EST and RNA-seq data from GenBank (Benson et al., 2012). GenBank obtains submissions primarily from individual laboratories and large-scale sequencing projects. The ChiTaRS project labeled entries as 'chimeric' if mRNA sequences, aligned to reference genomic sequences using UCSC BLAT program (Dreszer et al., 2012; Kent, 2002), mapped to two different genes at least 750 kb apart (maximum intron size for BLAT) and were less than 50 nt away from a splice site, since genuine fusions tend to have junctions proximal to splice sites (Hahn et al., 2004; Kim et al., 2010). Read-through fusions, which would be excluded by these criteria, were added at a later stage. ChiTaRS also contains fusion transcripts extracted from previous efforts to catalogue fusions, including the TICdb (Novo et al., 2007), dbCrid (Kong et al., 2011), ChimerDB 2.0 (Kim et al., 2010) and Mitelman (Mitelman et al., 2007) databases. Hence, ChiTaRS represents a comprehensive catalogue of fusion transcripts generated by a variety of mechanisms and detected in diverse studies, supported by manual inspection of research articles.

The fusion transcripts were mapped onto known protein sequences in the Ensembl database by translating ChiTaRS genomic alignment coordinates into corresponding protein coordinates. Instances in which genomic coordinates mapped to non-exonic regions such as intronic, UTR or intergenic sequences were discarded in order to isolate fusions affecting protein-coding regions. We further limited our analysis to fusion proteins in which both parents were mapped onto known Ensembl proteins. Full details of the fusion protein mapping are available at http://fusion.d2p2.pro. The gene symbols provided by the original ChiTaRS database for ChiTaRS genomic regions occasionally differ from gene symbols associated with mapped Ensembl proteins, often due to gene symbol aliasing, the use of cDNA alignment accessions instead of gene names by ChiTaRS, or ambiguity associated with the presence of overlapping UTRs and exons in the genome. We default to using gene symbols consistent with the Ensembl framework, and label gene names as ChiTaRS gene names where required. Original ChiTaRS v1 gene names for fusion events are available at http://chitars-old.bioinfo.cnio.es/, are provided along with Ensembl protein mappings at http://fusion.d2p2.pro, and both gene name sets are also presented as part of **Table S1**.

Oncogene and tumour suppressor gene datasets

A list of 239 human oncogenes was retrieved from the Tumour Associated Gene (TAG) database (Chen et al. 2013; from http://www.binfo.ncku.edu.tw/TAG/GeneFinder_chr.php), and 216 protein-coding oncogenes were successfully integrated into our fusion protein database. The TAG database identified both oncogenes and tumour suppressor genes by text-mining the PubMed database and collecting well-studied genes confidently implicated in oncogenesis. 716 human tumour suppressor genes (TSGs) were acquired from the TSGene web resource for tumour suppressor genes (Zhao et al., 2013; from http://bioinfo.mc.vanderbilt.edu/TSGene/) and of these, 626 protein-coding tumour suppressor genes were integrated. TSGene is a comprehensive literature-based knowledgebase of human, mouse and rat tumour suppressor genes presented with supporting functional and expression annotation. Structural feature density calculations were performed on parent proteins across gene sets (parent OG n=75; parent TSG n=161; other parent n=3043; non-parent n=16933; the 3 parent OGs which were also TSGs were classed as TSGs), as well as on included and excluded segments of parent proteins (OG segments n=198; TSG segments n=302; other parent segments n=4837). Minor differences in these sample sizes across certain calculations can result from constraints of overlapping multiple data sets (i.e. accurately mapping between different accession frameworks).

Parent protein mRNA and protein abundance and half-lives

Protein and mRNA abundances were acquired from (Vogel et al., 2010), which experimentally measured absolute protein and mRNA concentrations in the Daoy medulloblastoma cell line using shotgun proteomics and microarrays, respectively. The dataset covers 1,051 proteins, for which protein expression levels (molecules per cell) and mRNA expression levels (arbitrary units) are available. In the 86 cases where measurements were available for multiple isoforms of the same gene, the values were averaged to obtain gene-level measures. The identity of the 3,339 parent genes was overlapped onto the dataset, leading to 374 parents mapped to the abundance data. The remaining genes were regarded as non-parents. Statistical significances of the differences in abundance distributions were quantified using non-parametric Wilcoxon rank-sum tests.

Protein half-lives were acquired from a SILAC (stable isotopic atoms for quantitative mass spectrometry analysis) study in HeLa cells (Boisvert et al., 2012), which quantified protein abundance and turnover. The study identified and quantified 80,098 peptides and mapped these onto 8,041 endogenous HeLa cell proteins (yielding an average of ~10 peptides per protein). Protein turnover rates ranged from under 10 minutes to over a hundred hours, with an average turnover rate of ~20 h. 1,545 parent genes were mapped to the dataset, and the remainder was regarded as non-parent genes. Differences in half-life distributions were quantified as before.

Parent gene participation in oncogenic signaling blocks
To investigate if parent proteins are more likely than expected to participate in cancer signaling processes, 328 gene members of 12 oncogenic 'signaling blocks' were acquired. Signaling blocks were derived from an analysis that integrated a manually curated human signaling network with information on cancer-associated genetically and epigenetically altered genes (Cui et al., 2007). 181 of the provided 'gene names' were recognized and incorporated into the analysis. A contingency table of parent genes against signaling genes was constructed and tested using a chi-squared test of independence.

Protein-protein interaction network datasets
The Wang dataset of binary protein-protein interactions (Wang et al., 2012) consists of 12,500 literature-curated binary interactions taken from 6 databases and 8,000 well-verified high-throughput yeast two-hybrid interactions. In total, 20,614 binary interactions were acquired from the dataset, composed of 7,401 unique genes, of which 1,738 were parent genes. Network centrality measures were calculated (**Experimental Procedures**) for parent and non-parents. Furthermore, an unbiased network of 56,553 protein-protein interactions from 10,961 proteins, derived from affinity purity mass spectrometry experiments (Huttlin et al., 2015), was acquired for further validation of centrality trends (data available at http://wren.hms.harvard.edu/bioplex/).

The tissue specific set of protein-protein interactions (Bossi and Lehner, 2009) was defined by acquiring ~81,000 interactions from 21 sources, with each interaction supported by at least one piece of direct experimental evidence demonstrating physical association, and integrating gene expression data to infer tissue specificity. A given protein-protein interaction was labeled as being present in a tissue if the two genes leading to the proteins are co-expressed above a certain threshold in the cells of that tissue, indicating that they could potentially interact in those cells. The resulting dataset contains 78 tissue-specific PPI networks and 1 "consensus", non-tissue specific network. The consensus network was composed of 8,383 unique genes, of which 1,974 were parent genes and 6,409 non-parents. Network centrality measures were calculated and compared between: 1) parents and non-parents, 2) different categories of parents (OGs, TSGs, and other parents) and 3) parent and non-parent OGs and TSGs. Averaged centrality values for parents and non-parents were also calculated for each cell or tissue type.

Protein-protein interaction network centrality in parent proteins
We first performed network centrality calculations on a non-tissue specific protein-protein interaction network (Wang et al., 2012). Using the igraph R package (http://igraph.org), the (undirected) degree centrality, betweenness centrality (Gursoy et al., 2008) and Kleinberg's hub score (Kleinberg, 2000) were calculated for both parent and non-parent genes/proteins. The degree of each node i in an undirected graph is defined as the number of edges incident upon a node. In PPI networks, this gives the number of interacting partners for a given protein. The degree centrality $C_d(i)$ of a node i is simply its degree, given by

$$C_d(i) = deg(i) = |N(i)|$$

where $N(i)$ is the number of neighbours of node i. Nodes with especially high degree centrality are also called 'hubs'.

The betweenness centrality of a node reflects the number of shortest paths (geodesics) from all nodes to all others that pass through it. Nodes take on high values for betweenness centrality if they lie on a high proportion of paths connecting all other nodes in the graph. Formally, the C_b of a node i is given by:

$$C_b(i) = \sum_{j<k} g_{jk}(i) / g_{jk}$$

where g_{jk} is the number of geodesics connecting nodes j and k, and $g_{jk}(i)$ is the number of geodesics that the node i lies on. Proteins with high betweenness centrality function as connectors within interaction networks and are sometimes referred to as 'bottlenecks'.

Kleinberg's hub score was first developed to rank web searches and identify "authoritative" pages (which are linked to by many other pages) and "hub" pages (which themselves link to many authorities). We calculate the hub score for each protein in the network, which captures each protein's connectedness to high degree proteins. The hub scores of nodes can be defined recursively by referencing authority scores, or using linear algebra notation can be more concisely defined as the principal eigenvector of the matrix $A*A^T$, where A is the adjacency matrix of the graph.

Network centrality of parents detected in cell lines derived from metastatic and primary tumours
All gene fusions that were detected in a recent complete transcriptomic screen of 675 human cancer cell lines (Klijn et al., 2015) were acquired. The screening of fusion candidates procedure involved checking for multiple breakpoint-spanning reads, correcting for read number, confirming their absence in normal tissues, and checking for in-frame status (Klijn et al., 2015). The dataset comprises 2,371 gene fusions formed from 3,161 parent genes. 2,119 fusions had available cell line information, allowing the identification of 220 gene fusions detected in metastatic tumours and the remainder in primary tumours. We mapped 1,641 parent genes to the consensus, non-tissue specific PPI network (**Figure 3A**)(Bossi and Lehner, 2009) and compared centrality metrics between 'primary parent' genes, 'metastatic parent' genes and 'other' genes. Genes were allocated to the 'metastatic' category if the gene formed a fusion that was detected in at least one cell line of metastatic tumour origin and to the 'primary' category if detected only in cell lines of primary tumour origin. All other genes were classed as non-parent genes.

Intrinsic structural disorder in parent proteins
Residue by residue predictions for structural disorder in the human proteome were calculated using IUPred (Dosztányi et al., 2005; http://iupred.enzim.hu/) as in the **Experimental Procedures**. All sequences in the human proteome was downloaded from Ensembl, processed using custom Python scripts, and input into IUPred (using "long" for the length parameter) to generate disorder predictions. The IUPred algorithm is based on analyzing amino acid composition to estimate the likelihood of polypeptides forming stabilizing inter-residue contacts.

Interaction-mediating domain dataset
The INstruct database (Meyer et al., 2013) is composed of a curated protein interactome network annotated to the structural resolution of individual domains. The dataset encompasses 6,585 interactions in human. Binary interactions were curated from popular interaction databases and then filtered to meet strict quality conditions: the interaction must have at least two separate supporting publications and each of these publications must have a binary evidence code. These interactions were then used to reconstruct 3D interaction interfaces by using co-crystal structures in PDB using a validated homology-based interaction interface inference approach. Densities of interaction-mediating domain residues were analyzed as described in the **Experimental Procedures**. Interaction-mediating domain (IMD) retention was calculated using IMD residue densities instead of simply counting the presence of untruncated IMDs due to the functional potential of large portions of retained IMDs even if they are not 100% retained (cf. linear motifs, for which a requirement of full retention was imposed due to their short length).

Analysis of interaction interfaces in parents
Structures of proteins in complex with either proteins, DNA or RNA molecules were obtained from the PDB and PISA database (http://pdbe.org/pisa). Residues at the interface were identified by parsing

PISA XML files using custom bash and Python scripts. Coordinates from PDB structures were corrected and mapped onto UniProt sequences using the Structure Integration with Function, Taxonomy and Sequence (SIFTS) API (Velankar et al., 2013). A total of 173,199 unique interface-forming residues were extracted from 3,125 proteins (97% of interfaces are protein-protein interfaces, 2.2% DNA-protein interfaces, and 0.8% RNA-protein interfaces). PISA residue densities were calculated by counting unique positions and dividing by protein length. Only the unique interface residues were analysed to control for highly studied proteins having more known interactions or complexes. Differences in the distributions of interface-forming PISA residue densities, as well as PISA residue retention in fusion proteins, were analyzed as before. Biological process and protein class enrichments for parent genes which donate 10 or more interface-forming residues to fusion proteins were calculated using PantherDB.

Linear motif datasets

Using the ELM database of 1,410 experimentally validated, manually curated short linear motifs (LMs) in eukaryotes (Dinkel et al., 2014), we tested for enrichment of human LMs in parent proteins. The linear motifs subtypes are: proteolytic cleavage sites, general ligand binding sites, degradation motifs (regions that promote protein polyubiquitylation and proteasomal degradation), docking sites (motifs that recruit an enzyme to a protein region that is not the active site), sites for post-translational modification and sub-cellular targeting sites. We tested for enrichment of LMs in parent proteins compared to other genes, and calculated the retention vs. exclusion of LMs in fusion proteins (see **Experimental Procedures**). The larger set of putative linear motifs was produced using the ANCHOR predictive method (Dosztányi et al., 2009), which is based on estimating the likelihood of a residue being a part of a disordered binding region by combining information on estimated energies (using the same method as the IUPred algorithm) and the local structural environment. Linear motif densities were calculated by counting unique ELM accessions and dividing by protein length. Differences in linear motif densities were assessed across parent gene sets, and across included versus excluded segments. Due to small sample sizes, functional enrichments were reported even if the number of genes in an enriched category was less than 10. Parent proteins which donate ELMs to fusion proteins were assessed for specific functions as before. To expand the linear motif analysis, putative linear motifs in the human proteome were computationally predicted using the ANCHOR resource (Dosztányi et al., 2009), identifying 1,036,282 short protein-binding regions. All predicted motifs were 6 amino acids in length with consistent scores over 0.4 and disorder scores over 0.2, with cut-offs based on a survey of annotated motifs from ELM (Davey et al., 2012). Due to the lower likelihood that partial linear motifs would retain functionality (due to their short length), both ELM and ANCHOR linear motif densities were calculated as the count of unique, untruncated linear motifs per amino acid residue.

Post-translational modification datasets and analysis

The dbPTM (Lu et al., 2013; http://dbptm.mbc.nctu.edu.tw/) database integrates experimental PTMs obtained from eleven public resources (UniProtKB/Swiss-Prot, Phospho.ELM, PHOSIDA, etc.) as well as from manual curation of research articles. PTMcode v2 catalogues pairs of PTMs within or between interacting proteins which are known or predicted to be functionally associated. These associated PTM sites, which were identified using co-evolution and structural distance models, are good candidate sites for regulating protein interactions (Minguez et al., 2014; http://ptmcode.embl.de/) through either their positioning in protein interfaces (Beltrao et al., 2012) or potentially through allosteric mechanisms (Nussinov et al., 2013).

Enrichments of specific types of modification sites were quantified for included and excluded segments as follows: the background PTM type frequencies for all of dbPTM were calculated, as were the PTM type frequencies for included and excluded segments. For PTM types that occurred 50 or more times in dbPTM, included fusion segments, and excluded fusion segments, fold changes were calculated for both included and excluded segments by dividing by background dbPTM frequencies. Normalized fold changes (1 - fold changes) were plotted to visualize enrichments.

Ubiquitination site gain and loss was analysed in oncogenes and tumour suppressor genes. Experimentally validated ubiquitination (UB) sites were gathered by filtering dbPTM. UB sites were mapped onto fusion protein coordinates, and the number of UB sites lost in oncogene parents and the number of UB sites in segments partnering with TSG parents was tabulated and compared to all other parental segments.

Analysis of transcription factor fusions and the expression levels of target genes

A database of all fusion transcripts identified in TCGA samples (Yoshihara et al., 2015)(http://54.84.12.177/PanCanFusV2/) was filtered to identify all fusion events involving transcription factors (TFs), yielding 1131 TF fusions from 818 samples (**Table S10**). The cancer type acting as the largest source of these fusions (BRCA, breast cancer invasive carcinoma; 461 fusions in 297 samples) was used for further analysis. TCGA was queried to identify instances in which Illumina HiSeq 2000 RNAseqV2 data was available for both the fusion-containing BRCA sample and a matched, healthy solid tissue sample. For these 29 instances, the gene-wise Level 3 RSEM normalized expression counts (i.e. upper quartile normalized RSEM count estimates) for each diseased/healthy pair were extracted. Genes with extremely small read counts ($n<10$) were removed to exclude genes with very low expression values. The RNAseq data was merged for each patient-matched diseased and healthy sample, and differential gene expression (DGE) values were calculated as the absolute $\log_2$ fold change between the diseased and healthy samples. For each gene fusion involving a TF in each BRCA sample, the regulatory targets of the TFs were acquired from the TRRUST database (Han et al., 2015). The absolute $\log_2$ fold change metric was chosen in order to simultaneously examine evidence for either up- or down-regulation of TF targets; further, the regulatory link (i.e. either “activation” or “repression”) between TFs and their targets was frequently unknown (Han et al., 2015). DGE values for the TF targets were compared against the corresponding values for all other genes using non-parametric Wilcoxon rank-sum tests in cases where sufficient regulatory targets ($n\geq20$) were available ($n=8$). The resulting p-values were corrected for multiple testing using Holm’s procedure.

We note that TCGA does not (and possibly could not) provide data from cells with a clean genetic background. In addition to a specific fusion, tumour samples contain a variety of other mutations (often in addition to several fusions), and hence the “background” levels of differential expression are likely to be affected by myriad factors. Furthermore, gene expression levels are influenced by a variety of post-transcriptional regulatory mechanisms (e.g. transcript stability). Despite these caveats and within these limitations, we identify evidence of heightened differential expression of target genes of transcription factors involved in fusions in our dataset. This provides support for potential downstream rewiring effects due to the fusion event for some proteins (TFs) in our dataset.

SUPPLEMENTAL REFERENCES

Beltrao, P., Albanèse, V., Kenner, L.R., Swaney, D.L., Burlingame, A., Villén, J., Lim, W.A., Fraser, J.S., Frydman, J., and Krogan, N.J. (2012). Systematic functional prioritization of protein posttranslational modifications. Cell *150*, 413–425.

Benson, D.A., Karsch-Mizrachi, I., Clark, K., Lipman, D.J., Ostell, J., and Sayers, E.W. (2012). GenBank. Nucleic Acids Res. *40*, D48–D53.

Chen, J.-S., Hung, W.-S., Chan, H.-H., Tsai, S.-J., and Sun, H.S. (2013). In silico identification of oncogenic potential of fyn-related kinase in hepatocellular carcinoma. Bioinformatics *29*, 420–427.

Dreszer, T.R., Karolchik, D., Zweig, A.S., Hinrichs, A.S., Raney, B.J., Kuhn, R.M., Meyer, L.R., Wong, M., Sloan, C.A., Rosenbloom, K.R., et al. (2012). The UCSC Genome Browser database: extensions and updates 2011. Nucleic Acids Res. *40*, D918–D923.

Gursoy, A., Keskin, O., and Nussinov, R. (2008). Topological properties of protein interaction networks from a structural perspective. Biochem. Soc. Trans. *36*, 1398–1403.

Hahn, Y., Bera, T.K., Gehlhaus, K., Kirsch, I.R., Pastan, I.H., and Lee, B. (2004). Finding fusion genes resulting from chromosome rearrangement by analyzing the expressed sequence databases. Proc. Natl. Acad. Sci. U. S. A. *101*, 13257–13261.

Kent, W.J. (2002). BLAT--the BLAST-like alignment tool. Genome Res. *12*, 656–664.

Kim, P., Yoon, S., Kim, N., Lee, S., Ko, M., Lee, H., Kang, H., Kim, J., and Lee, S. (2010). ChimerDB 2.0--a knowledgebase for fusion genes updated. Nucleic Acids Res. *38*, D81–D85.

Kleinberg, J. (2000). Navigation in a small world. Nature *406*, 845.

Klijn, C., Durinck, S., Stawiski, E.W., Haverty, P.M., Jiang, Z., Liu, H., Degenhardt, J., Mayba, O.,

Gnad, F., Liu, J., et al. (2015). A comprehensive transcriptional portrait of human cancer cell lines. Nat. Biotechnol. *33*, 306–312.

Kong, F., Zhu, J., Wu, J., Peng, J., Wang, Y., Wang, Q., Fu, S., Yuan, L.-L., and Li, T. (2011). dbCRID: a database of chromosomal rearrangements in human diseases. Nucleic Acids Res. *39*, D895–D900.

Novo, F.J., de Mendíbil, I.O., and Vizmanos, J.L. (2007). TICdb: a collection of gene-mapped translocation breakpoints in cancer. BMC Genomics *8*, 33.

Nussinov, R., Ma, B., Tsai, C.J., and Csermely, P. (2013). Allosteric conformational barcodes direct signaling in the cell. Structure *21*, 1509–1521.

Velankar, S., Dana, J.M., Jacobsen, J., van Ginkel, G., Gane, P.J., Luo, J., Oldfield, T.J., O'Donovan, C., Martin, M.-J., and Kleywegt, G.J. (2013). SIFTS: Structure Integration with Function, Taxonomy and Sequences resource. Nucleic Acids Res. *41*, D483–D489.

Zhao, M., Sun, J., and Zhao, Z. (2013). TSGene: a web resource for tumor suppressor genes. Nucleic Acids Res. *41*, D970–D976.
